# Modular synthesis of dihydro-isoquinolines: palladium-catalyzed sequential C(sp^2^)–H and C(sp^3^)–H bond activation†
†Electronic supplementary information (ESI) available. CCDC 1408591. For ESI and crystallographic data in CIF or other electronic format see DOI: 10.1039/c5sc01482d


**DOI:** 10.1039/c5sc01482d

**Published:** 2015-06-26

**Authors:** Weidong Liu, Qingzhen Yu, Le'an Hu, Zenghua Chen, Jianhui Huang

**Affiliations:** a School of Pharmaceutical Science and Technology , Tianjin University , 92 Weijin Road, Nankai District , Tianjin 300072 , China . Email: jhuang@tju.edu.cn; b Collaborative Innovation Center of Chemical Science and Engineering (Tianjin) , Tianjin 300072 , China; c Tianjin Key Laboratory for Modern Drug Delivery & High-Efficiency , China

## Abstract

An efficient synthesis of dihydro-isoquinolines *via* a Pd–catalyzed double C–H bond [a C(sp^2^)–H and a C(sp^3^)–H bond] activation/annulation (CHAA) reaction is presented. This methodology features a short reaction time, high atom economy (loss of H_2_O only) and the formation of a sterically less favoured tertiary C–N bond. This fast (30 min) and environmentally benign radical C–H activation approach has demonstrated the potential direction for the future design/development of fast and efficient C–H direct functionalization processes.

## Abstract 2

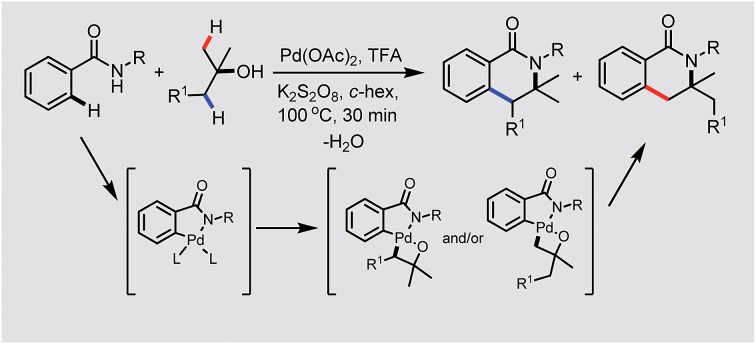
An efficient synthesis of dihydro-isoquinolines *via* a Pd–catalyzed double C–H bond activation/annulation featuring a short reaction time, high atom economy and the formation of a sterically less favoured tertiary C–N bond.

## 

Transition metal catalyzed C–H bond activation has become one of the most studied research areas in the last 15 years.^1^ The activation of C(sp^2^)–H bonds for the syntheses of functionalized arenes and alkenes has been further applied in material science and pharmaceutical sciences.^2^ However, the reactions on the less reactive C(sp^3^)–H bond are still a challenge for synthetic organic chemists. Pioneers like Yu, Gaunt, Kündig, Baudoin, Shi and Rao *et al.* in this area have mainly focused on two typical strategies: (1) Pd-induced oxidative addition onto an aryl halide followed by intramolecular C(sp^3^)–H bond activation to give cyclized product **2**, see Scheme 1-eqn (1);^3^ (2) directed C(sp^3^)–H bond activation of alkane **3** followed by the oxidative addition of an aryl or alkyl halide and reductive elimination for the introduction of alkyl or aryl substituents into the system (Scheme 1-eqn (2)).^4^

**Scheme 1:**
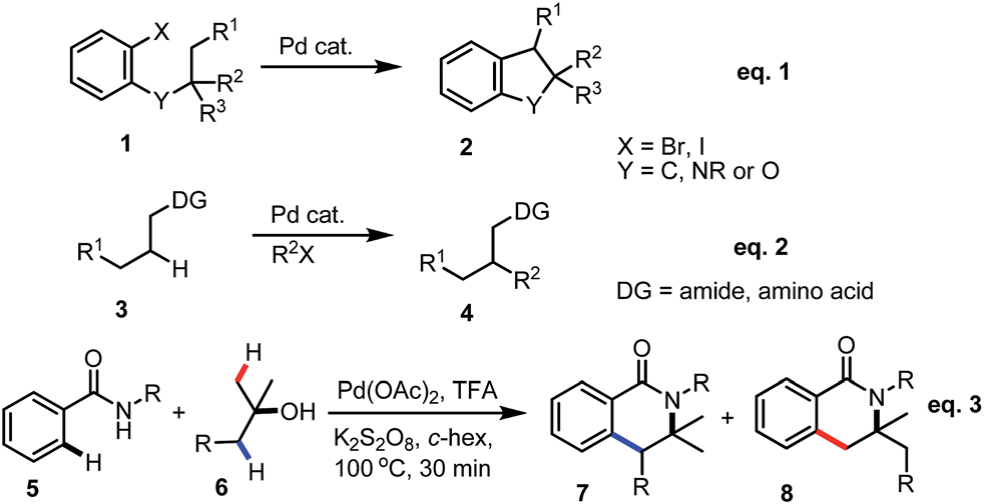
Direct functionalization of the C(sp^3^)–H bond.

However, in both of the strategies (Scheme 1, eqn (1) and eqn (2)), aryl halides or alkyl halides have to be utilized for the direct functionalization of the C(sp^3^)–H bond. Herein, we report our latest discovery of the first direct double activation of C(sp^2^)–H and C(sp^3^)–H bonds for the synthesis of dihydro-isoquinolines under radical processes (Scheme 1-eqn (3)).

Previously, we have demonstrated a type of C–H activation/annulation reaction for the synthesis of hydroxyl isoindolones *via* Pd(iii) or Pd(iv)-catalyzed radical processes.^5^ The possible Pd(iii) intermediate **9** and Pd(iv) species **10** may be formed during the processes (Fig. 1).

**Fig. 1:**
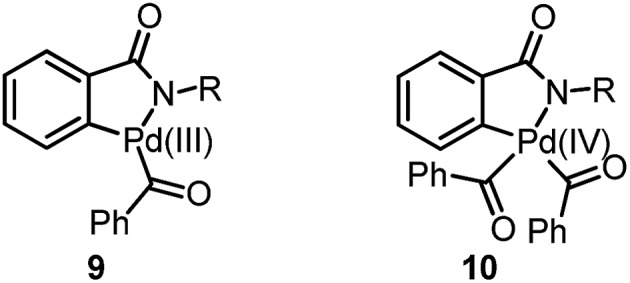
Proposed Pd(iii) and Pd(iv) intermediates.

Inspired by the proposed reactive intermediates, we envisaged a new pathway for the direct C(sp^2^)–C(sp^3^) bond construction *via* a sequential C(sp^2^)–H and C(sp^3^)–H bond activation. As described in Scheme 2, an arene bearing a directing group undergoes a directed C(sp^2^)–H activation to give a Pd(ii) intermediate **12**, upon the radical addition, one or two aliphatic chain(s) (depends on the oxidation state of the Pd centre) could be introduced for the further C(sp^3^)–H bond activation, analogous to the initial step under CMD processes. After the formation of the bicyclic system the highly reactive Pd(iii/iv) species undergoes a reductive elimination to give an intermediate Pd ring followed by further reductive elimination or work up to give the final product **16**.

**Scheme 2:**
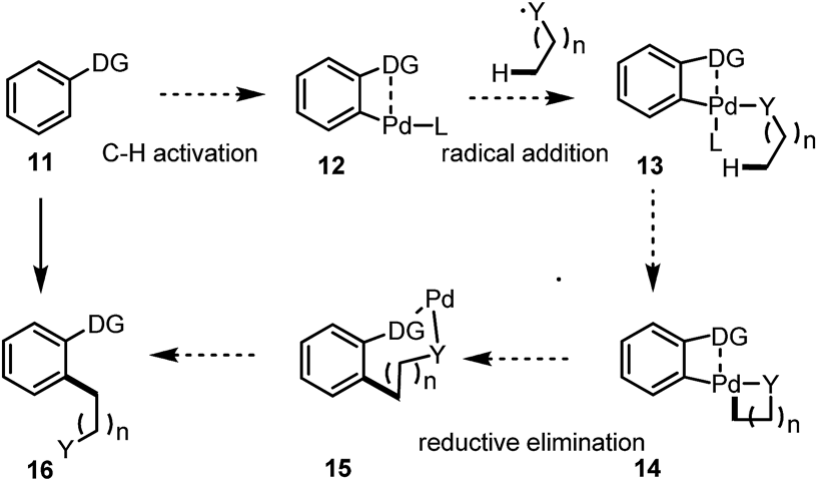
Proposed sequence for the C(sp^2^)–H and C(sp^3^)–H bond activation.

With regard to the proof of concept, *N*-substituted benzamides, acetanilides as well as 2-phenyl pyridines were examined with a variety of radical synthons (possibly formed amide, aldehyde or alcohol radicals) in a combination of radical initiators (AIBN, TBHP *et al.*), in all cases, either unreacted starting material was recovered or hydrolysis was observed for the reactions with benzamides. Even under the reaction conditions we have previously reported for the synthesis of hydroxyisoindolones, we did not observe any of the proposed products (either the directly coupled product or the CHAA product). Interestingly, however, when *N*-methoxyl benzamide **17a**^6^ was treated with 5.0 equivalents of tertiary butyl hydrogen peroxide (TBHP) in the presence of Pd(OAc)_2_ (10 mol%) and 10.0 equivalents of TFA in DCE at 100 °C, our proposed product **18a** was isolated in 5% yield (Scheme 3). The product structure was unambiguously confirmed using X-ray crystallography. With the proof of concept, we carried out our preliminary studies using non-substituted benzamide **17b** as the model for the reaction condition evaluations.

**Scheme 3:**
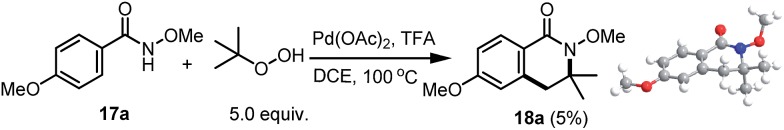
The synthesis of dihydro-isoquinoline **18a** and its X-ray structure.

Based on the preliminary studies, we found that di-*tert*-butyl peroxide (DTBP) was an excellent source for the C–H activations. TFA seemed to be crucial for this type of transformation in order to make the C(sp^3^)–H activation proceed while other acid or base additives did not provide any of our desired product **18b** during our studies. The reaction condition screening focused on the stoichiometry of the acid as well as the screening of the solvents. We found that the acid additive is crucial for this transformation, as demonstrated in ESI Table S1.† In the presence of 20.0 equivalents of TFA, reactions using AcOH and cyclohexane as the solvents gave the corresponding dihydro-isoquinoline **18b** in 69% and 75%, respectively (see ESI, Table S1,† entries 3 and 4), prior to DCE which gave a rather disappointingly low yield. When THF was chosen as the solvent, no product was isolated. Further increasing the amount of TFA used gave rise to a much higher yield and the product was then isolated in 89% yield. The stoichiometry of TFA as the additive was also evaluated and 25 equivalents seemed to be an optimal condition for this transformation (ESI, Table S1, entry 6†). In addition, hexafluorobutyric acid (HFBA) has also shown a good reactivity for the reaction and the desired dihydro-isoquinoline was synthesised in 85% yield (ESI, Table S1, entry 7†).

With the optimal conditions in hand, we have started to evaluate the scope and limitations of this methodology. Neither the electronic nor the steric effects of the substituents on the aromatic ring affected the reactivity. The corresponding dihydro-isoquinolines were successfully prepared. For the synthesis of dihydro-isoquinoline **18b**, we were pleased to find that even under our optimal conditions, the introduction of 3.0 equivalents of benzaldehyde (similar conditions which had been reported previously for the synthesis of hydroxyl isoindolones), our desired product **18b** was isolated in 55% yield together with benzoic acid (27%) and methyl benzoate (18%). Surprisingly, no hydroxyl isodolone was detected from the ^1^H NMR of the crude reaction mixture which indicates that our desired reaction is more favored than the hydroxyl isoindolone formation processes. Benzamides bearing electron deficient substituents at the *para*-position are good substrates and the corresponding dihydro-isoquinolines **18c–18f** were synthesized in good yields. Electron rich benzamides are also tolerated; the products **18g** and **18a** were also facilitated in good yields. Either electron rich or electron deficient substituents at the *ortho*-position would provide dihydro-isoquinolines, such as **18h–18j**, synthesised in 54–94% yields. Reaction of a *meta*-substituted benzamide gave rise to the corresponding product **18k** exclusively in 43% yield which consistently agreed with the selectivity that we have reported previously.^7^ In addition, the reaction with *N*-isopropoxy benzamide was also examined and isoquinoline **18l** was obtained in 75% yield. The reaction of *N*-methyl benzamide also works and the corresponding product **18m** was successfully synthesized, albeit with a low yield (10%). The low yielding reaction is due to the low reaction conversion as most of the starting material was left without having been converted into the dihydro-isoquinoline. Unfortunately, the attempts of the synthesis of *N*-phenyl dihydro-isoquinoline **18n** failed to provide the desired product (Table 1).

**Table 1:** Reaction scope of the benzamides^*a*^

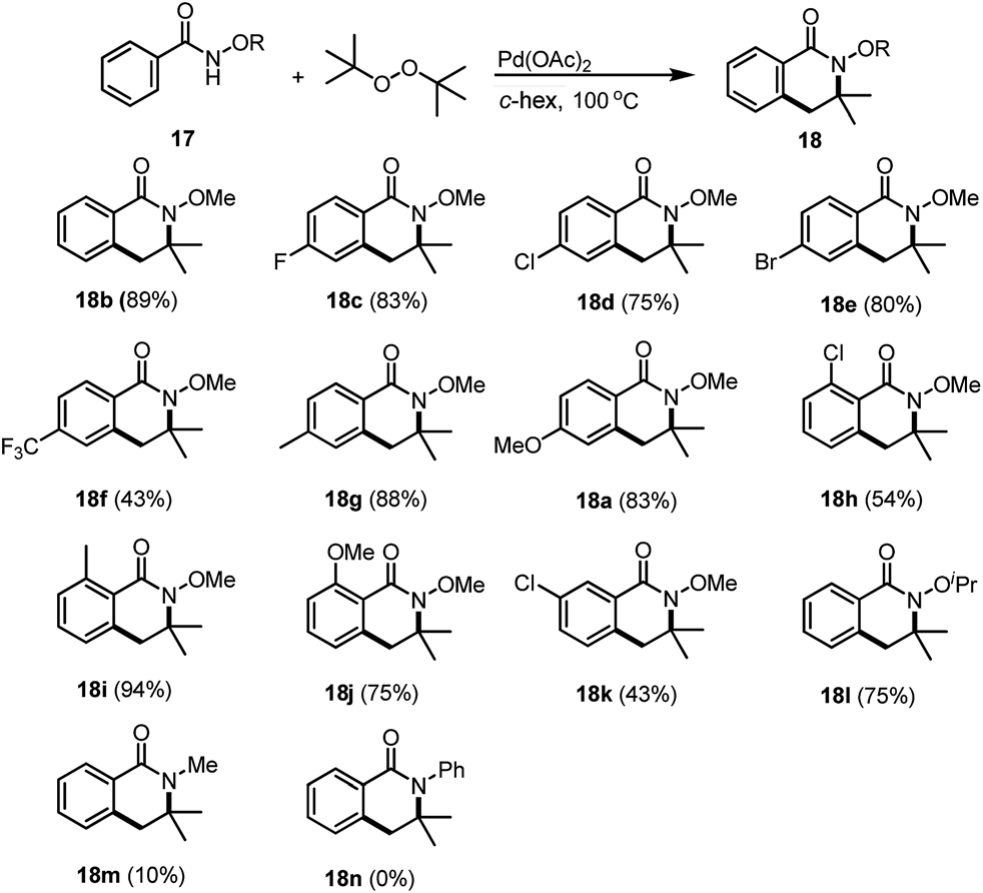

^*a*^Reaction conditions: benzamide **17** (0.2 mmol), DTBP (0.8 mmol), Pd(OAc)_2_ (10 mol%), with TFA (25.0 equiv.) at 100 °C in cyclohexane (0.2 M), 30 min.

Peroxides are not the only efficient coupling partners; we were pleased to find that when the inorganic salt K_2_S_2_O_8_ was employed as the oxidant for the reaction, product **18b** could be synthesised in a similarly good yield (84%), compared to the corresponding reaction with DTBP. The reaction with *N*-methyl benzamide also worked as the desired *N*-methyl dihydro-isoquinoline **18m** was isolated in 10% yield, together with 88% unreacted starting material. Unsymmetrical tertiary alcohols are also tolerated in the reaction, while the reaction of 1,1-dimethyl propanol with benzamide **17b** gave the two regio-isomers **18o** and **18o′** in 63% and 8%, respectively (Table 2). However, the regioselectivity for the syntheses of **18p** and **18q** seemed very low and the isomers **18p** and **18p′** were isolated in 29% and 33%, respectively. Similarly, dihydro-isoquinolines **18o** and **18o′** were formed in a nearly 1 : 1 ratio. The low regio-selectivity in **18p** and **18q** may due to steric hindrance balanced with electronic effects, which had predominantly played crucial roles in the good selectivity for the preparation of **18o** and **18o′**. A similar trend was observed when a more hindered alcohol was utilized and the corresponding dihydro-isoquinolines **18r** and **18r′** were isolated in 32% and 31% yields. Interestingly, when 3-methylpentan-3-ol was employed, only a single regioisomer **18s** was isolated as a 3 : 2 mixture of 2 diastereoisomers (the relative stereochemistry was not determined). These results may indicate that the later C–H activation of an alcohol is more likely to proceed at the more substituted carbon centre. Unfortunately, alcohols **6a–6c** failed to give the corresponding dihydro-isoquinolines, only the background radical hydrolysis products benzoic acid and/or methyl benzoate were formed.

**Table 2:** Reaction scope of the tertiary alcohols^*a*^

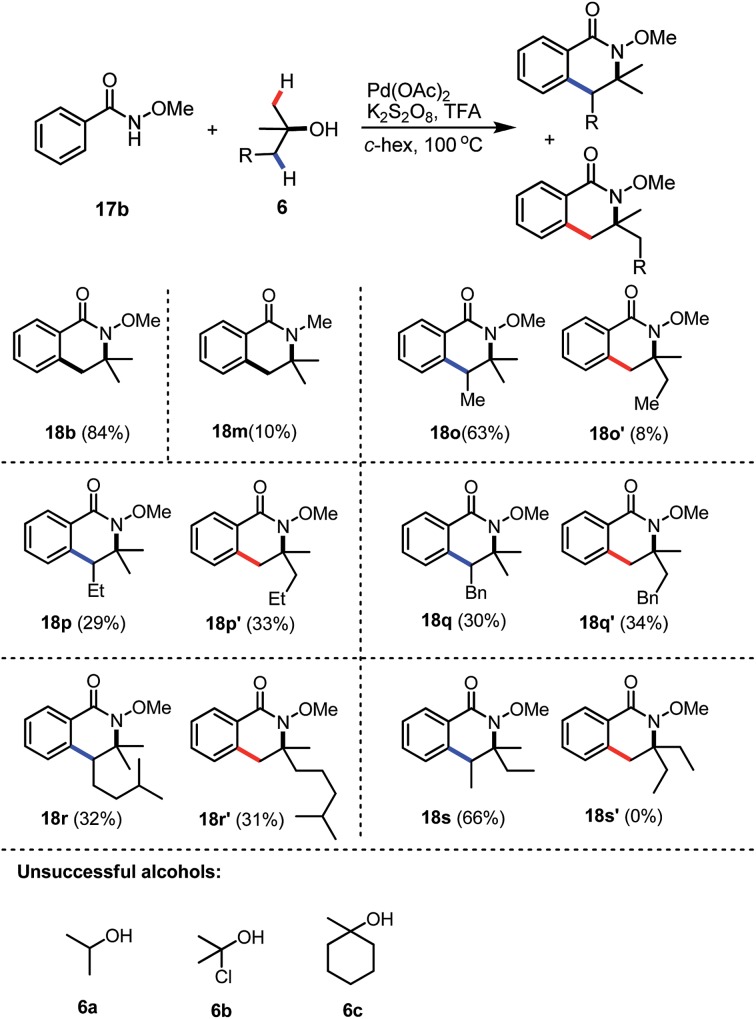

^*a*^Reaction conditions: benzamide **17** (0.2 mmol), tertiary alcohol (0.8 mmol), Pd(OAc)_2_ (10 mol%), K_2_S_2_O_8_ (2.0 equiv.) with TFA (10 equiv.) at 100 °C in cyclohexane (0.2 M), 30 min.

Early this year, Yang has reported the Pd-catalyzed intramolecular tandem aminoalkylation for the activation of C(sp^3^)–H bonds. An intramolecular cyclization reaction of alkenes for the formation of five membered-lactams has been described.^8^ A concern for the product formation could be the simple elimination of a tertiary alcohol for the generation of an alkene, which would be further reacted with benzamide in the presence of the Pd catalyst. However, under our standard conditions, when an alkene was subjected to the reaction, no desired product **18p** was observed, instead only unreacted starting material was recovered in 94% yield after column chromatography (Scheme 4).

**Scheme 4:**
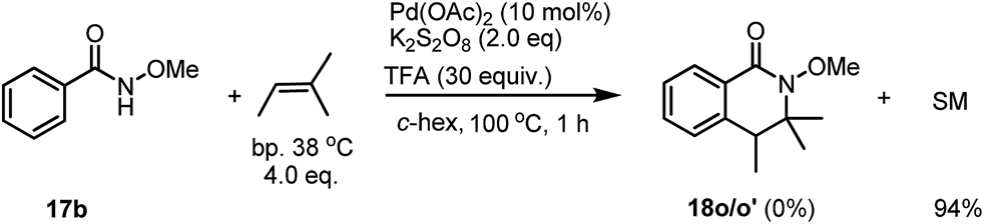
The reaction of benzamide **17b** with an alkene.

The use of a radical scavenger for the reaction was tested; we found that the introduction of 8.0 equivalents of butylated hydroxytoluene (BHT) totally shut down the reactivity. After heating the reaction mixture under standard conditions for one hour, no desired product was observed and only 95% of the unreacted starting material was recovered. Unfortunately, the trapping reaction using TEMPO gave us a complicated reaction mixture with 92% of the starting material recovered (Scheme 5).

**Scheme 5:**
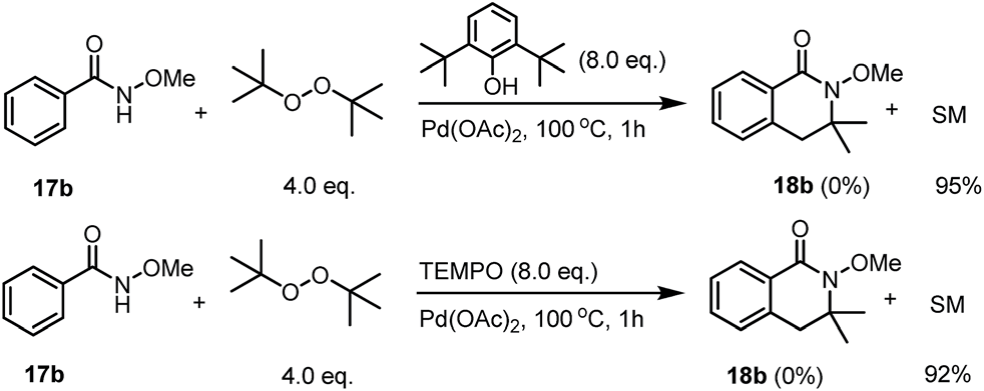
The reactions with radical scavengers.

The reaction using the pre-C–H activated palladacycle **19** was also successful, which may indicate that this reaction may undergo C–H activation of the directed arene first followed by a second C–H activation of the alkyl group after the addition of the radical to the Pd centre (Scheme 6).

**Scheme 6:**
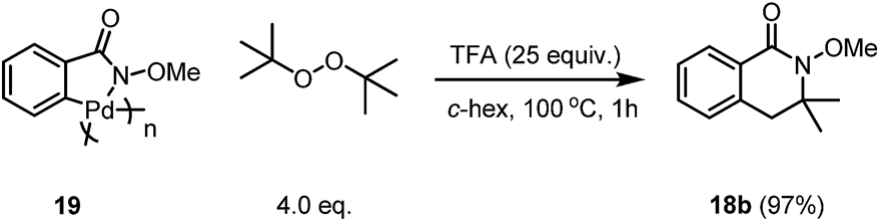
The control experiment with the palladacycle **19**.

To have a better understanding of the reaction mechanism, we set up competitive KIE studies. A 1 : 1 mixture of benzamide **17b** with **17b-5D** under our standard conditions gave a 1.9 : 1 mixture of **18b** and **18b-D5** which showed a primary isotope effect which indicates that the C(sp^2^)–H activation processes may be involved in the selectivity determining step.

Similarly, intramolecular KIE studies using *tert*-butanol-D6 were also carried out. A much higher KIE value of 4.7 : 1 compared to the KIE value 1.9 : 1 for *ortho*-C(sp^2^)–H activation (Scheme 7) was observed which may imply that the C(sp^3^)–H activation processes may be involved in the rate determining step.^9^ Our KIE value for the C(sp^3^)–H activation is comparable with the KIE value of 5.8 : 1 obtained by Baudoin^10^ and Shi's KIE value of 6.2 : 1 (ref. 4*d*) during their studies of C(sp^3^)–H activation reactions (Scheme 8). Both H/D exchange experiments on benzamide **17b-5D** and *tert*-butanol-D6 did not show H/D scrambling under Pd-catalyzed conditions (see in the ESI†), unlike in Rh- and Ru-catalyzed C–H activation processes which are similar to the discoveries that have been discussed previously in our isoquinolone syntheses.^11^ The large KIE value has also proven that the activation of *tert*-butanol is likely to be involved in the reaction pathway instead of the reaction with the possible formed alkene during the reaction which is shown in Scheme 4.

**Scheme 7:**
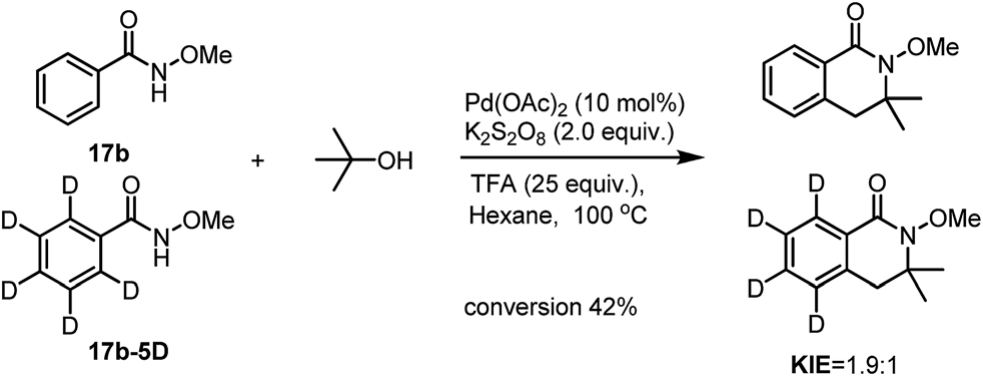
The intermolecular KIE on the C(sp^2^)–H activation processes.

**Scheme 8:**
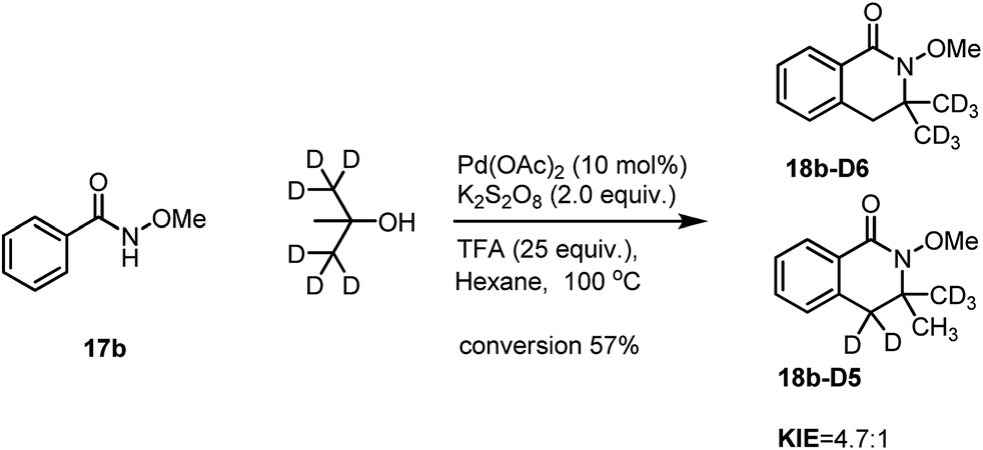
The intramolecular KIE on the C(sp^3^)–H activation.

According to our initial proposal, as well as the evidences we have collected during the mechanistic studies, we have proposed a plausible reaction mechanism based on the Pd(ii)–Pd(iv) cycle for the reaction pathway. The reaction of palladacycle **19** with 1.0 equivalent of tertiary butoxide radical (0.5 equiv. of DTBP) did not provide our desired product in greater than 50% yield (Scheme 9), which may indicate that the reaction needs more radicals for the oxidation of the Pd(ii) species ultimately into Pd(iv), which may be involved in the key reaction mechanism instead of Pd(iii) intermediate.

**Scheme 9:**
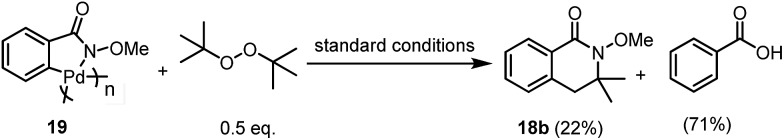
The reaction of palladacycle **19** with a limited radical source.

As we have shown in Fig. 2, starting from benzamide **17b** for instance, the C(sp^2^)–H activation occurred first under acidic conditions to give a palladacycle **A**, (Fig. 2). Under radical conditions, the Pd(iv) species **B** may form, which would give the active precursor **C** after ligand exchange of the *tert*-butanol with trifluoroacetate. The further reaction is involved in a CMD process for the activation of a C(sp^3^)–H bond to form a bicyclic intermediate, **D**. The final product was then formed after a reductive elimination as well as a further elimination of PdO, which had also been proposed by Daves during their studies of *syn*-elimination of PdO.^12^ The ligand exchange would be beneficial for the regeneration of the active Pd(ii) species for the next catalytic cycle.

**Fig. 2:**
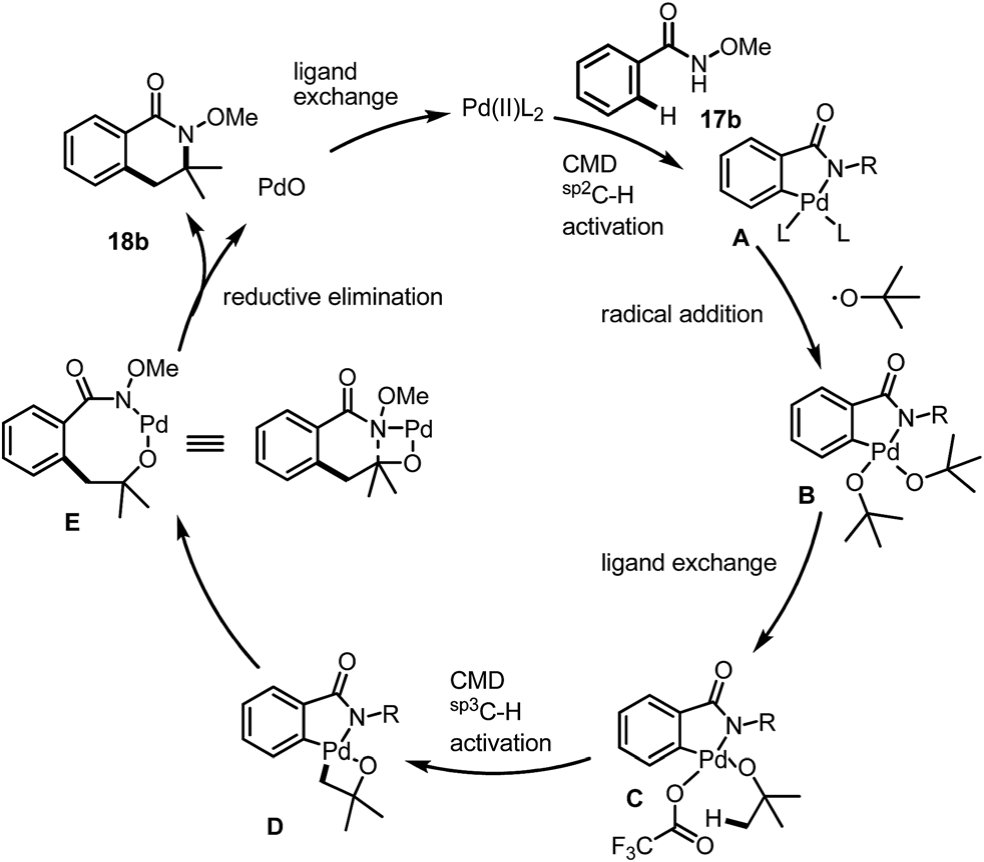
Plausible reaction mechanism.

In conclusion, we have reported a novel approach for the synthesis of a series of useful dihydro-isoquinolines *via* sequential C(sp^2^)–H and C(sp^3^)–H bond activation of benzamides reacting with tertiary alcohols. These novel radical processes featured (1) *high reactivity* – two intermolecular unactivated C–H bonds were directly functionalized, together with a challengeable tertiary amide bond formation; (2) *high efficiency* – reactions were generally fast, in most of the cases reactions were completed within 30 minutes; (3) *modular synthesis* – the heterocyclic ring systems were constructed in a clickable fashion. Detailed mechanistic studies are still in progress.

## Supplementary Material

Supplementary information

Crystal structure data
