# Supplementary material for: Modular synthesis of dihydro-isoquinolines: palladium-catalyzed sequential C(sp2)–H and C(sp3)–H bond activation
Source: Chem Sci. 2015 Jun 26;6(10):5768–72. doi: 10.1039/c5sc01482d (PMC5950195; doi:10.1039/c5sc01482d)
Supplement: Supplementary file 1 [file SC-006-C5SC01482D-s001.pdf]

Electronic Supplementary Material (ESI) for Chemical Science.
This journal is © The Royal Society of Chemistry 2015

Modular synthesis of dihydro-isoquinolines: Palladium-catalyzed sequential C(sp^2)–H and C(sp^3)–H bonds activation

Weidong Liu, Qingzhen Yu, Le'an Hu, Zenghua Chen, Jianhui Huang*

Tianjin Key Laboratory for Modern Drug Delivery & High-Efficiency,
School of Pharmaceutical Science and Technology, Tianjin University,
92 Weijin Road, Nankai District, Tianjin, P. R. China, 300072
email: jhuang@tju.edu.cn

Supporting Information

General Information 2
General Procedures 3
General Procedure A: Synthesis of *N*-methoxybenzamides 3
General Procedure B: Synthesis of *N*-isopropoxybenzamide 3
General Procedure C: Synthesis of *N*-alkoxyl dihydroisoquinolones by amide with peroxide 4
General Procedure D: Synthesis of *N*-alkoxyl dihydroisoquinolones by amide with alcohol 9
References 16
^{1}H and ^{13}C NMR Spectra 17
X-ray Structure and Data of **18a** 36

General Information

Flash chromatography was performed on silica gel 100-200 m. The solvent system used was a gradient of petroleum ether/ethyl acetate, increasing in polarity to ethyl acetate. Thin layer chromatography (TLC) was performed on glass backed plates pre-coated with silica (GF254), which were developed using standard visualizing agents. ^{1}H and ^{13}C NMR spectra were recorded on a 600 MHz or 400 MHz BRUKER AVANCE spectrometer at 25 ^{o}C. ^{1}H: Chemical shifts are reported in ppm with the solvent resonance as the internal standard ($CHCl_3$: δ 7.26 ppm). Data are reported as follows: chemical shift, multiplicity (s = singlet, d = doublet, t = triplet, m = multiplet), integration, coupling constants (*J*) in Hz. ^{13}C NMR spectra were recorded with complete proton decoupling. Chemical shifts are reported in ppm with the solvent resonance as the internal standard ($CDCl_3$: δ 77.0 ppm). Low resolution mass spectra were recorded on Micromass Autospec, operating in Agilent GC-MS operating in either E.I. or C.I mode. High-resolution mass spectra (HRMS) recorded for accurate mass analysis, were performed on either a Q-TOF micro (Bruker Compass Data Analysis 4.0) spectrometer. Melting points were performed on recrystallised solids and recorded on a national standard melting point apparatus and are uncorrected.

General Procedures:

The *N*-methoxybenzamides[1-8] and *N*-methylbenzamide[9] were prepared following literature procedure and the analytical data are agreed with those data which have been reported previously.

General Procedure A: Synthesis of *N*-methoxybenzamides

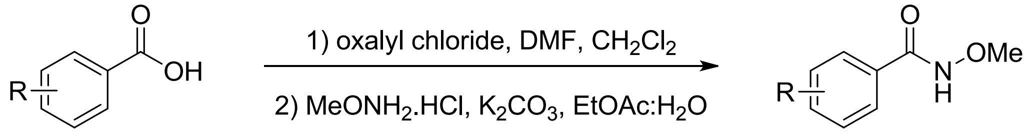

Following same procedure by Guimond *et. al*[1]

To a solution of the carboxylic acid (10 mmol) in CH_2Cl_2 (0.3 M) at 0 °C under N_2 was added dropwise oxalyl chloride (12 mmol) followed by a catalytic amount of DMF (2 drops). The reaction was allowed to stir at room temperature until completion (typically 4h). The solvent was then removed under reduce pressure to afford the corresponding crude acid chloride. Methoxyamine hydrochloride (11 mmol) was added to a biphasic mixture of K_2CO_3 (20 mmol) in a 2:1 mixture of EtOAc:H_2O (0.2 M). The resulting solution was cooled to 0°C followed by addition of a solution unpurified acid chloride in a minimum amount of EtOAc dropwise. The flask containing the acid chloride was then rinsed with additional EtOAc. The reaction was stirring for 4h and slowly warmed up to room temperature. The two layers were separated and extracted with EtOAc (20 mL x 2). The combined organic phase was dried over anhydrous Na_2SO_4, filtered, and evaporated under reduced pressure. The residue was purified by flash column chromatography on silica gel.

General Procedure B: Synthesis of *N*-isopropoxybenzamide

The amide **17l** was prepared by following the procedure reported by A. Nickon *et al*[6], and the analytical data are consistently agreed with those ones are reported in the literature.[7]

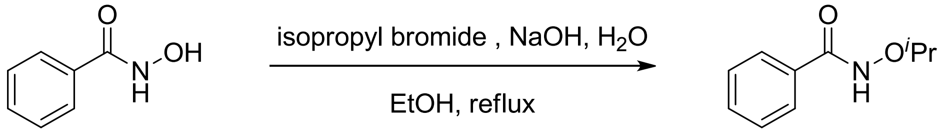

Following similar procedure reported by Nickon *et. al*[6]

Benzohydroxamic acid[8] (3 mmol) was added into a solution of sodium hydroxide (4.5 mmol) in 1.5 mL H_2O, the mixture was warmed to 50 °C until the solids were dissolved, the resulting solution was added to isopropyl bromide (15 mmol) in absolute ethanol (6 mL), and the mixture was heated to reflux for 5 h. After the reaction was completed, the solvents were removed under reduced pressure. The residue was dissolved in EtOAc (20 mL), washed with H_2O (15 mL x 2). The organic layer was dried over anhydrous Na_2SO_4, filtered, and evaporated under reduced pressure. The crude product was purified by flash column chromatography on silica gel.

General Procedure C: Synthesis of *N*-alkoxyl dihydroisoquinolones by amide with peroxide

A solution of amide (0.2 mmol), peroxide (0.8 mmol), $Pd(OAc)_2$ (10 mol%) and TFA (5.0 mmol) in *c*-hex (1.0 mL) was heated in 100 °C under air for 20 min. After the reaction was completed, the reaction mixture was allowed to cool down to room temperature and saturated aqueous $NaHCO_3$ (20 mL) was added. The resulting mixture was extracted with EtOAc (20 mL x 3). The organic layer was dried over anhydrous Na_2SO_4, filtered and the solvent was removed under reduced pressure to provide the crude product. The purification was performed by flash column chromatography on silica gel.

Table 1. Reaction condition screening

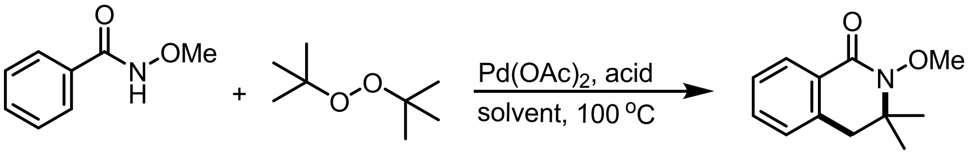

17b **18b**

Entry[a]	Solvent	Acid	Equiv.	Yield
1	DCE	TFA	20	30%
2	THF	TFA	20	0%
3	AcOH	TFA	20	69%
4	Cy	TFA	20	75%
5	Cy	TFA	10	55%
**6**	**Cy**	**TFA**	**25**	**89%**
7	Cy	TFA	30	82%
**8**	**Cy**	**TFA**	**35**	**77%**
9	Cy	HFBA	25	**85%**

[a] Reaction conditions: Benzamide **17b** (0.2 mmol), DTBP (0.8 mmol), $Pd(OAc)_2$ (10 mol%), acid (10-35 equiv.) at 100 ^{o}C in solvent (0.2 M), 20 min-1 h.

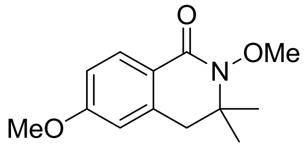

2,6-Dimethoxy-3,3-dimethyl-3,4-dihydroisoquinolin-1(2H)-one (18a)

Following the general procedure C, dihydroisoquinolinone **18a** (39 mg, 83%) was isolated as a white solid: Mp 57-59 ^{o}C; ^{1}H NMR (600 MHz, $CDCl_3$): δ 8.06 (d, *J* = 9.0 Hz, 1H), 6.85 (dd, *J* = 7.2, 1.8 Hz, 1H), 6.64 (d, *J* = 1.2 Hz, 1H), 3.92 (s, 3H), 3.85 (s, 3H), 3.02 (s, 2H), 1.37 (s, 6H); ^{13}C NMR (150 MHz, $CDCl_3$): δ 165.3, 162.9, 138.3, 130.4, 120.9, 112.6, 112.5, 64.5, 61.9, 55.4, 43.5, 24.7; HRMS (ESI) *m/z* calcd for $C_{13}H_{17}NO_3$ (M+Na) 258.1106; found 258.1113.

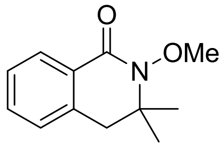

2-Methoxy-3,3-dimethyl-3,4-dihydroisoquinolin-1(2H)-one (18b)

Following the general procedure C, dihydroisoquinolinone **18b** (37 mg, 89%) was isolated as a colorless oil; ^{1}H NMR (600 MHz, $CDCl_3$): δ 8.12 (d, *J* = 7.2 Hz, 1H), 7.46 (t, *J* = 7.2 Hz, 1H), 7.35 (t, *J* = 7.5 Hz, 1H), 7.16 (d, *J* = 7.8 Hz, 1H), 3.94 (s, 3H), 3.08 (s, 2H), 1.39 (s, 6H); ^{13}C NMR (150 MHz, $CDCl_3$): δ 165.0, 136.1, 132.6, 128.2,

127.6, 127.0, 64.5, 62.1, 43.2, 24.8; HRMS (ESI) *m/z* calcd for $C_{12}H_{15}NO_2$ (M+H) 206.1181; found 206.1181.

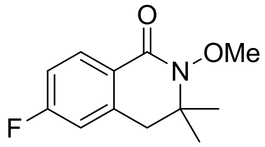

6-Fluoro-2-methoxy-3,3-dimethyl-3,4-dihydroisoquinolin-1(2H)-one (18c)

Following the general procedure C, dihydroisoquinolinone **18c** (37.0 mg, 83%) was isolated as a white solid: Mp 87-89 ^{o}C; ^{1}H NMR (400 MHz, $CDCl_3$): δ 8.13 (dd, J = 8.6, 5.8 Hz, 1H), 7.02 (td, J = 8.6, 2.4 Hz, 1H), 6.85 (dd, J = 8.7, 2.2 Hz, 1H), 3.93 (s, 3H), 3.06 (s, 2H), 1.39 (s, 6H); ^{13}C NMR (100 MHz, $CDCl_3$): δ 166.5, 164.2, 164.0, 138.9 (d, $J_{C\text{-}F}$ = 8.9 Hz), 131.1 (d, $J_{C\text{-}F}$ = 9.5 Hz), 124.6 (d, $J_{C\text{-}F}$ = 2.8 Hz), 114.4 (dd, $J_{C\text{-}F}$ = 18.8, 3.2 Hz), 64.5, 61.9, 43.2, 24.8; HRMS (ESI) *m/z* calcd for $C_{12}H_{14}FNO_2$ (M+H) 224.1087; found 224.1085.

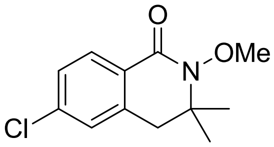

6-Chloro-2-methoxy-3,3-dimethyl-3,4-dihydroisoquinolin-1(2H)-one (18d)

Following the general procedure C, dihydroisoquinolinone **18d** (36 mg, 75%) was isolated as a white solid: Mp 84-86 ^{o}C; ^{1}H NMR (600 MHz, $CDCl_3$): δ 7.98 (d, J = 8.4 Hz, 1H), 7.24 (dd, J = 8.3, 1.5 Hz, 1H), 7.08 (s, 1H), 3.85 (s, 3H), 2.96 (s, 2H), 1.31 (s, 6H); ^{13}C NMR (150 MHz, $CDCl_3$): δ 164.1, 138.6, 137.8, 129.9, 127.6, 127.4, 126.7, 64.5, 61.9, 43.0, 24.8; HRMS (ESI) *m/z* calcd for $C_{12}H_{14}{}^{35}ClNO_2$ (M+H) 240.0791; found 240.0791.

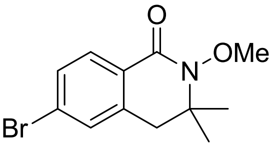

6-Bromo-2-methoxy-3,3-dimethyl-3,4-dihydroisoquinolin-1(2H)-one (18e)

Following the general procedure C, dihydroisoquinolinone **18e** (45 mg, 80%) was isolated as a white solid: Mp 102-104 ^{o}C; ^{1}H NMR (600 MHz, $CDCl_3$): δ 7.98 (d, J = 8.3 Hz, 1H), 7.53 – 7.43 (d, 1H), 7.33 (s, 1H), 3.93 (s, 3H), 3.04 (s, 2H), 1.38 (s, 6H); ^{13}C NMR (150 MHz, $CDCl_3$): δ 164.2, 137.9, 130.6, 130.4, 130.0, 127.2 (9), 127.2 (8),

64.5 (d, J = 2.8 Hz), 62.0, 42.9, 24.8; HRMS (ESI) m/z calcd for $C_{12}H_{14}{}^{79}BrNO_2$ (M+H) 284.0286; found 284.0283.

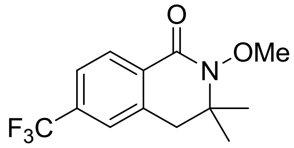

2-Methoxy-3,3-dimethyl-6-(trifluoromethyl)-3,4-dihydroisoquinolin-1(2H)-one (18f)

Following the general procedure C, dihydroisoquinolinone **18f** (24 mg, 43%) was isolated as a white solid: Mp 75-77 °C; ^{1}H NMR (400 MHz, $CDCl_3$): δ 8.26 (d, J = 8.1 Hz, 1H), 7.62 (d, J = 8.1 Hz, 1H), 7.29 (s, 1H), 3.97 (s, 3H), 3.15 (s, 2H), 1.43 (s, 6H).; ^{13}C NMR (100 MHz, $CDCl_3$): δ 163.4, 134.1, 134.0 ($J_{C\text{-}F}$ = 32.4 Hz), 131.3, 128.9, 124.6 ($J_{C\text{-}F}$ = 3.7 Hz), 123.9 ($J_{C\text{-}F}$ = 3.7 Hz), 123.6 ($J_{C\text{-}F}$ = 271.1 Hz), 64.5, 61.9, 43.1, 24.9; HRMS (ESI) m/z calcd for $C_{13}H_{14}F_3NO_2$ (M+H) 274.1055; found 274.1055.

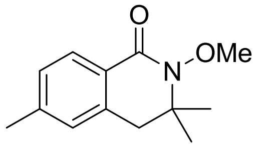

2-Methoxy-3,3,6-trimethyl-3,4-dihydroisoquinolin-1(2H)-one (18g)

Following the general procedure C, dihydroisoquinolinone **18g** (38.5 mg, 88%) was isolated as a colorless oil; ^{1}H NMR (600 MHz, $CDCl_3$): δ 7.99 (d, J = 7.8 Hz, 1H), 7.14 (d, J = 7.8 Hz, 1H), 6.95 (s, 1H), 3.92 (s, 3H), 3.02 (s, 2H), 2.37 (s, 3H), 1.37 (s, 6H); ^{13}C NMR (150 MHz, $CDCl_3$): δ 165.3, 143.2, 136.1, 128.3, 128.2, 127.9, 125.3, 64.4, 62.1, 43.2, 24.8, 21.6; HRMS (ESI) m/z calcd for $C_{13}H_{17}NO_2$ (M+Na) 220.1157; found 220.1158.

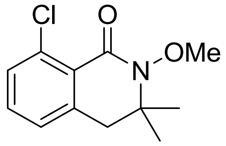

8-Chloro-2-methoxy-3,3-dimethyl-3,4-dihydroisoquinolin-1(2H)-one (18h)

Following the general procedure C, dihydroisoquinolinone **18h** (26 mg, 54%) was isolated as a white solid: Mp 108-110 °C; ^{1}H NMR (400 MHz, $CDCl_3$): δ 7.38 (d, J = 7.9 Hz, 1H), 7.31 (t, J = 7.7 Hz, 1H), 7.07 (d, J = 7.3 Hz, 1H), 3.95 (s, 3H), 3.06 (s, 2H), 1.37 (s, 6H); ^{13}C NMR (100 MHz, $CDCl_3$): δ 162.6, 139.1, 135.6, 132.2, 131.2,

126.7, 124.6, 64.6, 60.6, 44.1, 24.8; HRMS (ESI) *m/z* calcd for $C_{12}H_{14}{}^{35}ClNO_2$ (M+H) 240.0791; found 240.0791.

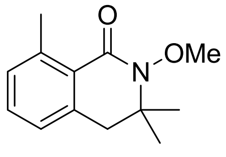

2-Methoxy-3,3,8-trimethyl-3,4-dihydroisoquinolin-1(2H)-one (18i)

Following the general procedure C, dihydroisoquinolinone **18i** (41 mg, 94%) was isolated as a white solid: Mp 60-62 °C; ^1H NMR (400 MHz, $CDCl_3$): δ 7.28 (dd, *J* = 9.6, 5.5 Hz, 1H), 7.12 (d, *J* = 7.6 Hz, 1H), 6.99 (d, *J* = 7.4 Hz, 1H), 3.94 (s, 3H), 3.03 (s, 2H), 2.75 (s, 3H), 1.36 (s, 6H); ^{13}C NMR (100 MHz, $CDCl_3$): δ 166.1, 142.1, 137.4, 131.5, 131.1, 125.9 (1), 125.9 (0), 64.5, 60.9, 44.1, 24.6, 23.2; HRMS (ESI) *m/z* calcd for $C_{13}H_{17}NO_2$ (M+Na) 220.1157; found 220.1158.

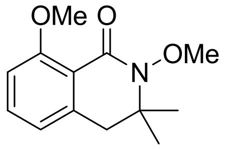

2,8-Dimethoxy-3,3-dimethyl-3,4-dihydroisoquinolin-1(2H)-one (18j)

Following the general procedure C, dihydroisoquinolinone **18j** (35 mg, 75%) was isolated as a white solid: Mp 61-63 °C; ^1H NMR (400 MHz, $CDCl_3$): δ 7.37 (t, *J* = 8.0 Hz, 1H), 6.88 (d, *J* = 8.4 Hz, 1H), 6.72 (d, *J* = 7.4 Hz, 1H), 3.92 (3) (s, 3H), 3.92 (0) (s, 3H), 3.01 (s, 2H), 1.35 (s, 6H); ^{13}C NMR (100 MHz, $CDCl_3$): δ 163.9, 160.8, 139.1, 133.3, 120.0, 116.1, 110.9, 64.4, 60.7, 56.1, 44.1, 24.7; HRMS (ESI) *m/z* calcd for $C_{13}H_{17}NO_3$ (M+Na) 258.1106; found 258.1103.

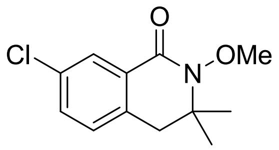

7-Chloro-2-methoxy-3,3-dimethyl-3,4-dihydroisoquinolin-1(2H)-one (18k)

Following the general procedure C, dihydroisoquinolinone **18k** (21 mg, 43%) was isolated as a colorless oil; ^1H NMR (400 MHz, $CDCl_3$): δ 8.12 (d, *J* = 2.2 Hz, 1H), 7.43 (dd, *J* = 8.1, 2.2 Hz, 1H), 7.13 (d, *J* = 8.1 Hz, 1H), 3.95 (s, 3H), 3.06 (s, 2H), 1.40 (s, 6H); ^{13}C NMR (100 MHz, $CDCl_3$): δ 163.7, 134.3, 133.2, 132.5, 129.7, 129.1, 128.1, 64.5, 62.1, 42.7, 24.8; HRMS (ESI) *m/z* calcd for $C_{12}H_{14}{}^{35}ClNO_2$ (M+H) 240.0791; found 240.0791.

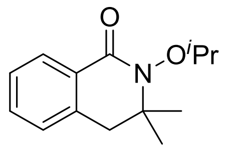

2-Isopropoxy-3,3-dimethyl-3,4-dihydroisoquinolin-1(2H)-one (18l)

Following the general procedure C, dihydroisoquinolinone **18l** (35 mg, 75%) was isolated as a white solid: Mp 44-46 °C; ^{1}H NMR (600 MHz, $CDCl_3$): δ 8.13 (d, J = 7.7 Hz, 1H), 7.44 (t, J = 7.4 Hz, 1H), 7.33 (t, J = 7.5 Hz, 1H), 7.16 (d, J = 7.5 Hz, 1H), 4.46 – 4.32 (m, 1H), 3.09 (s, 2H), 1.36 (s, 6H), 1.32 (d, J = 6.2 Hz, 6H); ^{13}C NMR (150 MHz, $CDCl_3$): δ 167.0, 136.5, 132.3, 128.6, 128.3, 127.5, 126.9, 77.5, 62.1, 43.3, 25.2, 21.2; HRMS (ESI) *m/z* calcd for $C_{14}H_{19}NO_2$ (M+H) 234.1494; found 234.1495.

General Procedure D: Synthesis of *N*-alkoxyl dihydroisoquinolones by amide with alcohol

A solution of amide (0.2 mmol), alcohol (0.8 mmol), $Pd(OAc)_2$ (10 mol%), $K_2S_2O_8$ (108.0 mg, 0.4 mmol) and TFA (2.0 mmol) in *c*-hex (1.0 mL) was heated in 100 °C under air for 20 min. After the reaction was completed, the reaction mixture was allowed to cool down to room temperature and saturated aqueous $NaHCO_3$ (20 mL) was added. The resulting mixture was extracted with EtOAc (20 mL x 3). The organic layer was dried over anhydrous Na_2SO_4, filtered and the solvent was removed under reduced pressure to provide the crude product. The purification was performed by flash column chromatography on silica gel.

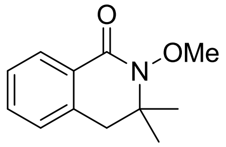

2-methoxy-3,3-dimethyl-3,4-dihydroisoquinolin-1(2H)-one (18b)

Following the general procedure C, dihydroisoquinolinone **18b** (34 mg, 84%) was isolated as a colorless oil; ^{1}H NMR (600 MHz, $CDCl_3$): δ 8.12 (d, J = 7.2 Hz, 1H), 7.46 (t, J = 7.2 Hz, 1H), 7.35 (t, J = 7.5 Hz, 1H), 7.16 (d, J = 7.8 Hz, 1H), 3.94 (s, 3H), 3.08 (s, 2H), 1.39 (s, 6H); ^{13}C NMR (150 MHz, $CDCl_3$): δ 165.0, 136.1, 132.6, 128.2, 127.6, 127.0, 64.5, 62.1, 43.2, 24.8; HRMS (ESI) *m/z* calcd for $C_{12}H_{15}NO_2$ (M+H) 206.1181; found 206.1181.

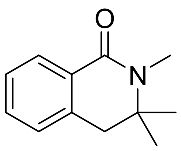

2,3,3-Trimethyl-3,4-dihydroisoquinolin-1(2H)-one (18m)

Following the general procedure C, 2,3,3-trimethyl-3,4-dihydroisoquinolin-1(2H)-one **18m** (4 mg, 10%) was isolated as a colorless oil; ^{1}H NMR (400 MHz, $CDCl_3$): δ 7.99 (d, *J* = 7.7 Hz, 1H), 7.34 (t, *J* = 7.4 Hz, 1H), 7.26 (t, *J* = 7.6 Hz, 1H), 7.07 (d, *J* = 7.4 Hz, 1H), 3.02 (s, 3H), 2.87 (s, 2H), 1.23 (s, 6H); ^{13}C NMR (100 MHz, $CDCl_3$): δ 164.7, 136.1, 131.6, 128.6, 128.2, 127.2, 126.9, 55.9, 42.3, 27.1, 26.1; HRMS (ESI) *m/z* calcd for $C_{12}H_{15}NO_2$ (M+H) 190.1232; found 190.1231.

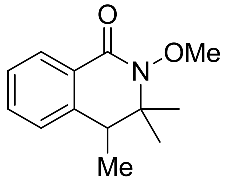

2-Methoxy-3,3,4-trimethyl-3,4-dihydroisoquinolin-1(2H)-one (18o)

Following the general procedure D, dihydroisoquinolinone **18n** (28 mg, 63%) was isolated as a colorless oil; ^{1}H NMR (400 MHz, $CDCl_3$): δ 8.12 (d, *J* = 7.7 Hz, 1H), 7.47 (t, *J* = 7.5 Hz, 1H), 7.34 (t, *J* = 7.5 Hz, 1H), 7.20 (d, *J* = 7.6 Hz, 1H), 3.93 (s, 3H), 2.94 (q, *J* = 7.0 Hz, 1H), 1.37 (s, 3H), 1.33 – 1.32 (m, 6H); ^{13}C NMR (100 MHz, $CDCl_3$): δ 164.3, 142.4, 132.7, 128.2, 127.3, 126.9, 126.8, 64.4, 64.1, 45.0, 29.7, 25.1, 21.7, 17.0; HRMS (ESI) *m/z* calcd for $C_{13}H_{17}NO_2$ (M+Na) 242.1157; found 220.1132.

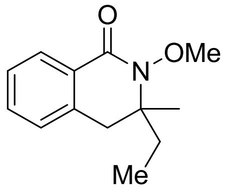

3-Ethyl-2-methoxy-3-methyl-3,4-dihydroisoquinolin-1(2H)-one (18o')

Following the general procedure D, dihydroisoquinolinone **18n'** (3.4 mg, 8%) was isolated as a colorless oil; ^{1}H NMR (400 MHz, $CDCl_3$): δ 8.12 (d, *J* = 7.6 Hz, 1H), 7.44 (t, *J* = 7.0 Hz, 1H), 7.34 (t, *J* = 7.5 Hz, 1H), 7.15 (d, *J* = 7.4 Hz, 1H), 3.94 (s, 2H), 3.06 (s, 2H), 1.87 (m, 1H), 1.66 (m, 1H), 1.40 (s, 3H), 0.90 (t, *J* = 7.5 Hz, 3H); ^{13}C NMR (100 MHz, $CDCl_3$): δ 164.7, 136.0, 132.4, 128.4, 128.1, 127.5, 126.9, 65.0,

64.4, 40.0, 29.7, 22.5, 9.1; HRMS (ESI) *m/z* calcd for $C_{13}H_{17}NO_2$ (M+Na) 242.1157; found 220.1132.

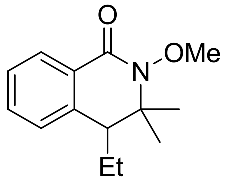

4-Ethyl-2-methoxy-3,3-dimethyl-3,4-dihydroisoquinolin-1(2H)-one (18p)

Following the general procedure D, dihydroisoquinolinone **18o** (13.5 mg, 29%) was isolated as a colorless oil; ^{1}H NMR (400 MHz, $CDCl_3$): δ 8.12 (d, *J* = 7.7 Hz, 1H), 7.44 (td, *J* = 7.5, 1.4 Hz, 1H), 7.35 (td, *J* = 7.6, 1.1 Hz, 1H), 7.11 (d, *J* = 7.5 Hz, 1H), 3.94 (s, 3H), 2.55 (dd, *J* = 9.8, 4.0 Hz, 1H), 1.95 (dtd, *J* = 15.0, 7.5, 3.4 Hz, 1H), 1.66 – 1.56 (m, 1H), 1.51 (s, 3H), 1.21 (s, 3H), 0.80 (t, *J* = 7.5 Hz, 3H); ^{13}C NMR (100 MHz, $CDCl_3$): δ 163.9, 140.2, 131.6, 128.8, 128.3, 127.7, 126.9, 64.2, 64.1, 51.9, 25.7, 23.6, 22.8, 11.8; HRMS (ESI) *m/z* calcd for $C_{14}H_{19}NO_2$ (M+H) 234.1494; found 234.1495.

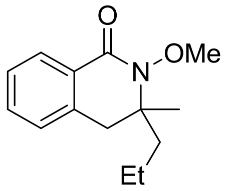

2-Methoxy-3-methyl-3-propyl-3,4-dihydroisoquinolin-1(2H)-one (18p')

Following the general procedure D, dihydroisoquinolinone **18o'** (15 mg, 33%) was isolated as a colorless oil; ^{1}H NMR (400 MHz, $CDCl_3$): δ 8.12 (d, *J* = 7.7 Hz, 1H), 7.44 (t, *J* = 7.4 Hz, 1H), 7.34 (t, *J* = 7.6 Hz, 1H), 7.14 (d, *J* = 7.5 Hz, 1H), 3.94 (s, 3H), 3.07 (s, 2H), 1.83 – 1.72 (m, 1H), 1.63 – 1.52 (m, 1H), 1.42 (s, 3H), 1.31 (m, 2H), 0.86 (t, *J* = 7.3 Hz, 3H); ^{13}C NMR (100 MHz, $CDCl_3$): δ164.7, 136.0, 132.4, 128.4, 128.1, 127.4, 126.9, 64.7, 64.3, 40.7, 39.8, 23.3, 17.9, 14.5; HRMS (ESI) *m/z* calcd for $C_{14}H_{19}NO_2$ (M+H) 234.1494; found 234.1495.

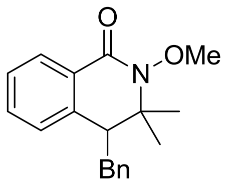

4-benzyl-2-methoxy-3,3-dimethyl-3,4-dihydroisoquinolin-1(2H)-one (18q)

Following the general procedure D, dihydroisoquinolinone **18p** (18 mg, 30%) was isolated as a colorless oil; ^{1}H NMR (400 MHz, $CDCl_3$): δ 8.12 (d, *J* = 6.9 Hz, 1H), 7.26 (t, 1H), 7.19 – 7.13 (m, 3H), 7.08 (td, *J* = 7.5, 1.3 Hz, 1H), 6.77 (m, 2H), 6.21 (d, *J* = 7.5 Hz, 1H), 3.99 (s, 3H), 3.35 (dd, *J* = 12.9, 4.0 Hz, 1H), 2.86 (dd, *J* = 10.9, 4.1 Hz, 1H), 2.67 (dd, *J* = 12.8, 11.1 Hz, 1H), 1.65 (s, 3H), 1.25 (s, 3H); ^{13}C NMR (100 MHz, $CDCl_3$): δ 164.1, 139.4, 139.2, 131.2, 129.6, 129.0, 128.2, 128.1, 127.3, 127.0, 126.2, 64.2, 53.0, 37.5, 29.7, 25.5, 23.4; HRMS (ESI) *m/z* calcd for $C_{19}H_{21}NO_2$ (M+H) 296.1651; found 296.1651.

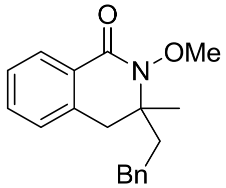

2-methoxy-3-methyl-3-phenethyl-3,4-dihydroisoquinolin-1(2H)-one (18q')

Following the general procedure D, dihydroisoquinolinone **18p'** (20 mg, 34%) was isolated as a colorless oil; ^{1}H NMR (400 MHz, $CDCl_3$): δ 8.12 (d, *J* = 7.7 Hz, 1H), 7.44 (t, *J* = 6.9 Hz, 1H), 7.35 (t, *J* = 7.4 Hz, 1H), 7.23 (d, *J* = 7.5 Hz, 2H), 7.19–7.07 (m, 4H), 3.97 (s, 3H), 3.13 (d, *J* = 5.2 Hz, 2H), 2.67–2.63 (t, *J* = 8.8 Hz, 2H), 2.16 – 2.09 (m, 1H), 1.9 –1.87 (m, 1H), 1.52 (s, 3H); ^{13}C NMR (100 MHz, $CDCl_3$): δ164.6, 141.5, 135.8, 132.5, 128.4, 128.3, 128.2 (1), 128.2 (0), 127.5, 127.0, 126.0, 64.6, 64.5, 41.0, 39.3, 31.1, 23.3.; HRMS (ESI) *m/z* calcd for $C_{19}H_{21}NO_2$ (M+H) 296.1651; found 296.1651.

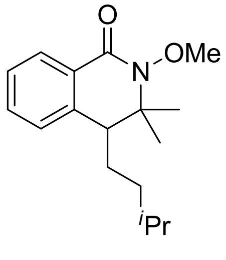

4-isopentyl-2-methoxy-3,3-dimethyl-3,4-dihydroisoquinolin-1(2H)-one (18r)

Following the general procedure D, dihydroisoquinolinone **18q** (17.6 mg, 32%) was isolated as a colorless oil; ^{1}H NMR (400 MHz, $CDCl_3$): δ 8.11 (d, *J* = 7.5 Hz, 1H), 7.43 (dd, *J* = 7.5, 4.2 Hz, 1H), 7.34 (t, *J* = 7.5 Hz, 1H), 7.10 (d, *J* = 7.5 Hz, 1H), 3.95 (s, 3H), 2.59 (dd, *J* = 10.0, 4.0 Hz, 1H), 1.91–1.83 (m, 1H), 1.59–1.55 (m, 1H), 1.50 (s, 3H), 1.21 (s, 3H), 1.14–0.96 (m, 3H), 0.84 (d, *J* = 7.0 Hz, 3H), 0.79 (d, *J* = 7.0 Hz,

3H); ^{13}C NMR (100 MHz, $CDCl_3$): δ 163.9, 140.8, 131.7, 128.5, 128.4, 127.6, 126.9, 64.3, 64.1, 50.8, 36.4, 28.6, 28.2, 25.7, 22.8, 22.3; HRMS (ESI) *m/z* calcd for $C_{17}H_{25}NO_2$ (M+Na) 298.1783; found 298.1780.

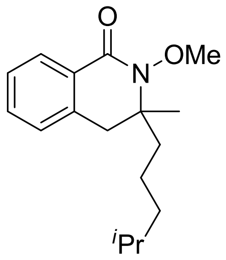

2-methoxy-3-methyl-3-(4-methylpentyl)-3,4-dihydroisoquinolin-1(2H)-one (18r')

Following the general procedure D, dihydroisoquinolinone **18q'** (17.1 mg, 31%) was isolated as a colorless oil; ^{1}H NMR (400 MHz, $CDCl_3$): δ 8.11 (d, J = 7.5 Hz, 1H), 7.44 (t, J = 7.5 Hz, 1H), 7.34 (t, J = 7.5 Hz, 1H), 7.14 (d, J = 7.5 Hz, 1H), 3.94 (s, 3H), 3.07 (s, 2H), 1.84–1.74(m, 2H), 1.51–1.45 (m, 2H), 1.41 (s, 3H), 1.12–1.10 (m, 2H), 0.82 (dd, J = 7.0, 4.0 Hz, 6H), 0.67 (dd, J = 7.0, 4.0 Hz, 1H); ^{13}C NMR (100 MHz, $CDCl_3$): δ 164.7, 136.0, 132.4, 128.4, 128.1, 127.4, 126.9, 64.8, 64.3, 40.6, 39.3, 37.6, 27.8, 23.1, 22.5, 22.3; HRMS (ESI) *m/z* calcd for $C_{17}H_{25}NO_2$ (M+Na) 298.1783; found 298.1784.

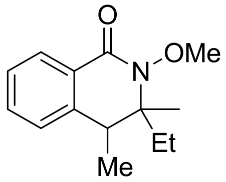

3-ethyl-2-methoxy-3,4-dimethyl-3,4-dihydroisoquinolin-1(2H)-one (18s)

Following the general procedure D, dihydroisoquinolinone **18r** (30.8 mg, 66%) was isolated as a colorless oil; ^{1}H NMR (400 MHz, $CDCl_3$): δ 8.10 (dd, J = 7.5, 4.0 Hz, 1H), 7.45 (td, J = 7.5, 1.4 Hz, 1H), 7.3–7.29 (m, 1H), 7.17 (dd, J = 7.5, 4.0 Hz, 1H), 3.93 (s, 3H), 3.00 (q, J = 7.0 Hz, 1H), 2.00–1.93 (m, 1H), 1.84–1.75 (m, 1H), 1.29 (dd, J = 7.0, 1.5 Hz, 6H), 0.86 (t, J = 7.0 Hz, 3H); ^{13}C NMR (100 MHz, $CDCl_3$): δ 163.6, 142.7, 132.6 (d, J = 3.8 Hz), 128.1 (d, J = 7.1 Hz), 127.4, 127.2, 126.8 (d, J = 4.0 Hz), 67.1, 64.2, 63.7, 41.8, 31.6, 22.7, 14.1; HRMS (ESI) *m/z* calcd for $C_{14}H_{19}NO_2$ (M+H) 234.1494; found 234.1495.

The reaction of benzamide 17b with alkene

A mixture of *N*-methoxylbenzamide (**17b**) (30.2 mg, 0.2 mmol), 2-methylbut-2-ene (0.8 mmol), $Pd(OAc)_2$ (10 mol%), $K_2S_2O_8$ (108.0 mg, 0.4 mmol) and TFA (228.0 mg, 2.0 mmol) in *c*-hex (1.0 mL) was heated at 100 °C for 60 min. The reaction mixture was allowed to cool down to room temperature and saturated aqueous $NaHCO_3$ (20 mL) was added. The resulting mixture was extracted with EtOAc (20 mL x 3). The organic layer was dried over anhydrous Na_2SO_4, filtered and the solvent was removed under reduced pressure to provide the crude product. The purification was performed by flash column chromatography on silica gel to give the unreacted benzamide **17b** (28.4 mg, 94%).

Radical capture experiment with 2,6-di-*tert*-butyl phenol

A solution of *N*-methoxylbenzamide (**17b**) (30.2 mg, 0.2 mmol), DTBP (116.8 mg, 0.8 mmol), $Pd(OAc)_2$ (4.5 mg, 10 mol%) and TFA (684.0 mg, 6.0 mmol) in *c*-hex (1.0 mL) was added 2,6-di-*tert*-butyl phenol (329.6 mg, 1.6 mmol). The mixture was heated at 100 °C under air for 60 min. The reaction mixture was allowed to cool down to room temperature and saturated aqueous $NaHCO_3$ (20 mL) was added. The resulting mixture was extracted with EtOAc (20 mL x 3). The organic layer was dried over anhydrous Na_2SO_4, filtered and the solvent was removed under reduced pressure to provide the crude product. The purification was performed by flash column chromatography on silica gel to give the unreacted benzamide **17b** (28.7 mg, 95%).

Radical capture experiment with TEMPO

A solution of *N*-methoxylbenzamide (**17b**) (30.2 mg, 0.2 mmol), DTBP (116.8 mg, 0.8 mmol), $Pd(OAc)_2$ (4.5 mg, 10 mol%) and TFA (684.0 mg, 6.0 mmol) in *c*-hex (1.0 mL) was added TEMPO (329.6 mg, 1.6 mmol). The mixture was heated in 100 °C under air for 60 min. The reaction mixture was allowed to cool down to room temperature and saturated aqueous $NaHCO_3$ (20 mL) was added. The resulting mixture was extracted with EtOAc (20 mL x 3). The organic layer was dried over

anhydrous Na_2SO_4, filtered and the solvent was removed under reduced pressure to provide the crude product. The purification was performed by flash column chromatography on silica gel to give the unreacted benzamide **17b** (27.8 mg, 92%).

Control experiment with palladacycle 19

A mixture of palladacycle **19** (51.0 mg, 0.2 mmol), DTBP (116.8 mg, 0.8 mmol) and TFA (684.0 mg, 6.0 mmol) in *c*-hex (1.0 mL) was heated at 100 °C for 60 min. The reaction mixture was allowed to cool down to room temperature and saturated aqueous $NaHCO_3$ (20 mL) was added. The resulting mixture was extracted with EtOAc (20 mL x 3). The organic layer was dried over anhydrous Na_2SO_4, filtered and the solvent was removed under reduced pressure to provide the crude product. The purification was performed by flash column chromatography on silica gel to give dihydroisoquinolinone **18b** (39.8 mg, 97%).

Intermolecular competition experiment between benzamides 17b and 17b-5D

A mixture of *N*-methoxylbenzamide (**17b**) (15.1 mg, 0.1 mmol), *N*-methoxyl-2,3,4,5,6-pentadeueriobenzamide (**17b-5D**) (15.6 mg, 0.1 mmol), *t*-BuOH (59.2 mg, 0.8 mmol), $Pd(OAc)_2$ (4.5 mg, 10 mol%), $K_2S_2O_8$ (108.0 mg, 0.4 mmol) and TFA (684.0 mg, 6.0 mmol) in *c*-hex (1.0 mL) was heated at 100 °C for 10 min. The reaction mixture was allowed to cool down to room temperature and saturated aqueous $NaHCO_3$ (20 mL) was added. The resulting mixture was extracted with EtOAc (20 mL x 3). The organic layer was dried over anhydrous Na_2SO_4, filtered and the solvent was removed under reduced pressure to provide the crude product. The purification was performed by flash column chromatography on silica gel to give **18b**/**18b-5D** (21.4 mg, 42%). The ratio of dihydroisoquinolinones **18b**:**18b-5D** in the crude mixture of products was determined by ^{1}H-NMR. The kinetic isotopic effect of this reaction was thus determined to be $kH/kD = 1.9$.

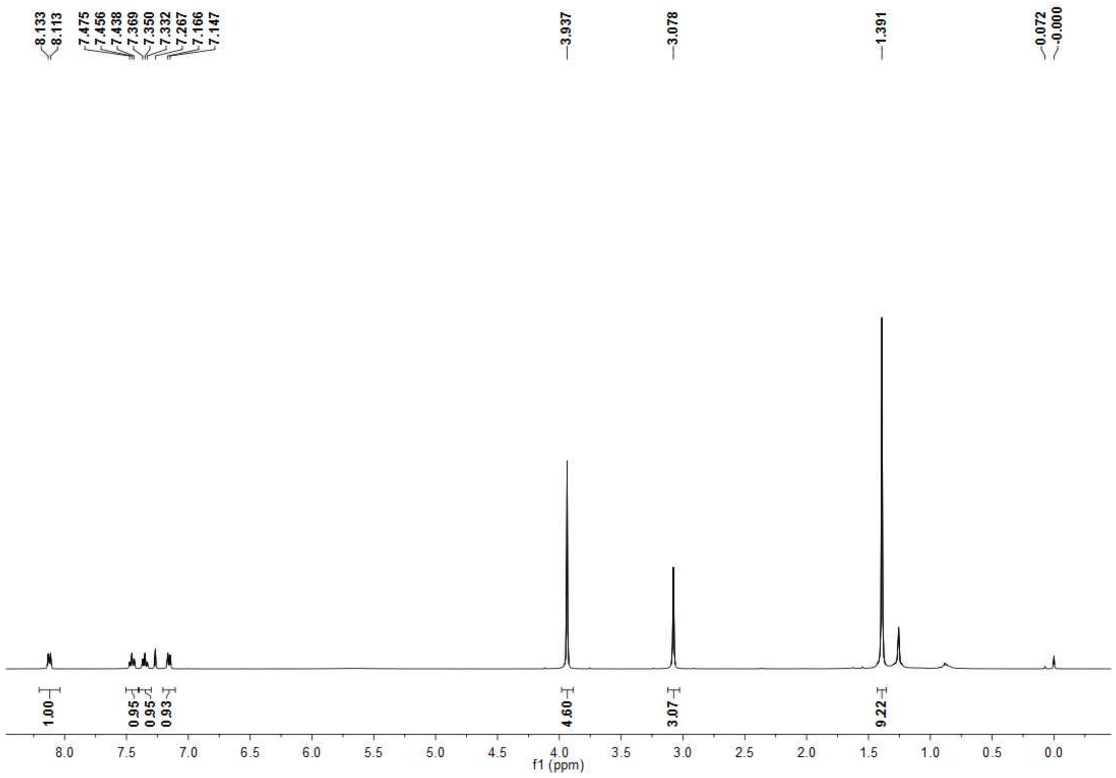

Intramolecular competition experiment with tertiary alcohol 6a-D6

A mixture of *N*-methoxylbenzamide (**17b**) (30.2 mg, 0.2 mmol), *t*-BuOH-**D6** (**[D6]-6a**) (59.2 mg, 0.8 mmol), $Pd(OAc)_2$ (4.5 mg, 10 mol%), $K_2S_2O_8$ (108.0 mg, 0.4 mmol) and TFA (684.0 mg, 6.0 mmol) in *c*-hex (1.0 mL) was heated at 100 °C for 15 min. The reaction mixture was allowed to cool down to room temperature and saturated aqueous $NaHCO_3$ (20 mL) was added. The resulting mixture was extracted with EtOAc (20 mL x 3). The organic layer was dried over anhydrous Na_2SO_4, filtered and the solvent was removed under reduced pressure to provide the crude product. The purification was performed by flash column chromatography on silica gel to give **18b-D6**/**18b-D5** (24.0 mg, 57%). The ratio of dihydroisoquinolinones **18b-D6**:**18b-D5** in the crude mixture of products was determined by ^1H-NMR. The kinetic isotopic effect of this reaction was thus determined to be $kH/kD = 4.7$.

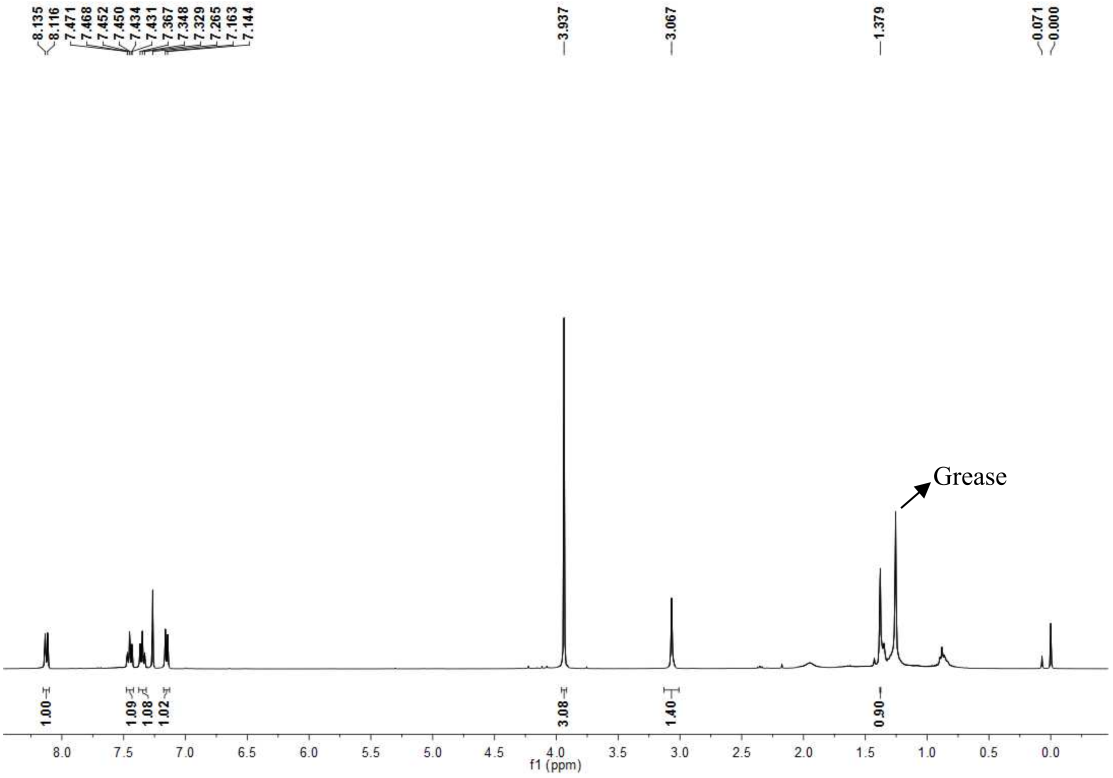

H/D exchange experiments:

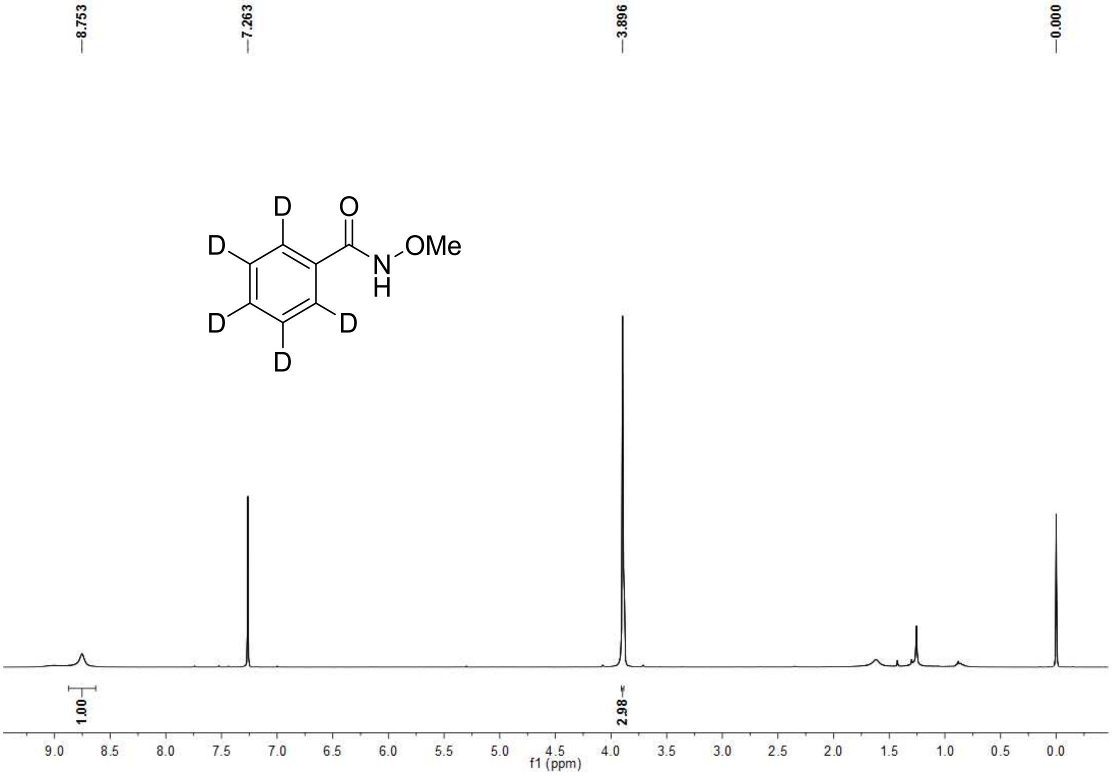

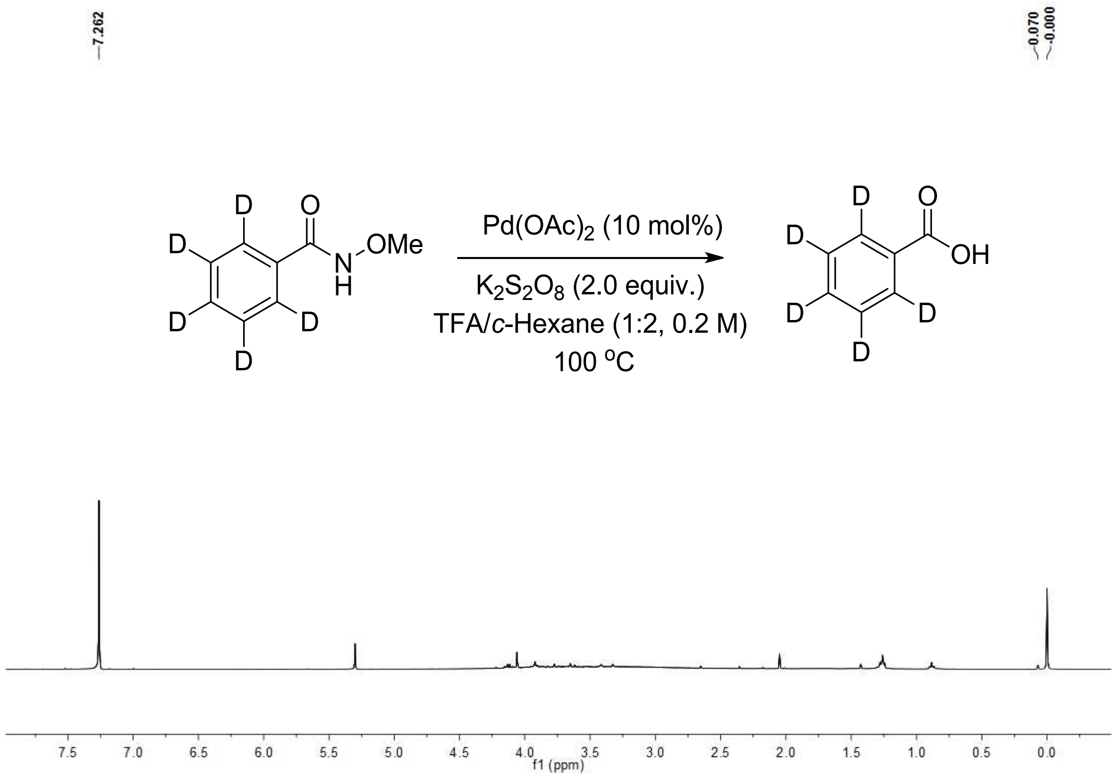
7.262
0.070
-0.000
Pd(OAc)2 (10 mol%)
K2S2O8 (2.0 equiv.)
TFA/c-Hexane (1:2, 0.2 M)
100 oC
f1 (ppm)

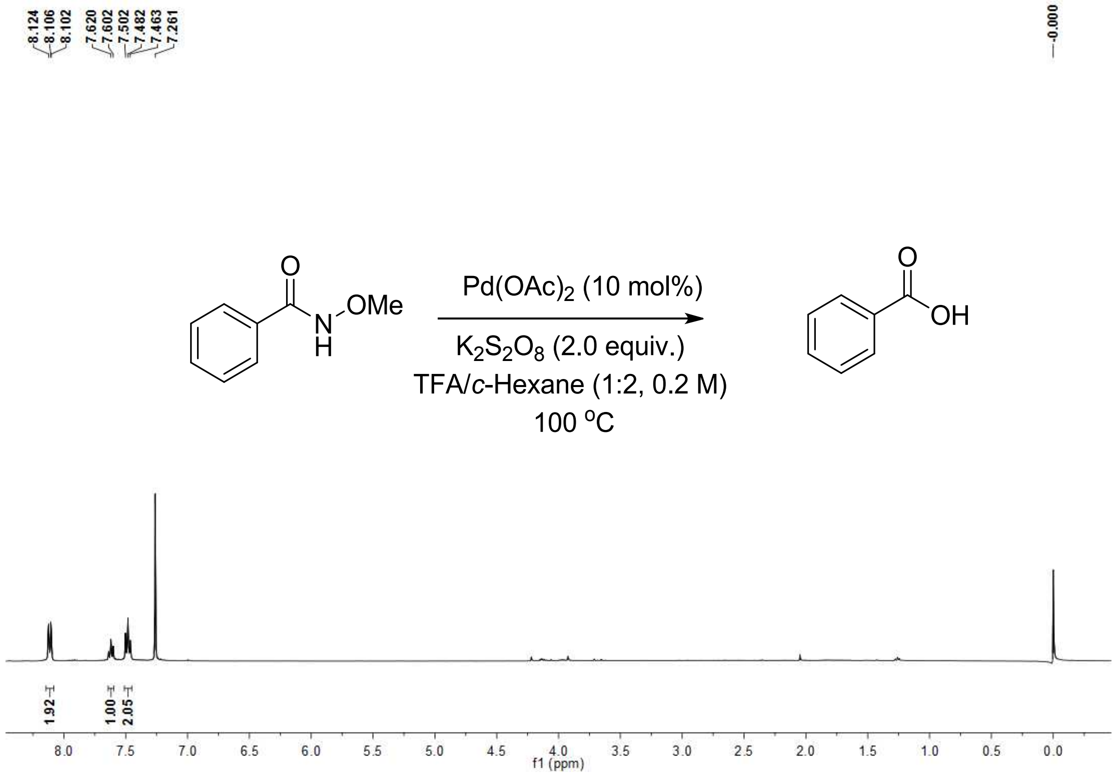
8.124
8.106
8.102
7.620
7.602
7.502
7.482
7.463
7.261
-0.000
Pd(OAc)2 (10 mol%)
K2S2O8 (2.0 equiv.)
TFA/c-Hexane (1:2, 0.2 M)
100 oC
1.92
1.00
2.05
f1 (ppm)

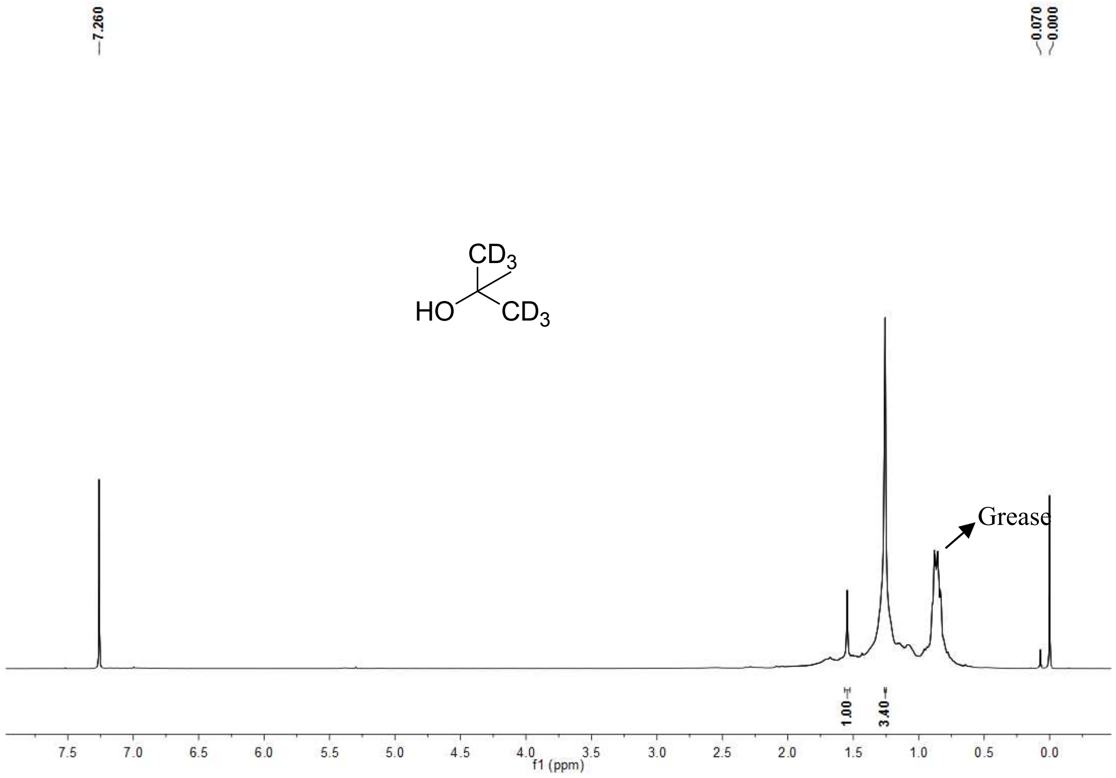

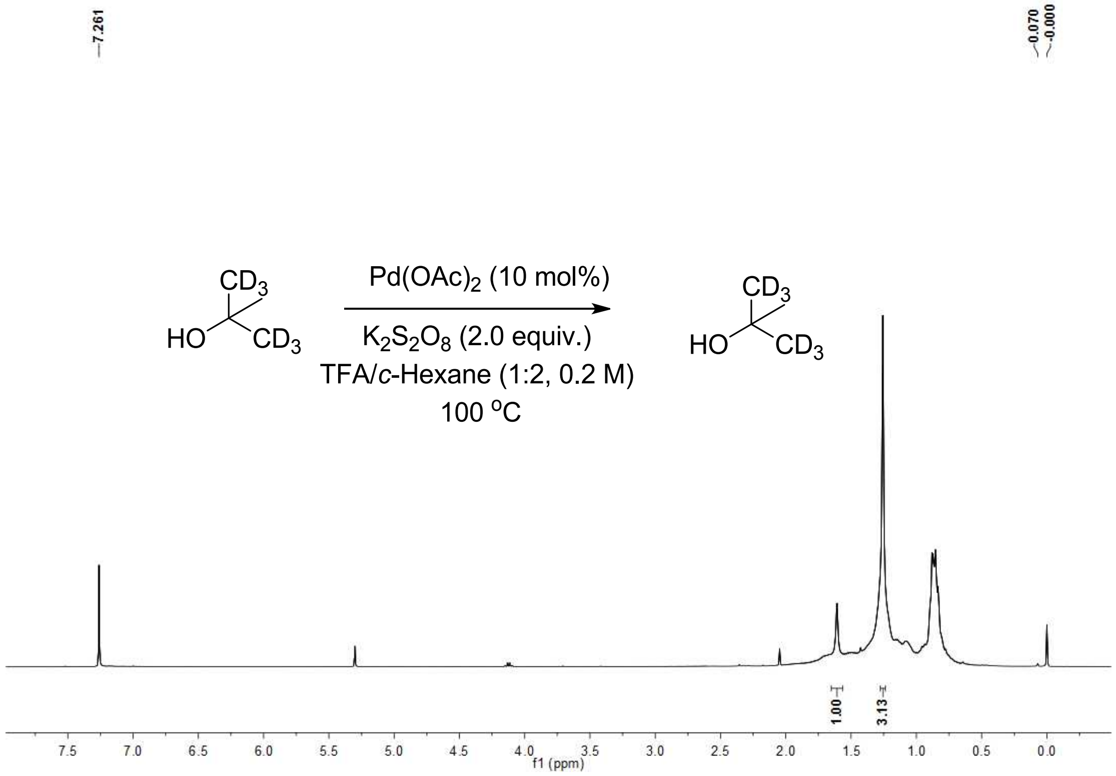

References:

1. N. Guimond, C. Gouliaras and K. Fagnou, *J. Am. Chem. Soc.*, 2010, **132**, 6908.
2. Z. W. Chen, Y. Z. Zhu and J. Yu. Zheng, *J. Org. Chem.*, **2014**, *79*, 10988−10998.

3. Semenov, *et al*, *Zhurnal Organicheskoi Khimii*, **1979**, *15*, 2069.

4. T. Kukosha, N. Trufilkina and M. Katkevics, *et al*, *Synthesis*, **2012**, *44*, 2413–2423.

5. H. B. Zhong, D. Yang, S. Q. Wang and J. H. Huang, *Chem. Commun.*, **2012**, *48*, 3236.

6. N. N. Zhang, B. Y. Li, H. B. Zhong and J. H. Huang, *Org. Biomol. Chem.*, **2012**, *10*, 9429.

^{1}H and ^{13}C NMR Spectra

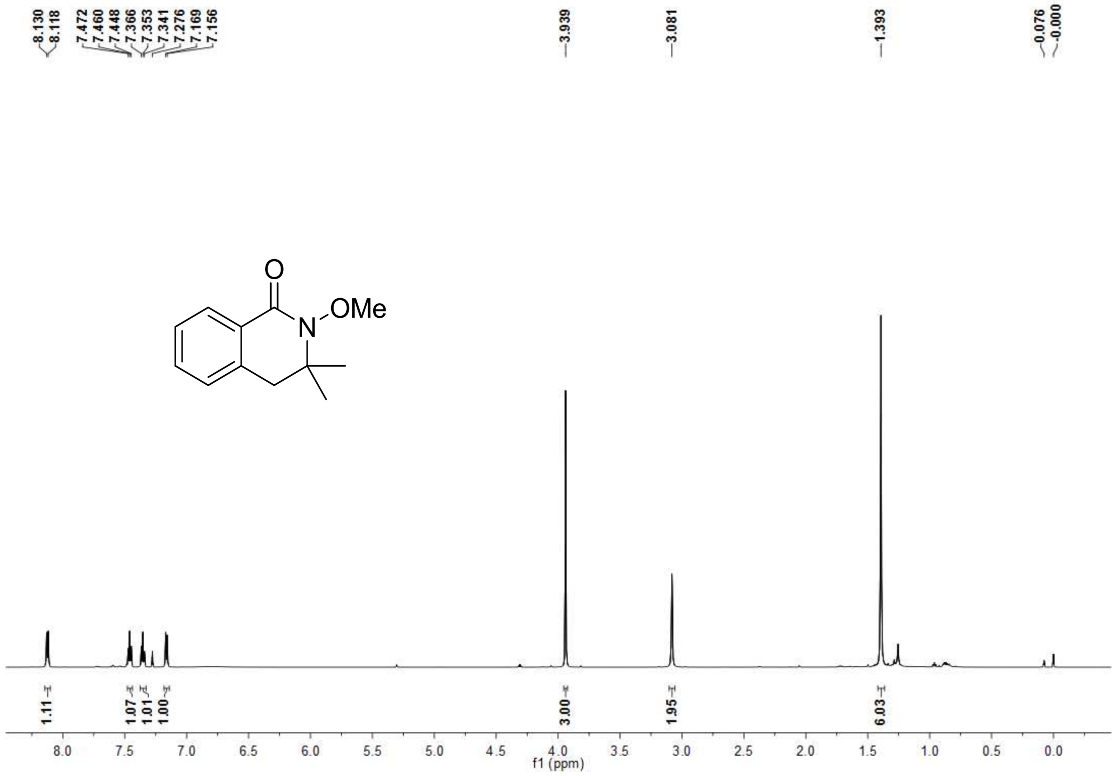

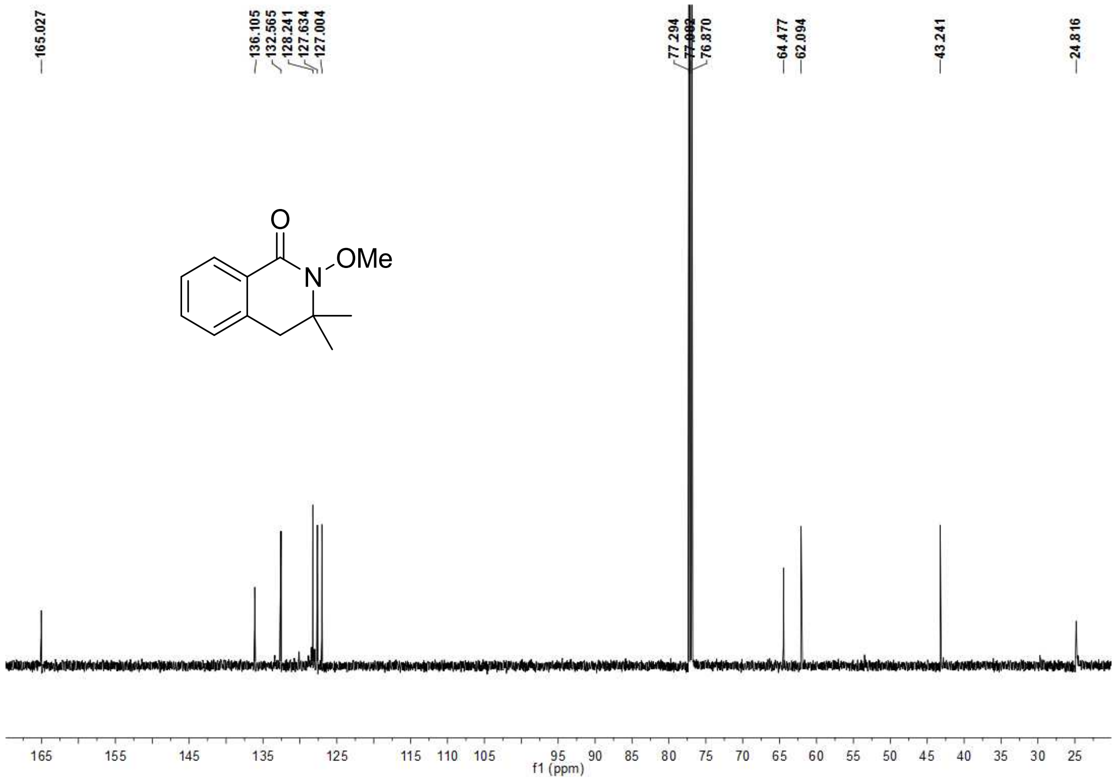

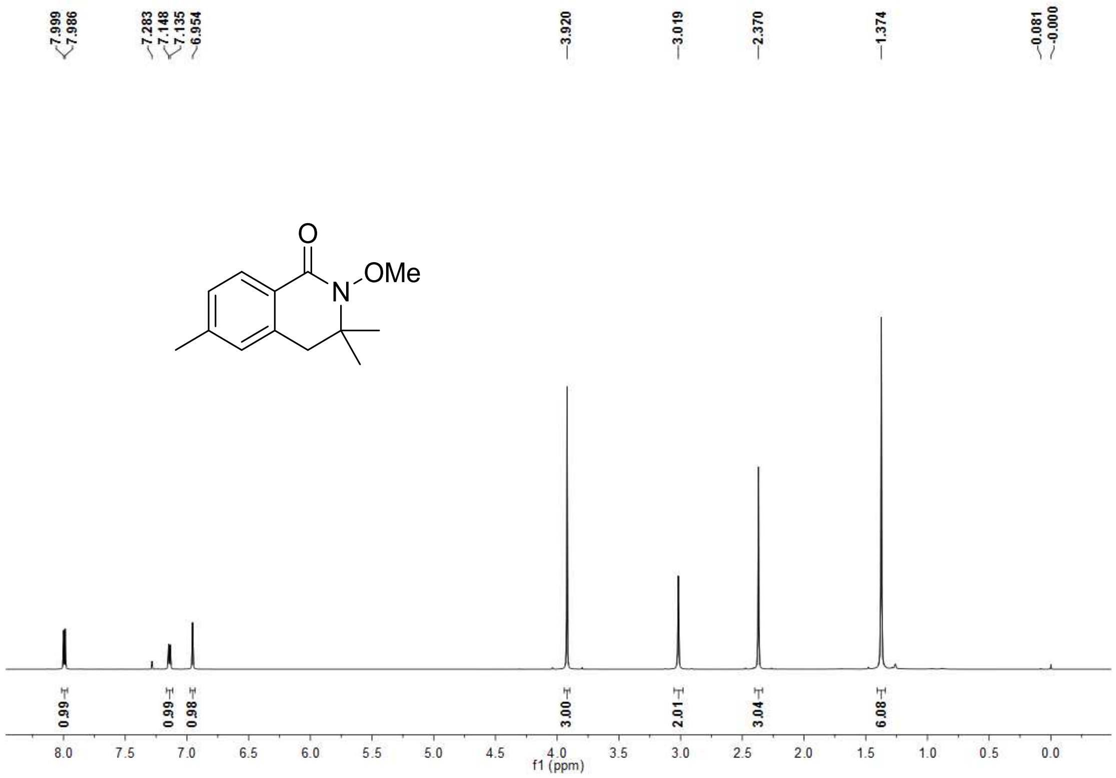
7.999
7.986
7.283
7.148
7.135
6.954
3.920
3.019
2.370
1.374
0.081
-0.000
O
N
OMe
0.99
0.99
0.98
3.00
2.01
3.04
6.08
f1 (ppm)

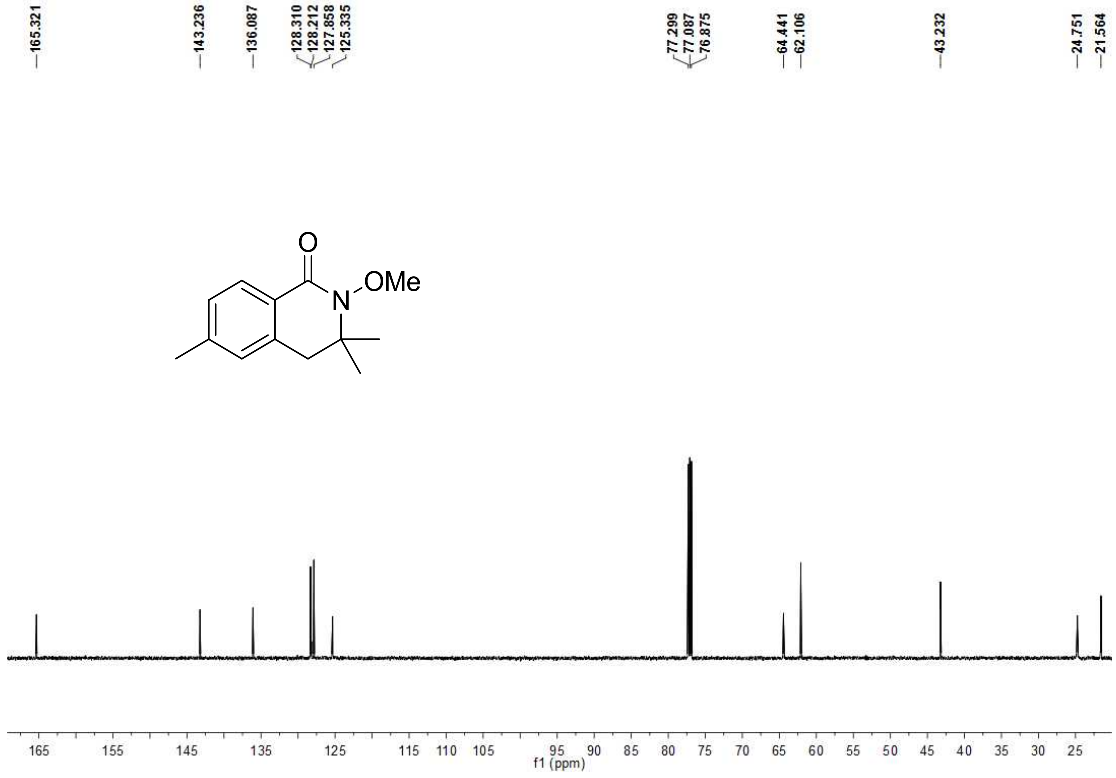
165.321
143.236
136.087
128.310
128.212
127.858
125.335
77.299
77.087
76.875
64.441
62.106
43.232
24.751
21.564
O
N
OMe
f1 (ppm)

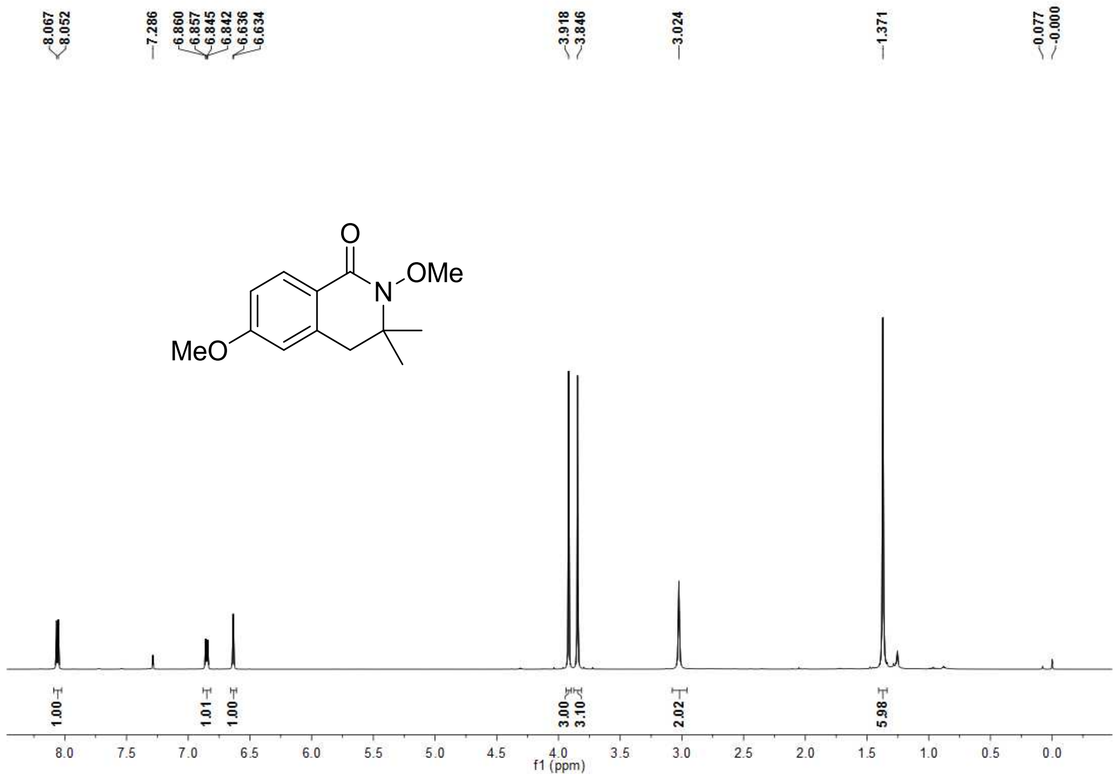
8.067
8.052
7.286
6.860
6.857
6.845
6.842
6.636
6.634
3.918
3.846
3.024
1.371
0.077
-0.000
O
N
OMe
MeO
1.00
1.01
1.00
3.00
3.10
2.02
5.98
8.0 7.5 7.0 6.5 6.0 5.5 5.0 4.5 4.0 3.5 3.0 2.5 2.0 1.5 1.0 0.5 0.0
f1 (ppm)

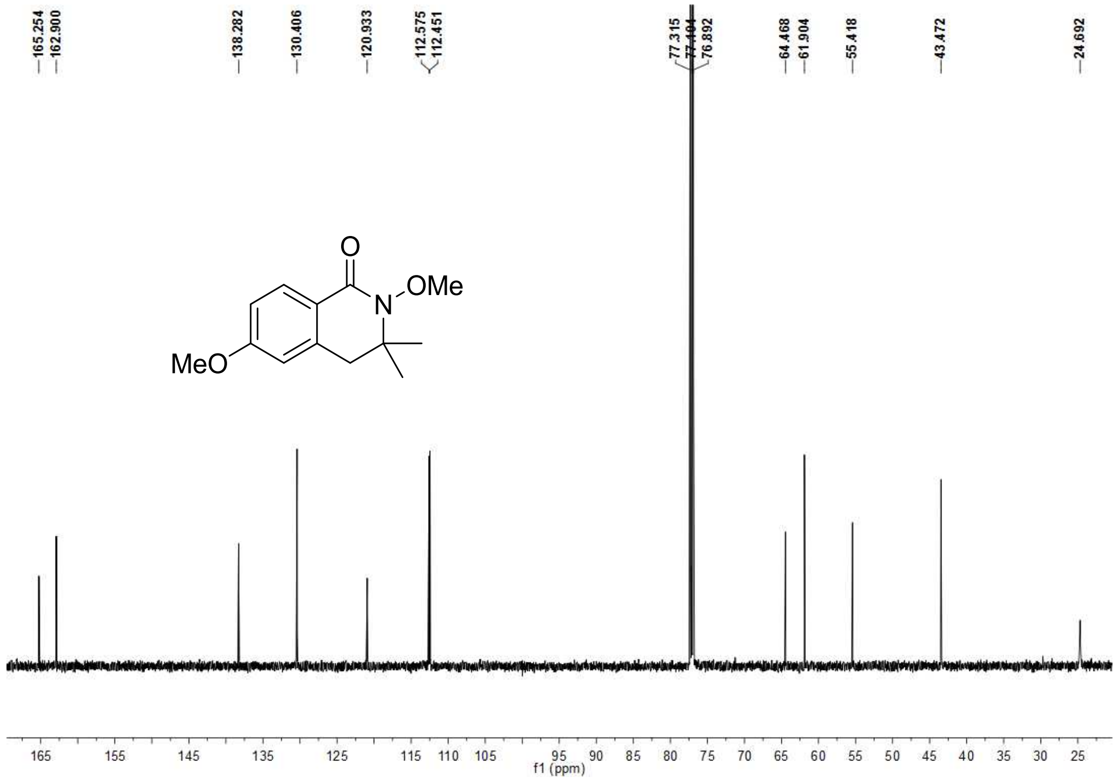
165.254
162.900
138.282
130.406
120.933
112.575
112.451
77.315
76.892
64.468
61.904
55.418
43.472
24.692
O
N
OMe
MeO
165 155 145 135 125 115 110 105 95 90 85 80 75 70 65 60 55 50 45 40 35 30 25
f1 (ppm)

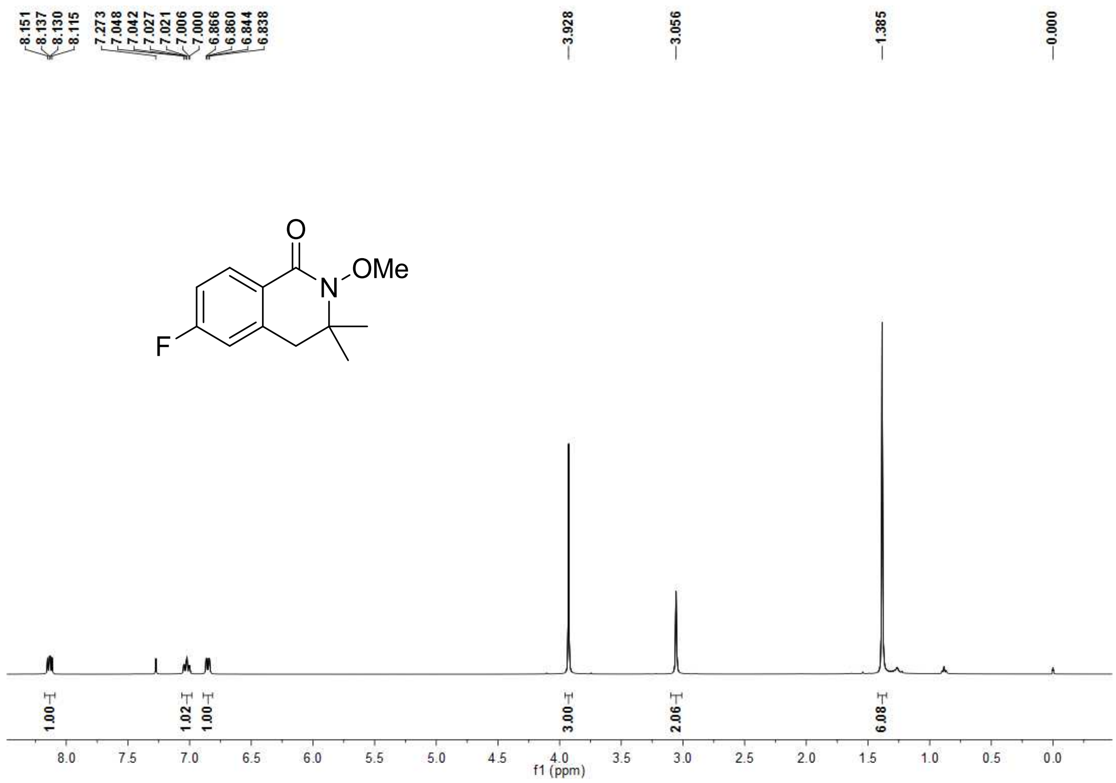
8.151
8.137
8.130
8.115
7.273
7.048
7.042
7.027
7.021
7.006
7.000
6.866
6.860
6.844
6.838
3.928
3.056
1.385
0.000
O
N
OMe
F
1.00
1.02
1.00
3.00
2.06
6.08
f1 (ppm)

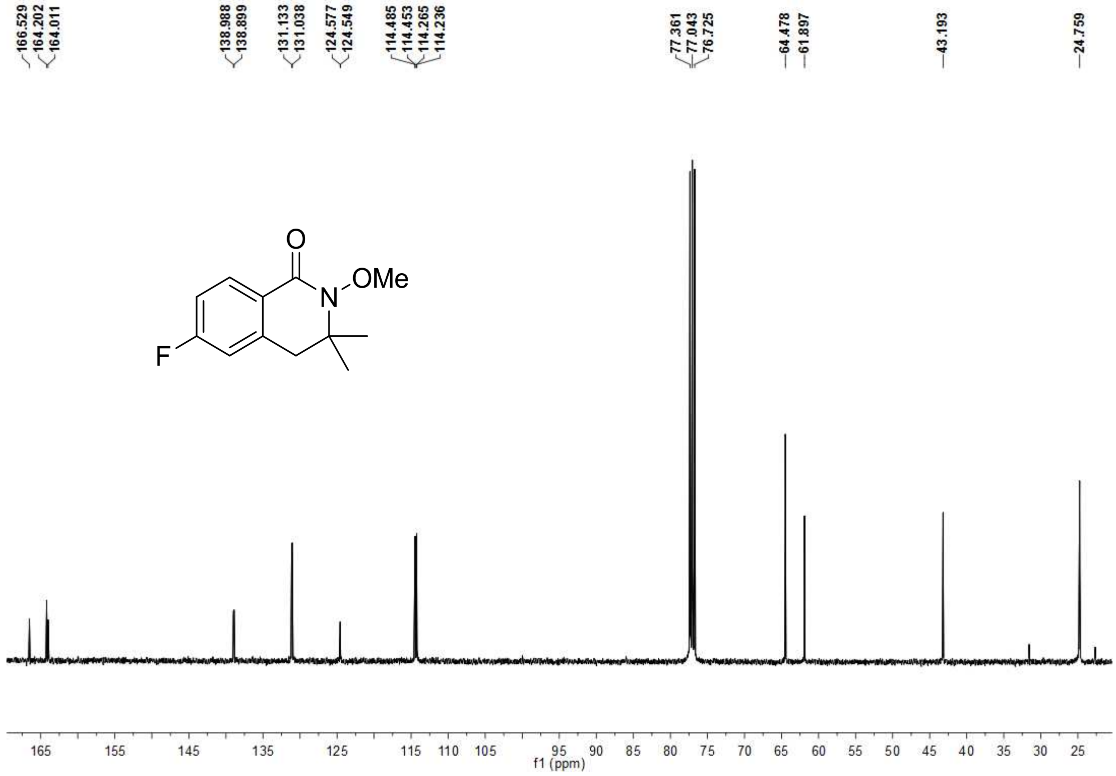
166.529
164.202
164.011
138.988
138.899
131.133
131.038
124.577
124.549
114.485
114.453
114.265
114.236
77.361
77.043
76.725
64.478
61.897
43.193
24.759
O
N
OMe
F
f1 (ppm)

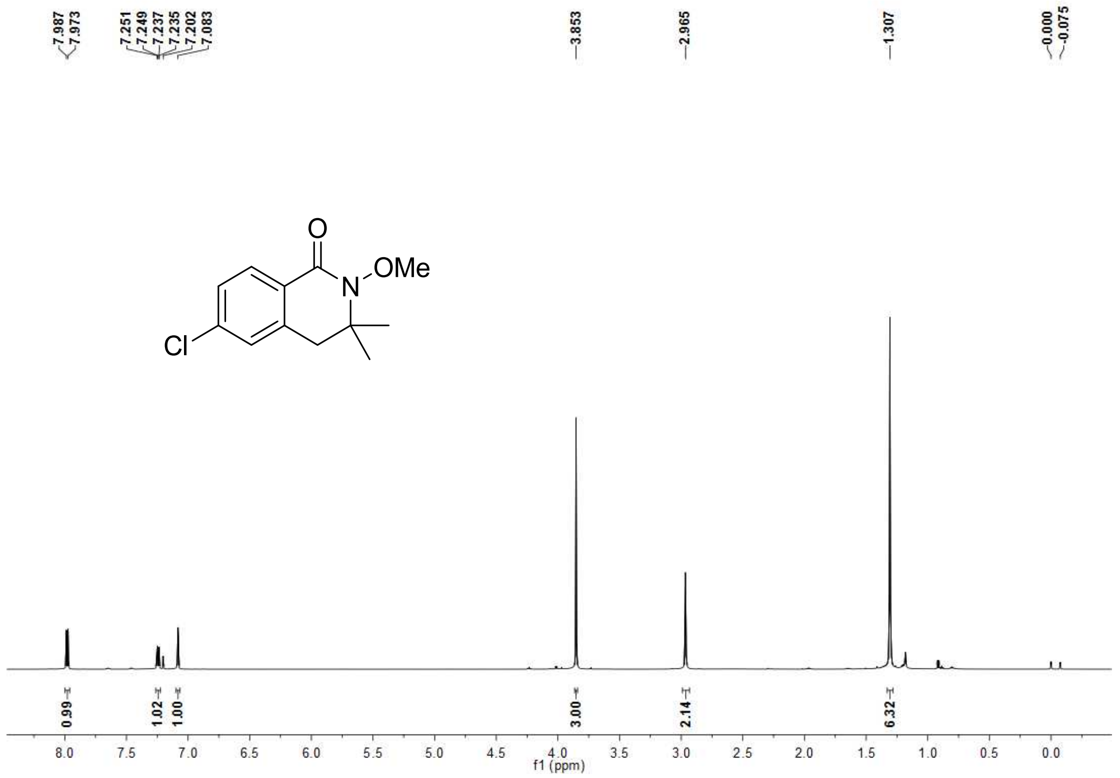
7.987
7.973
7.251
7.249
7.237
7.235
7.202
7.083
3.853
2.965
1.307
0.000
-0.075
O
N
OMe
Cl
0.99
1.02
1.00
3.00
2.14
6.32
f1 (ppm)

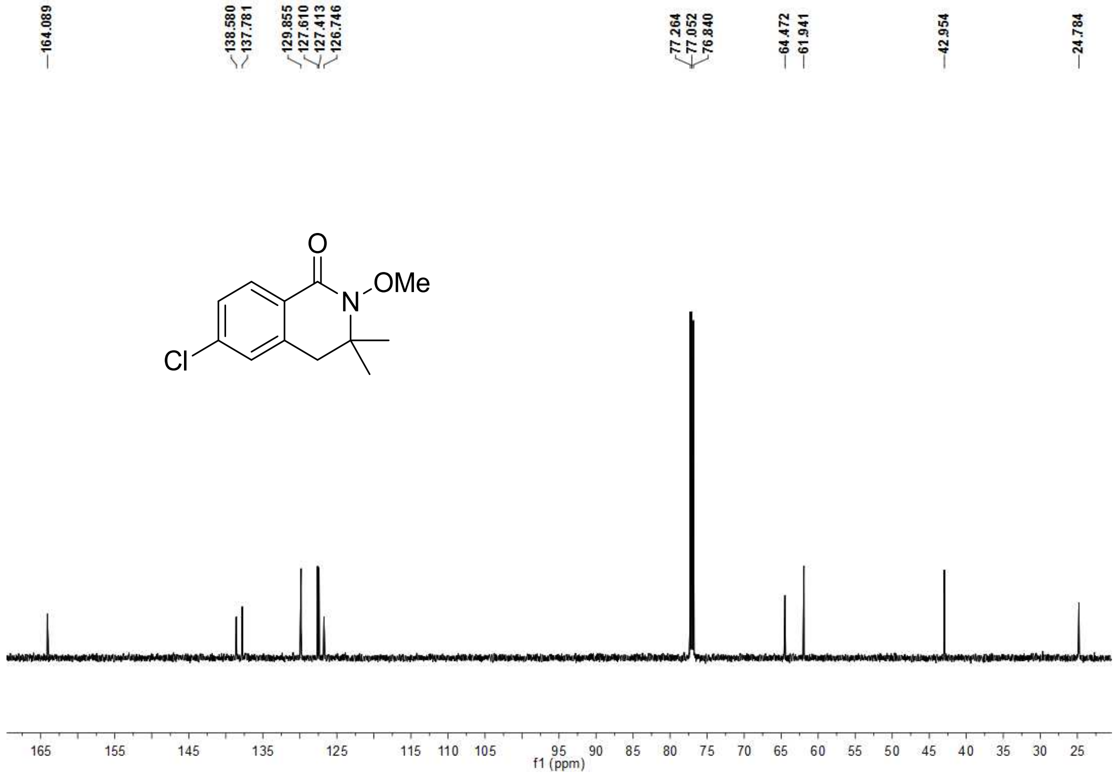
164.089
138.580
137.781
129.855
127.610
127.413
126.746
77.264
77.052
76.840
64.472
61.941
42.954
24.784
O
N
OMe
Cl
f1 (ppm)

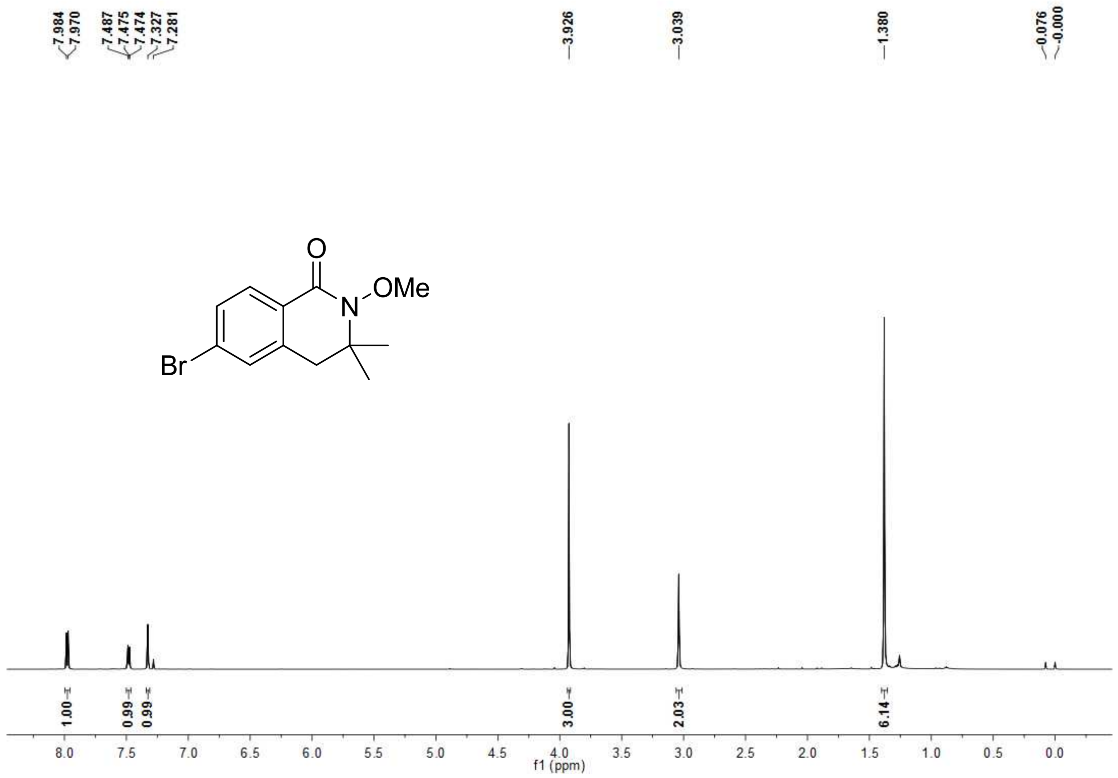
7.984
7.970
7.487
7.475
7.474
7.327
7.281
3.926
3.039
1.380
0.076
-0.000
O
N
OMe
Br
1.00
0.99
0.99
3.00
2.03
6.14
8.0
7.5
7.0
6.5
6.0
5.5
5.0
4.5
4.0
3.5
3.0
2.5
2.0
1.5
1.0
0.5
0.0
f1 (ppm)

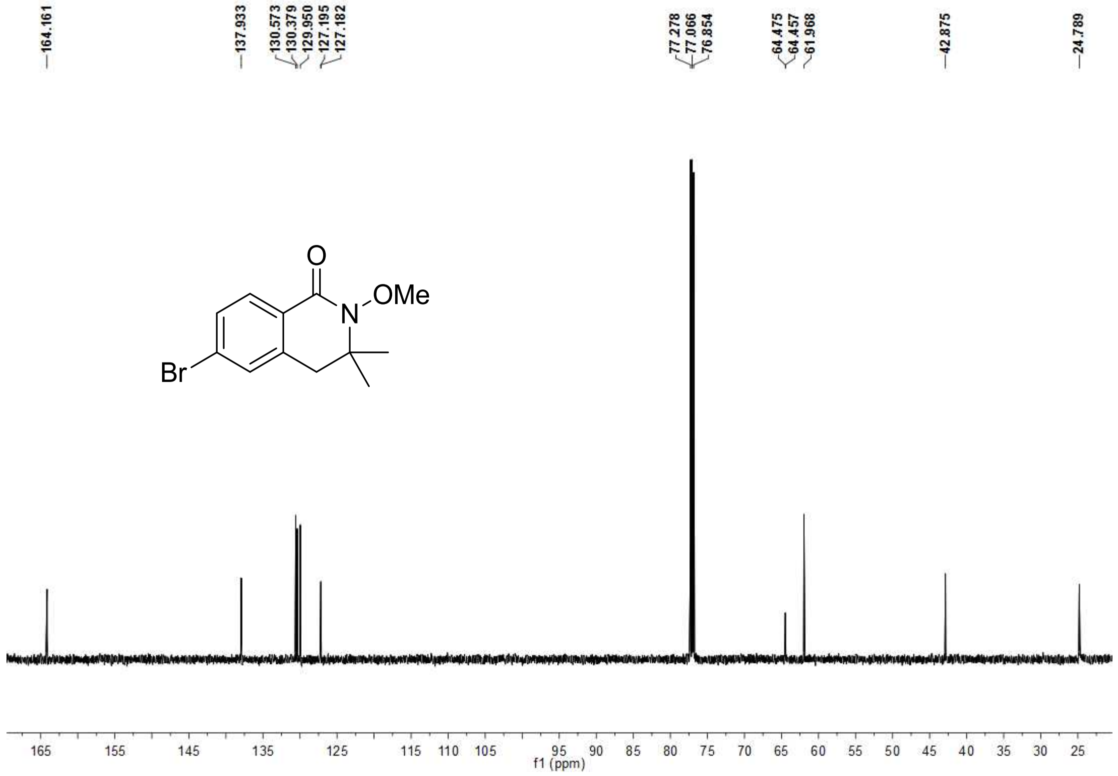
164.161
137.933
130.573
130.379
129.950
127.195
127.182
77.278
77.066
76.854
64.475
64.457
61.968
42.875
24.789
O
N
OMe
Br
165
155
145
135
125
115
110
105
95
90
85
80
75
70
65
60
55
50
45
40
35
30
25
f1 (ppm)

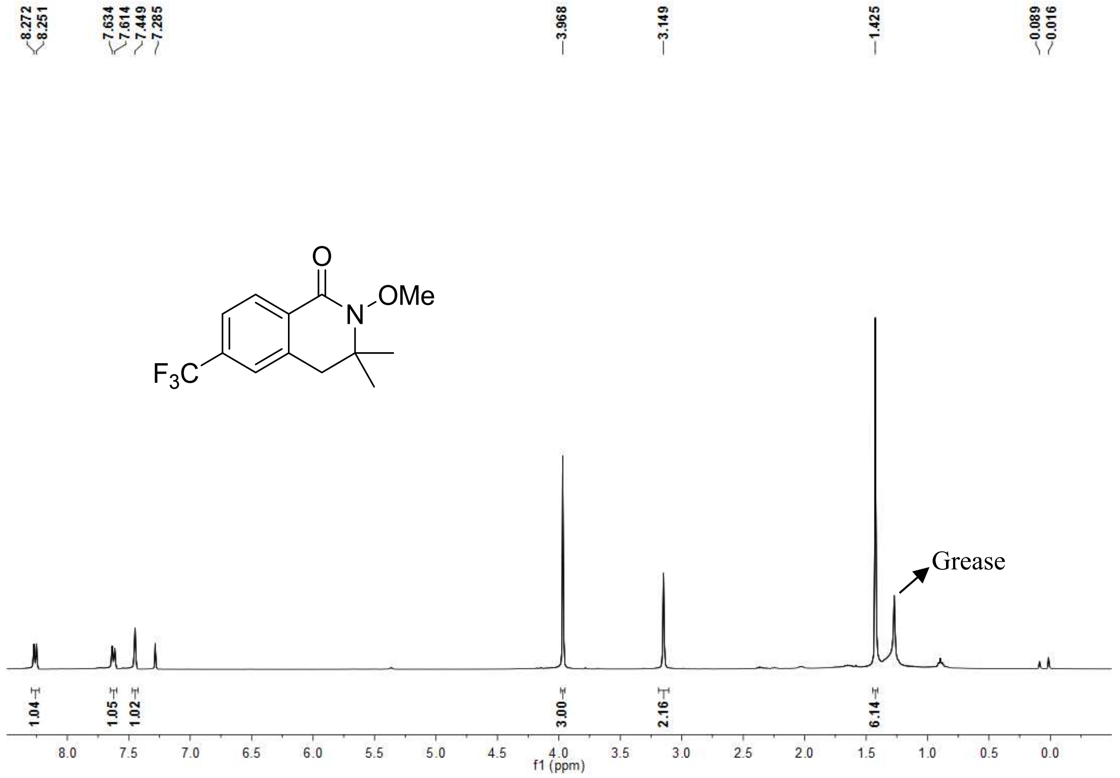
8.272
8.251
7.634
7.614
7.449
7.285
3.968
3.149
1.425
0.089
0.016
O
N
OMe
F3C
Grease
1.04
1.05
1.02
3.00
2.16
6.14
f1 (ppm)

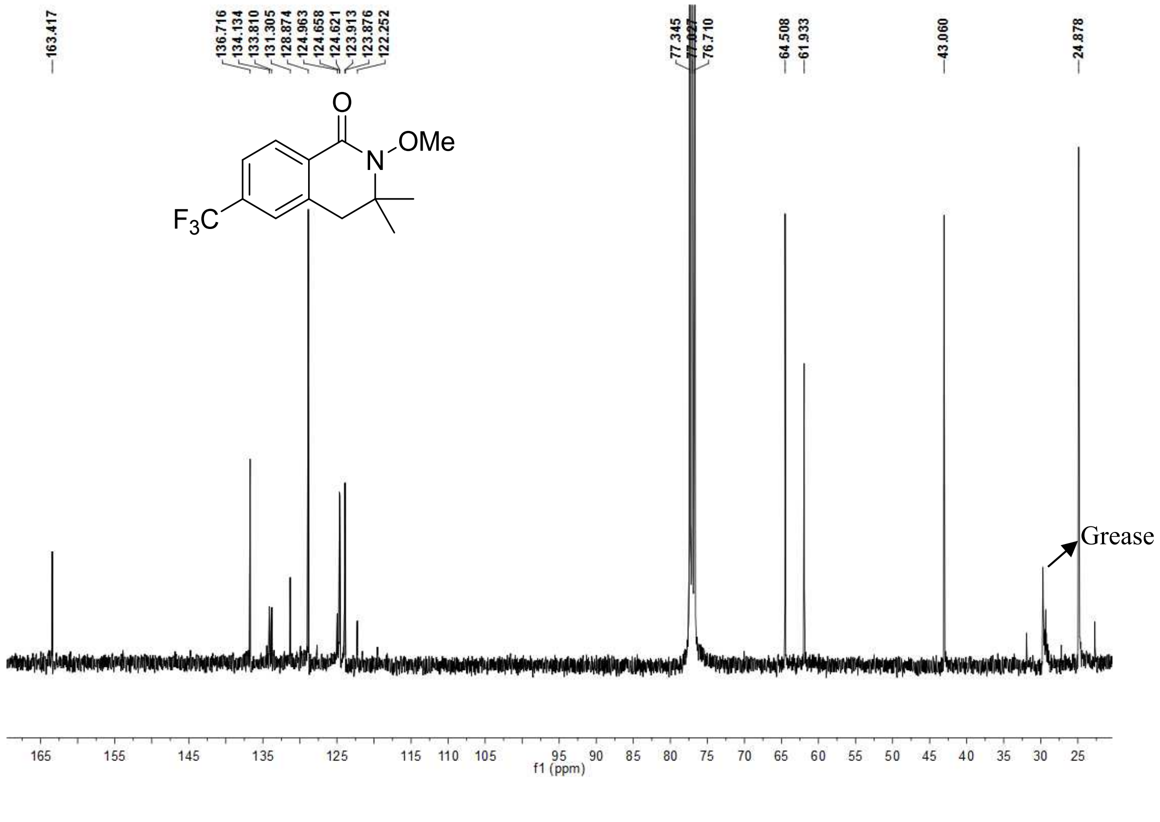
163.417
136.716
134.134
133.810
131.305
128.874
124.963
124.658
124.621
123.913
123.876
122.252
77.345
77.027
76.710
64.508
61.933
43.060
24.878
O
N
OMe
F3C
Grease
f1 (ppm)

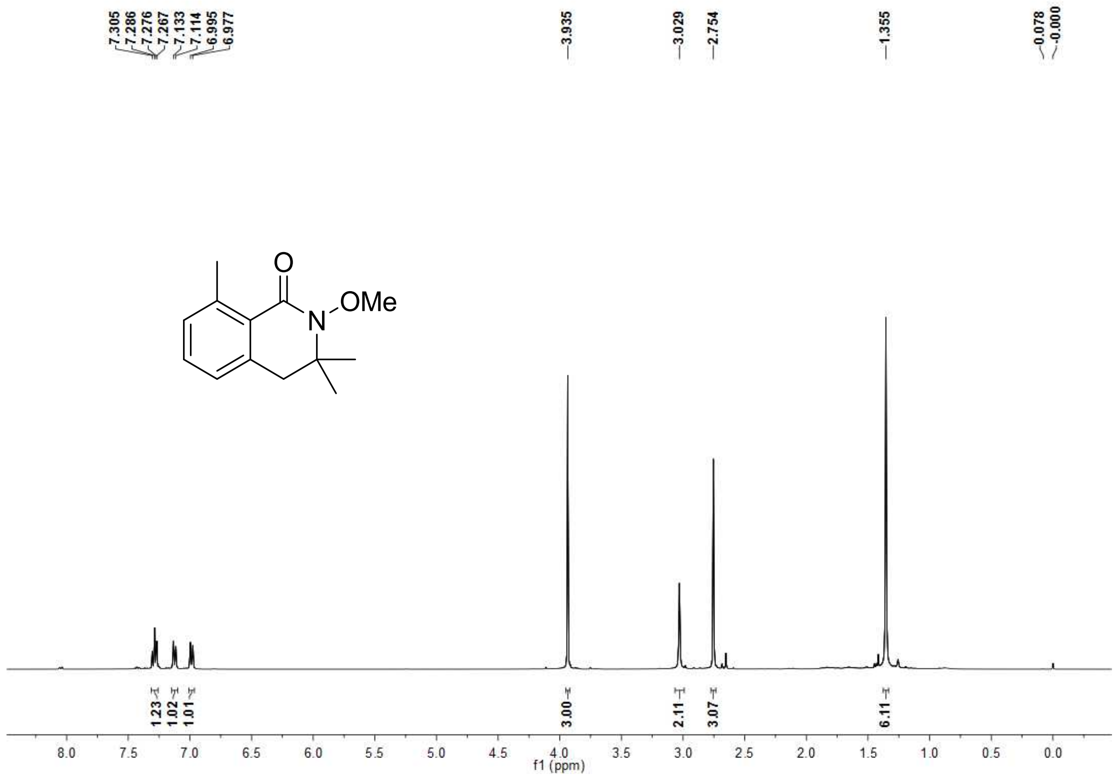
7.305
7.286
7.276
7.267
7.133
7.114
6.995
6.977
3.935
3.029
2.754
1.355
0.078
-0.000
OMe
O
N
1.23
1.02
1.01
3.00
2.11
3.07
6.11
f1 (ppm)

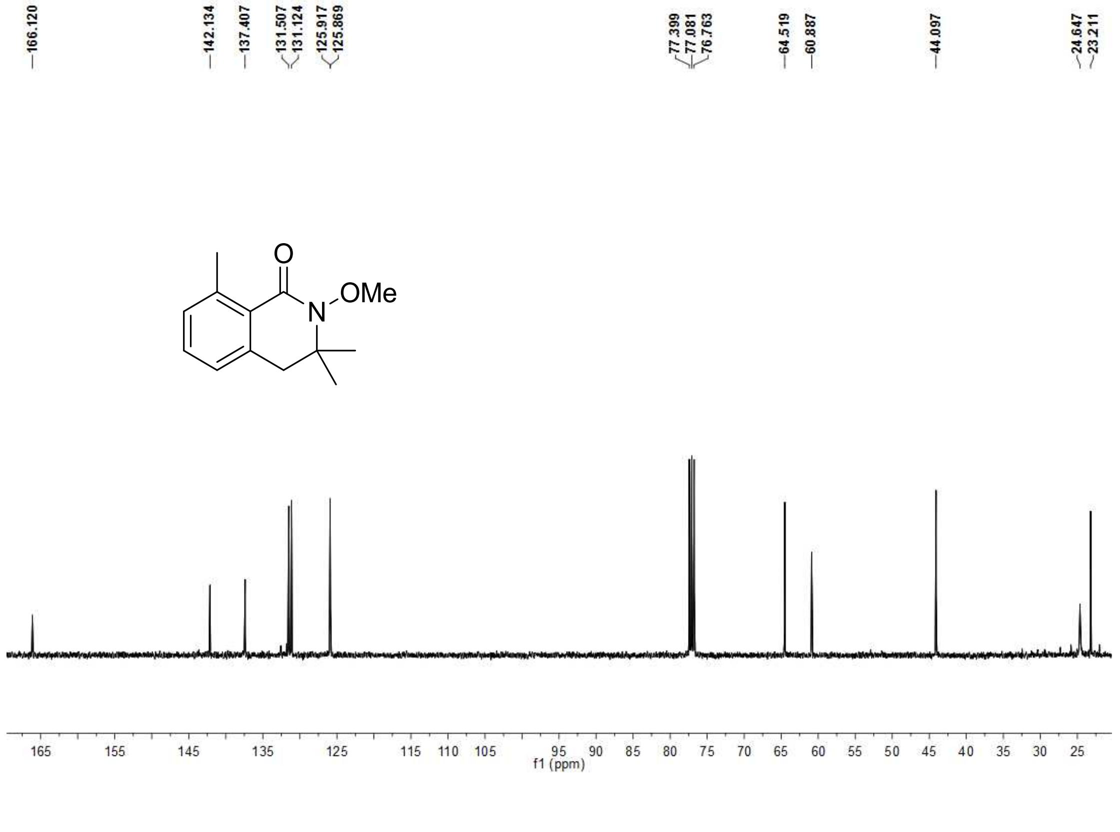
166.120
142.134
137.407
131.507
131.124
125.917
125.869
77.399
77.081
76.763
64.519
60.887
44.097
24.647
23.211
O
N
OMe
f1 (ppm)

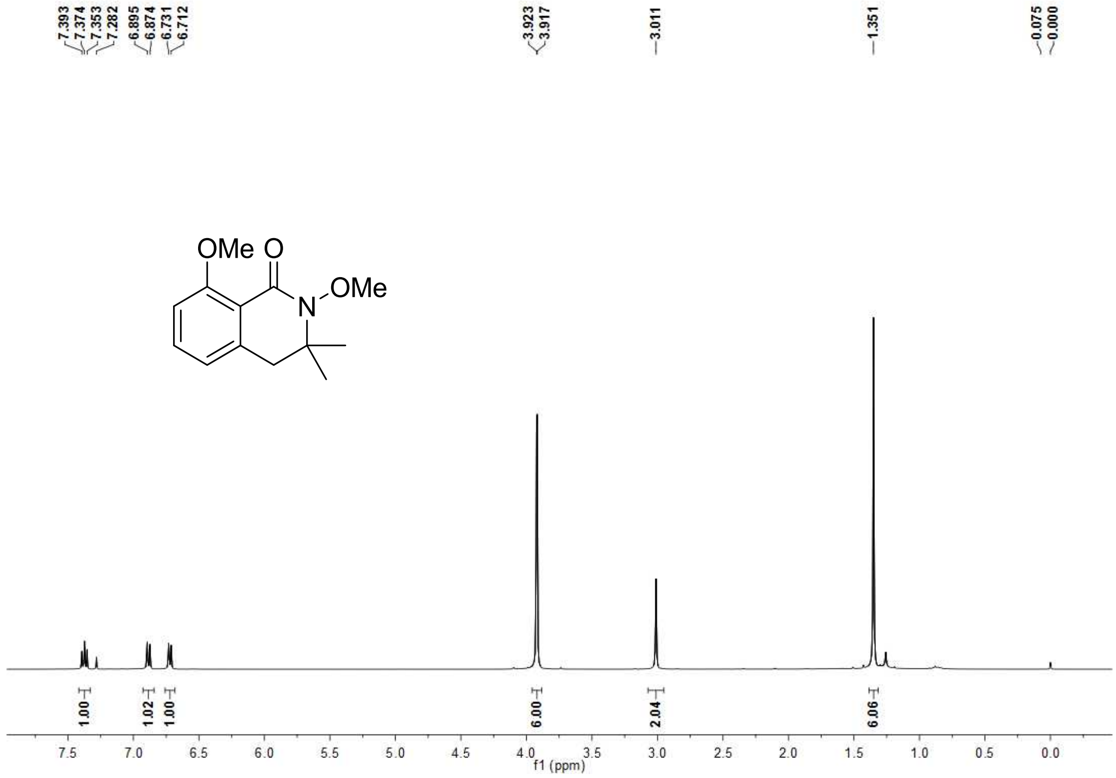
7.393
7.374
7.353
7.282
6.895
6.874
6.731
6.712
3.923
3.917
3.011
1.351
0.075
0.000
OMe O
N
OMe
1.00
1.02
1.00
6.00
2.04
6.06
7.5 7.0 6.5 6.0 5.5 5.0 4.5 4.0 3.5 3.0 2.5 2.0 1.5 1.0 0.5 0.0
f1 (ppm)

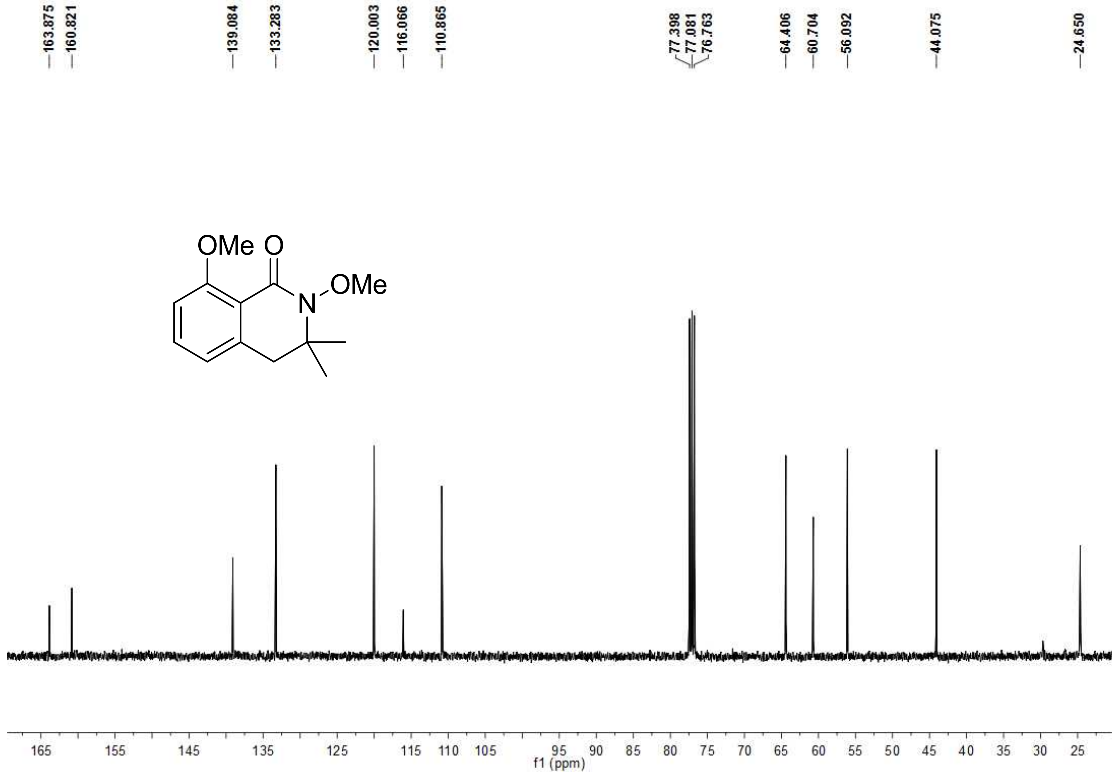
163.875
160.821
139.084
133.283
120.003
116.066
110.865
77.398
77.081
76.763
64.406
60.704
56.092
44.075
24.650
OMe O
N
OMe
165 155 145 135 125 115 110 105 95 90 85 80 75 70 65 60 55 50 45 40 35 30 25
f1 (ppm)

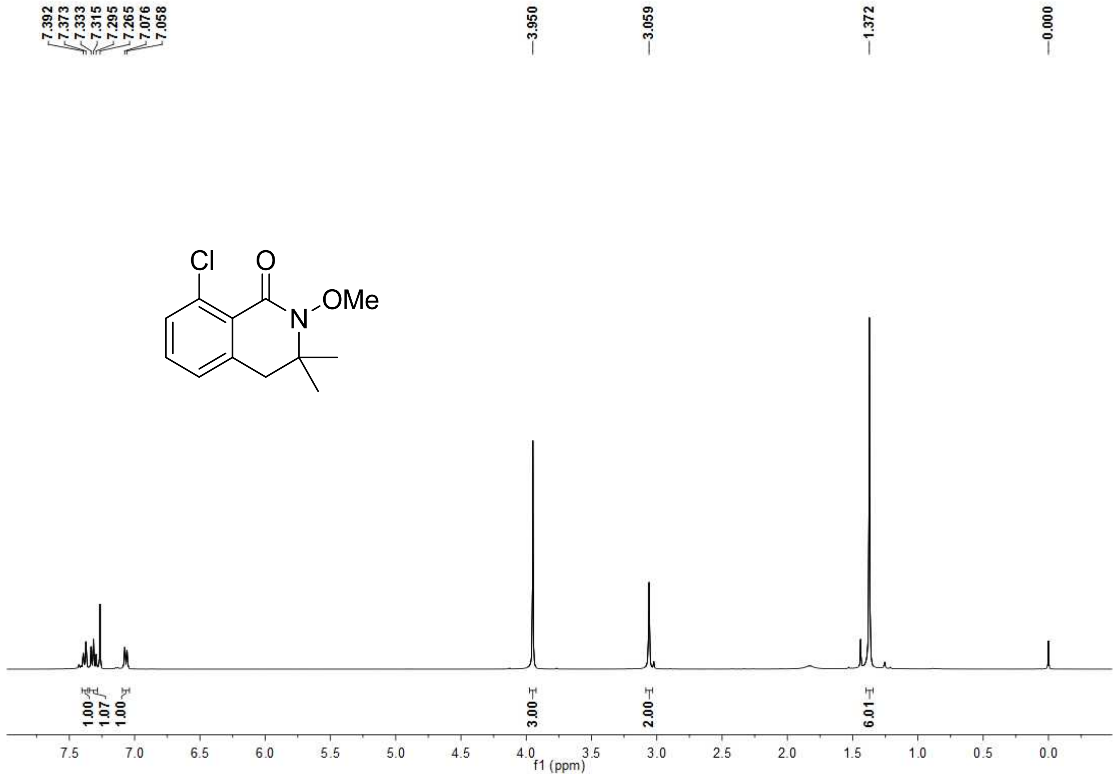
7.392
7.373
7.333
7.315
7.295
7.265
7.076
7.058
3.950
3.059
1.372
0.000
Cl
O
N
OMe
1.00
1.07
1.00
3.00
2.00
6.01
f1 (ppm)

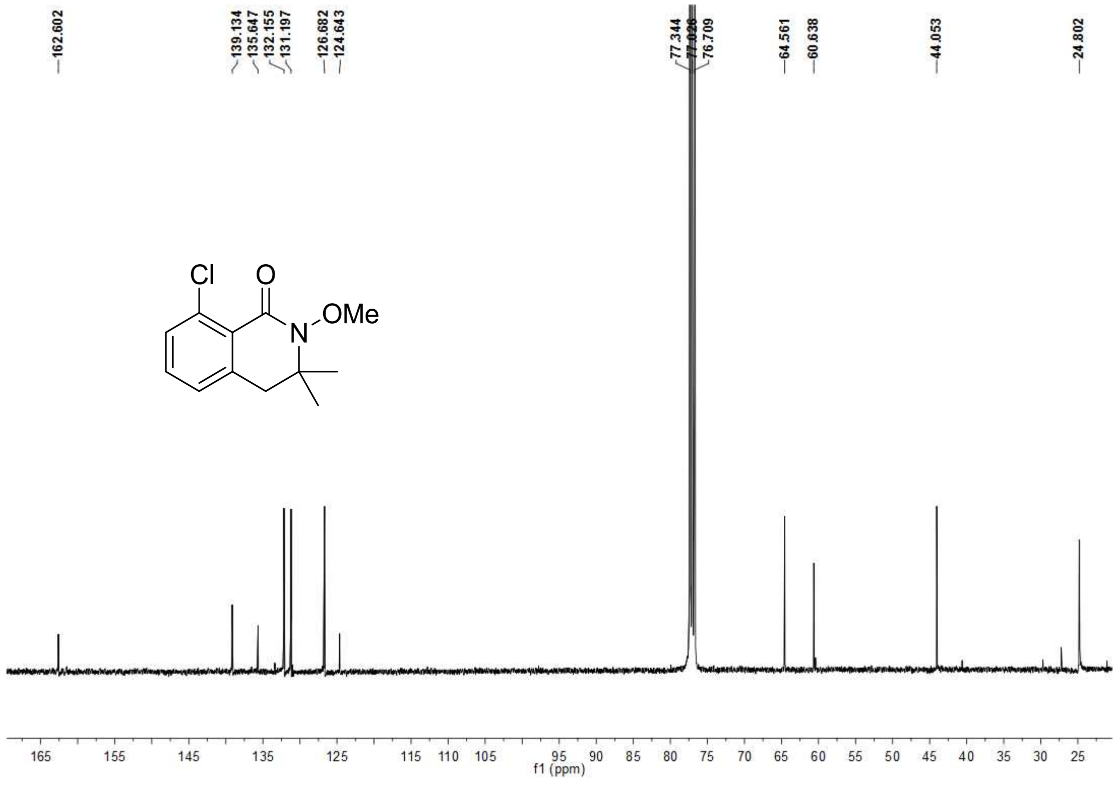
162.602
139.134
135.647
132.155
131.197
126.682
124.643
77.344
77.026
76.709
64.561
60.638
44.053
24.802
Cl
O
N
OMe
f1 (ppm)

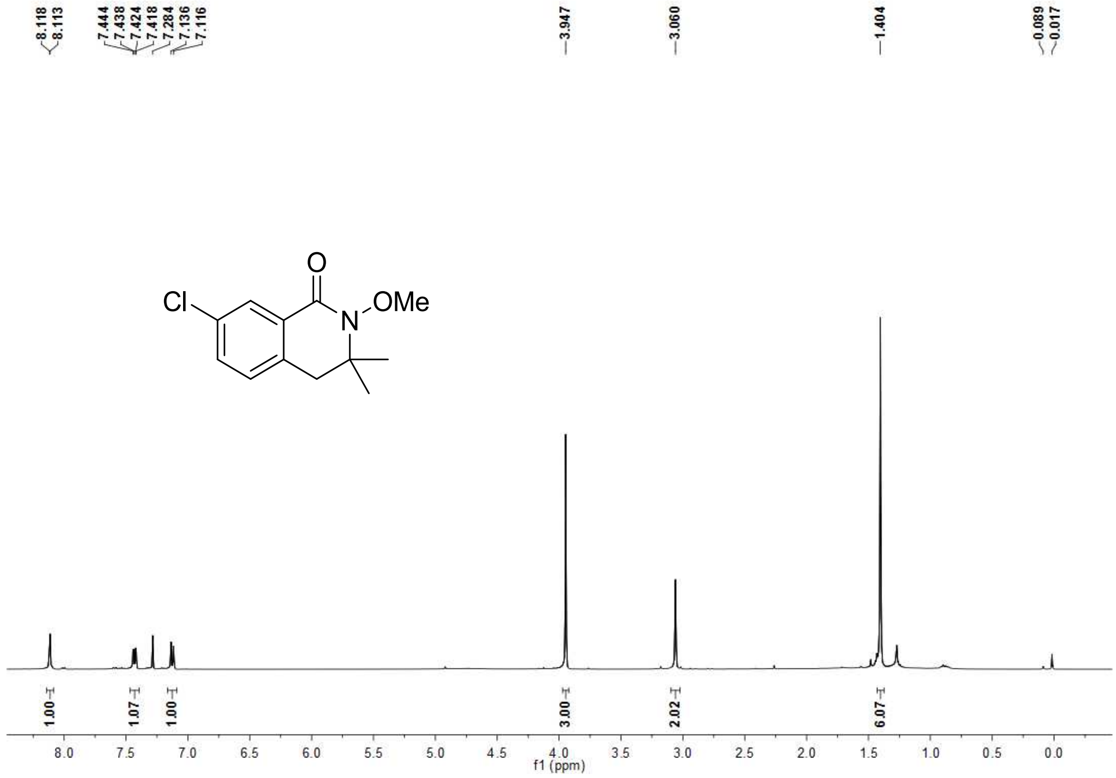
8.118
8.113
7.444
7.438
7.424
7.418
7.284
7.136
7.116
3.947
3.060
1.404
0.089
0.017
Cl
O
N
OMe
1.00
1.07
1.00
3.00
2.02
6.07
8.0 7.5 7.0 6.5 6.0 5.5 5.0 4.5 4.0 3.5 3.0 2.5 2.0 1.5 1.0 0.5 0.0
f1 (ppm)

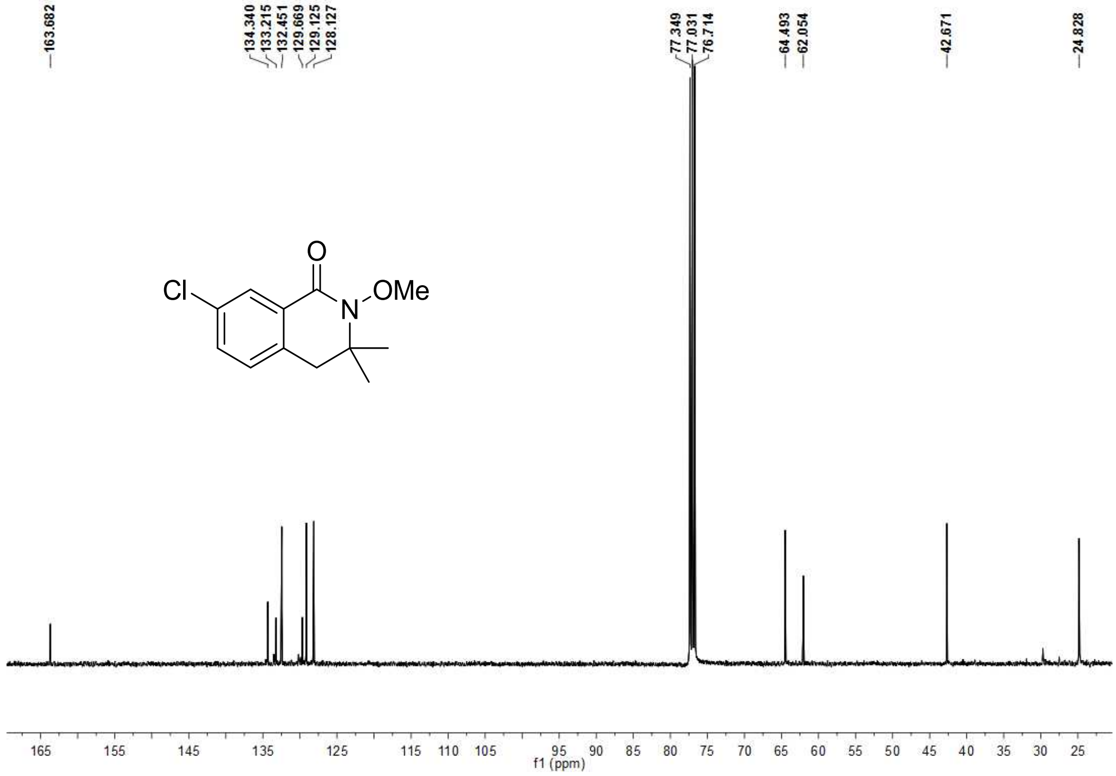
163.682
134.340
133.215
132.451
129.669
129.125
128.127
77.349
77.031
76.714
64.493
62.054
42.671
24.828
Cl
O
N
OMe
165 155 145 135 125 115 110 105 95 90 85 80 75 70 65 60 55 50 45 40 35 30 25
f1 (ppm)

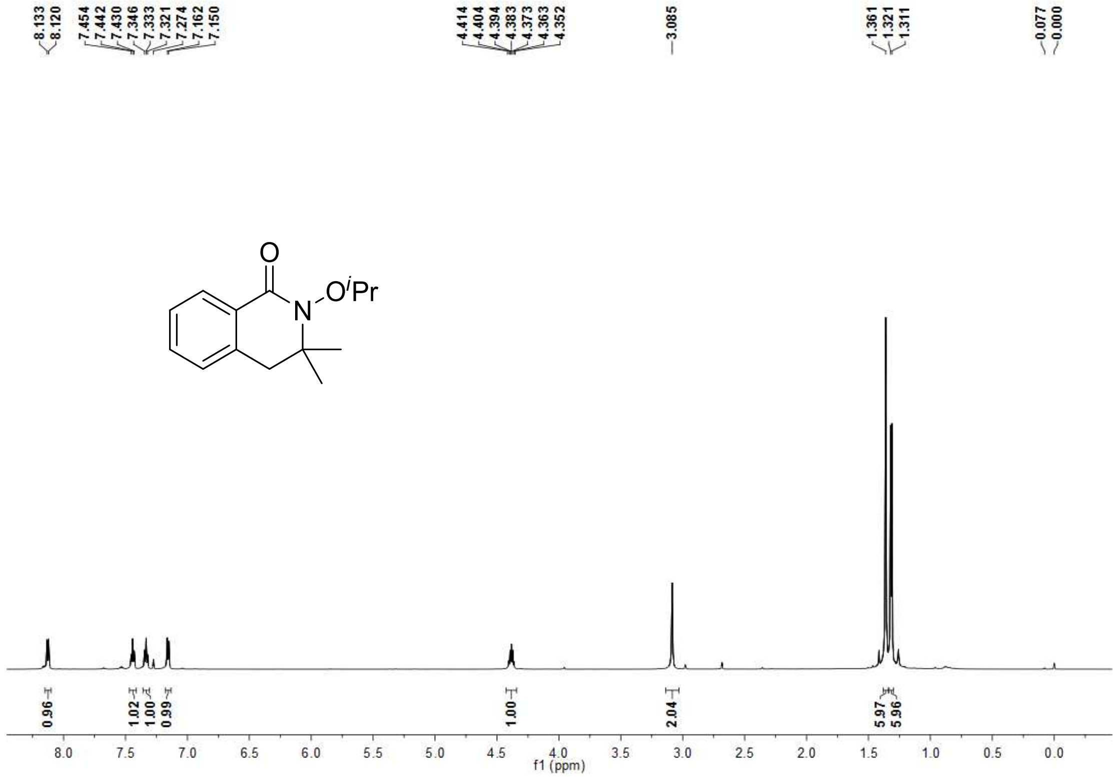
8.133
8.120
7.454
7.442
7.430
7.346
7.333
7.321
7.274
7.162
7.150
4.414
4.404
4.394
4.383
4.373
4.363
4.352
3.085
1.361
1.321
1.311
0.077
0.000
O
N
O^{i}Pr
0.96
1.02
1.00
0.99
1.00
2.04
5.97
5.96
f1 (ppm)

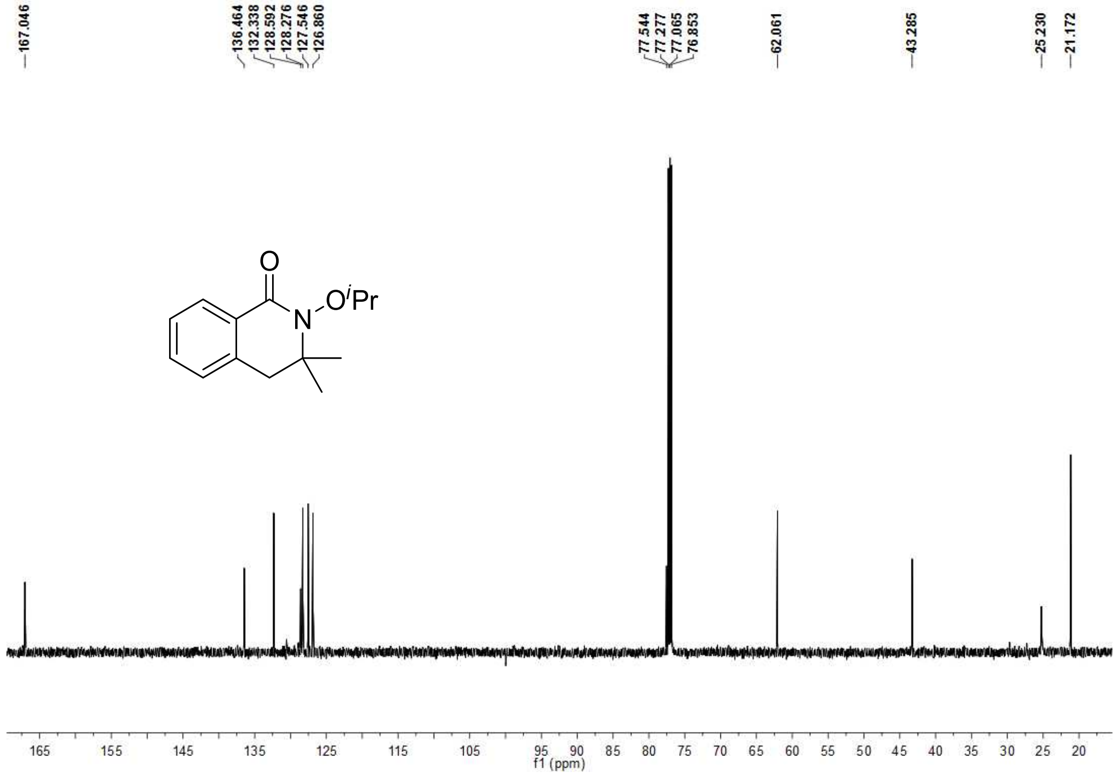
167.046
136.464
132.338
128.592
128.276
127.546
126.860
77.544
77.277
77.065
76.853
62.061
43.285
25.230
21.172
O
N
O^{i}Pr
f1 (ppm)

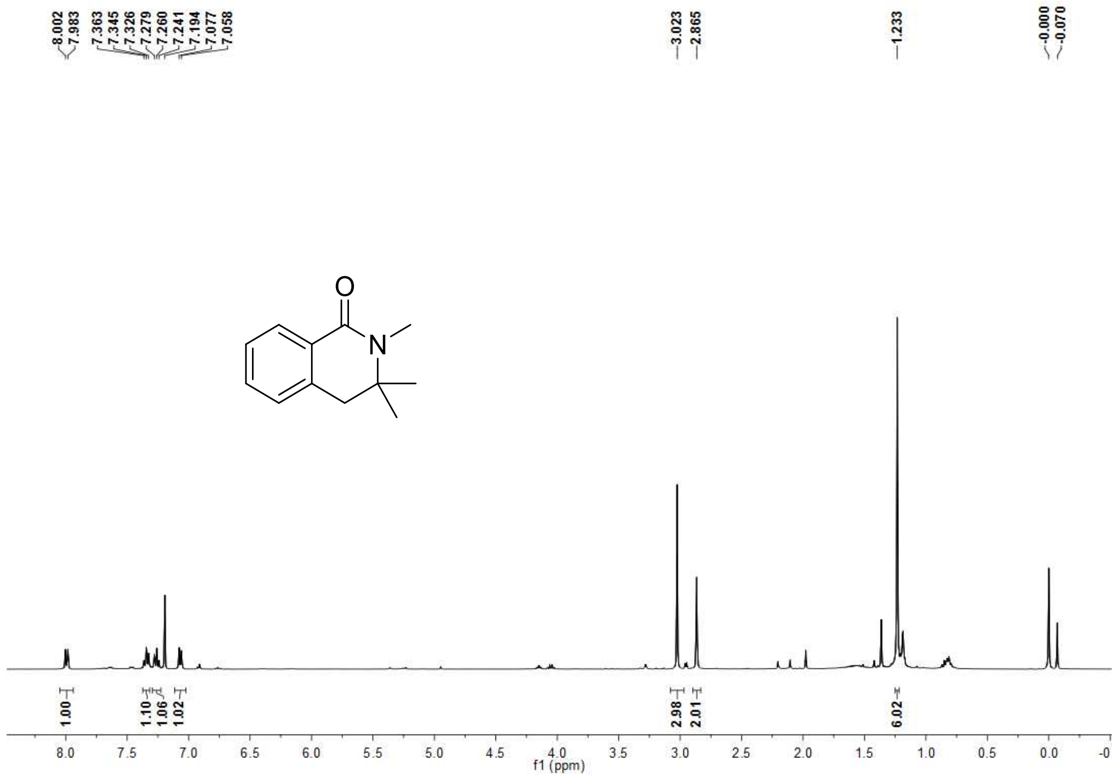
8.002
7.983
7.363
7.345
7.326
7.279
7.260
7.241
7.194
7.077
7.058
3.023
2.865
1.233
-0.000
-0.070
O
N
1.00
1.10
1.06
1.02
2.98
2.01
6.02
f1 (ppm)

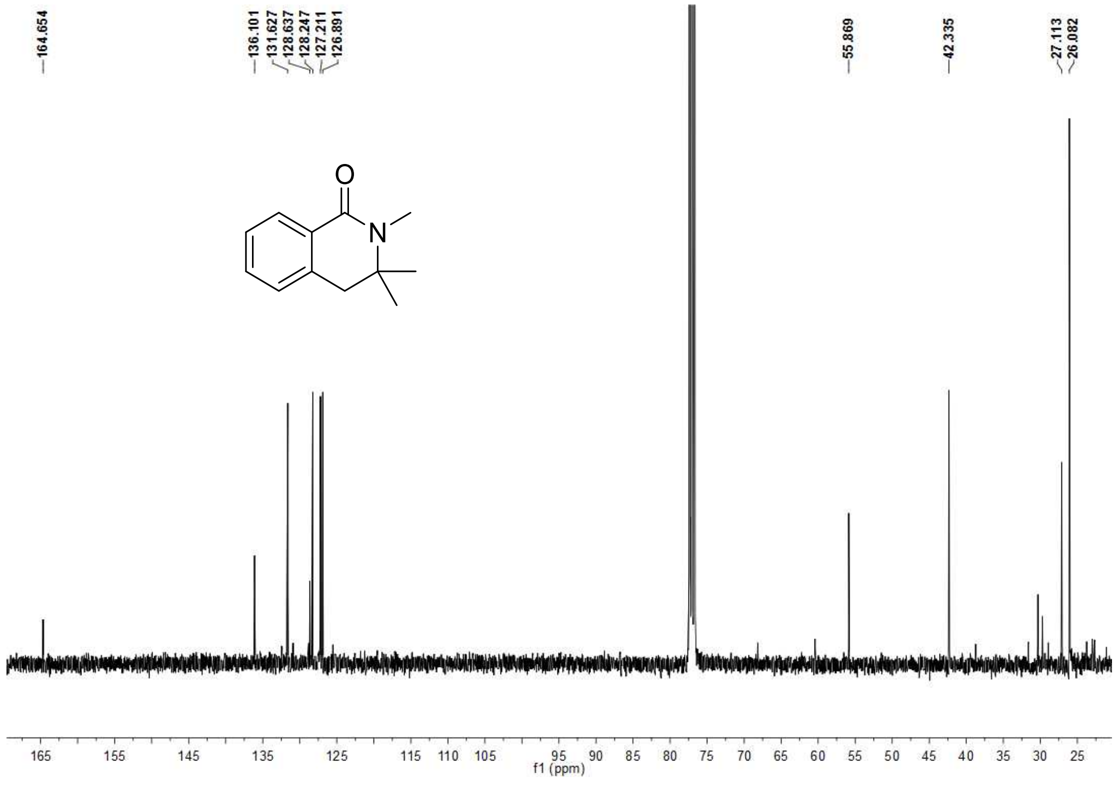
164.654
136.101
131.627
128.637
128.247
127.211
126.891
55.869
42.335
27.113
26.082
O
N
f1 (ppm)

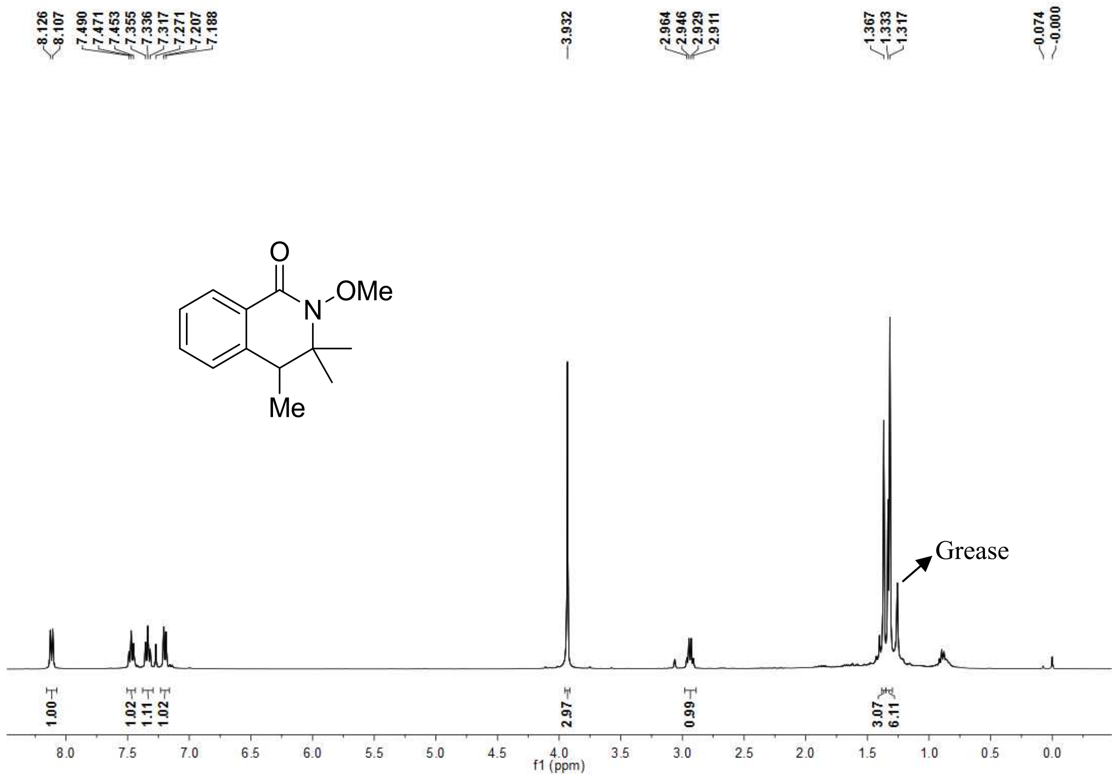
8.126
8.107
7.490
7.471
7.453
7.355
7.336
7.317
7.271
7.207
7.188
3.932
2.964
2.946
2.929
2.911
1.367
1.333
1.317
0.074
-0.000
O
N
OMe
Me
Grease
1.00
1.02
1.11
1.02
2.97
0.99
3.07
6.11
f1 (ppm)

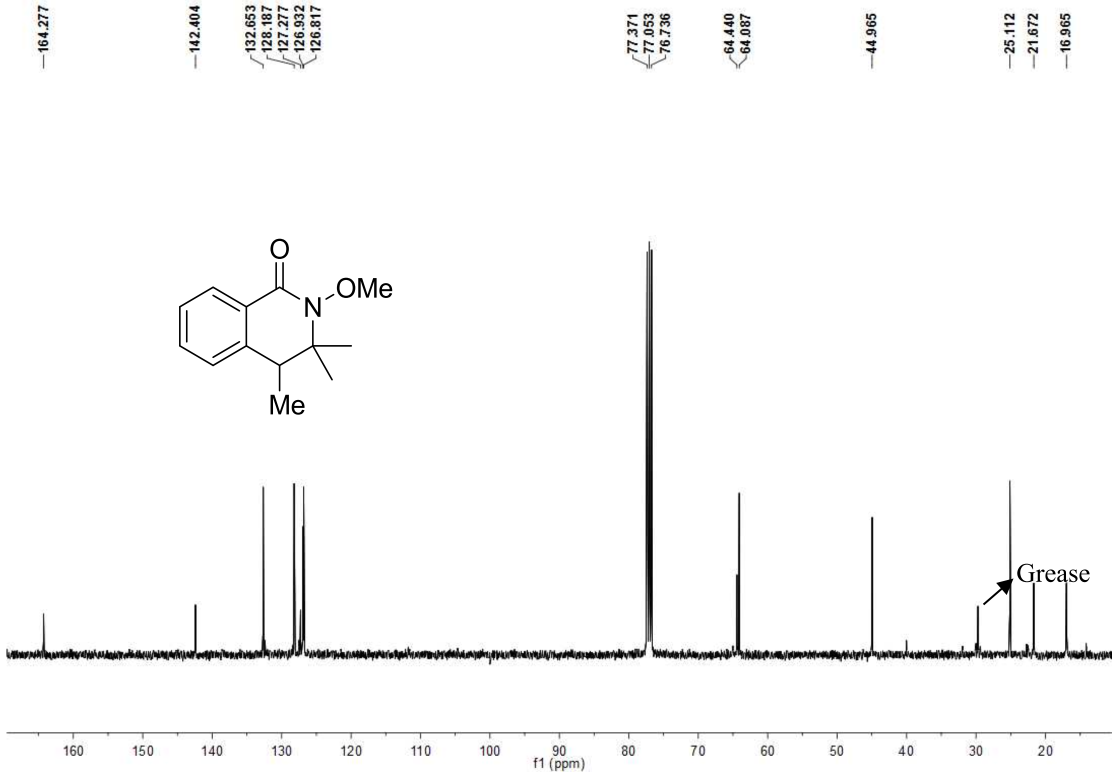
164.277
142.404
132.653
128.187
127.277
126.932
126.817
77.371
77.053
76.736
64.440
64.087
44.965
25.112
21.672
16.965
O
N
OMe
Me
Grease
f1 (ppm)

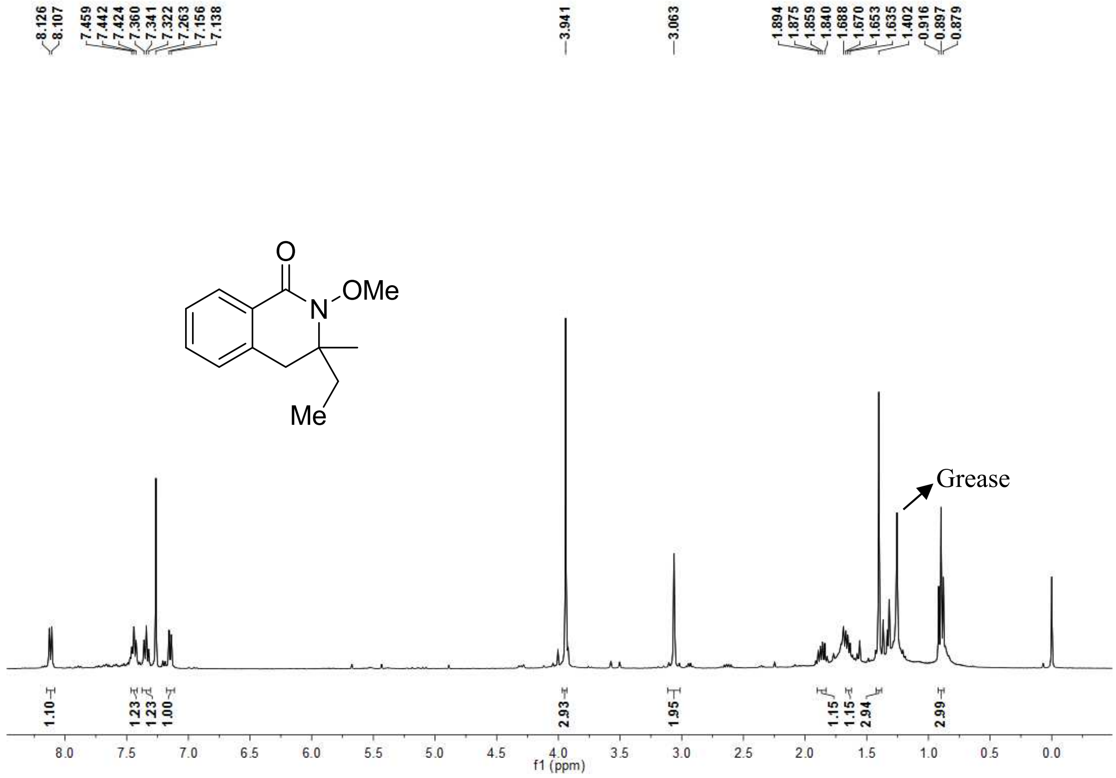
8.126
8.107
7.459
7.442
7.424
7.360
7.341
7.322
7.263
7.156
7.138
3.941
3.063
1.894
1.875
1.859
1.840
1.688
1.670
1.653
1.635
1.402
0.916
0.897
0.879
O
N
OMe
Me
Grease
1.10
1.23
1.23
1.00
2.93
1.95
1.15
1.15
2.94
2.99
f1 (ppm)

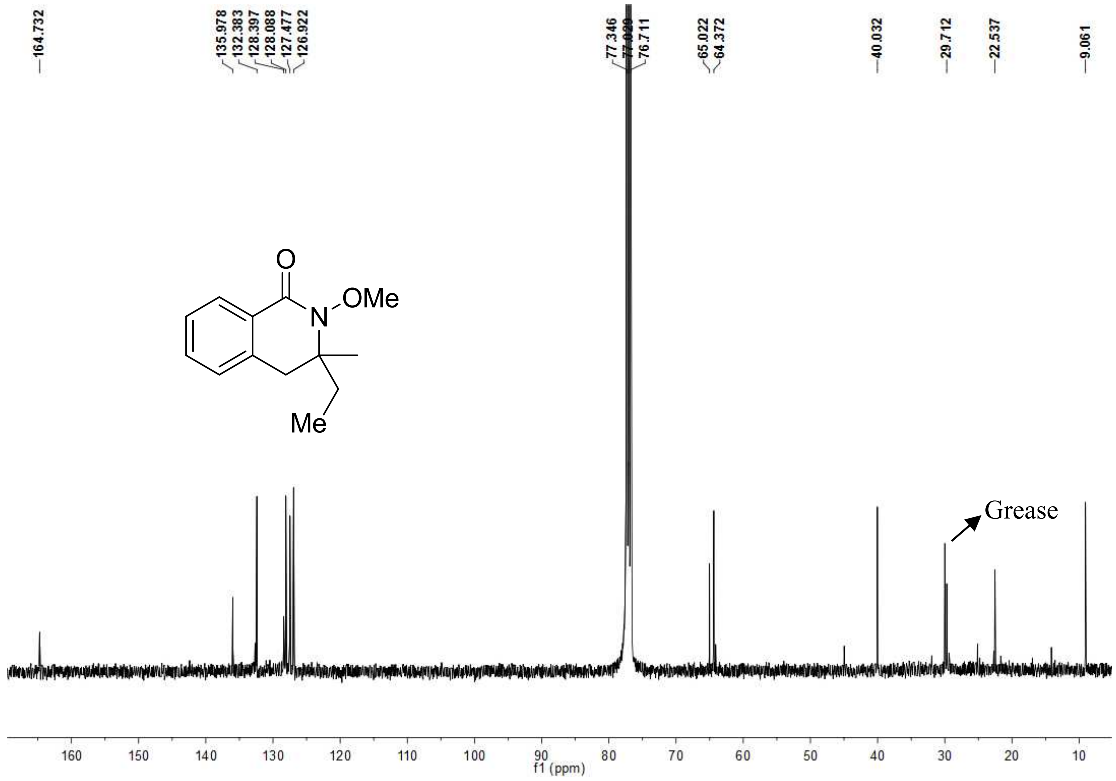
164.732
135.978
132.383
128.397
128.088
127.477
126.922
77.346
76.711
65.022
64.372
40.032
29.712
22.537
9.061
O
N
OMe
Me
Grease
f1 (ppm)

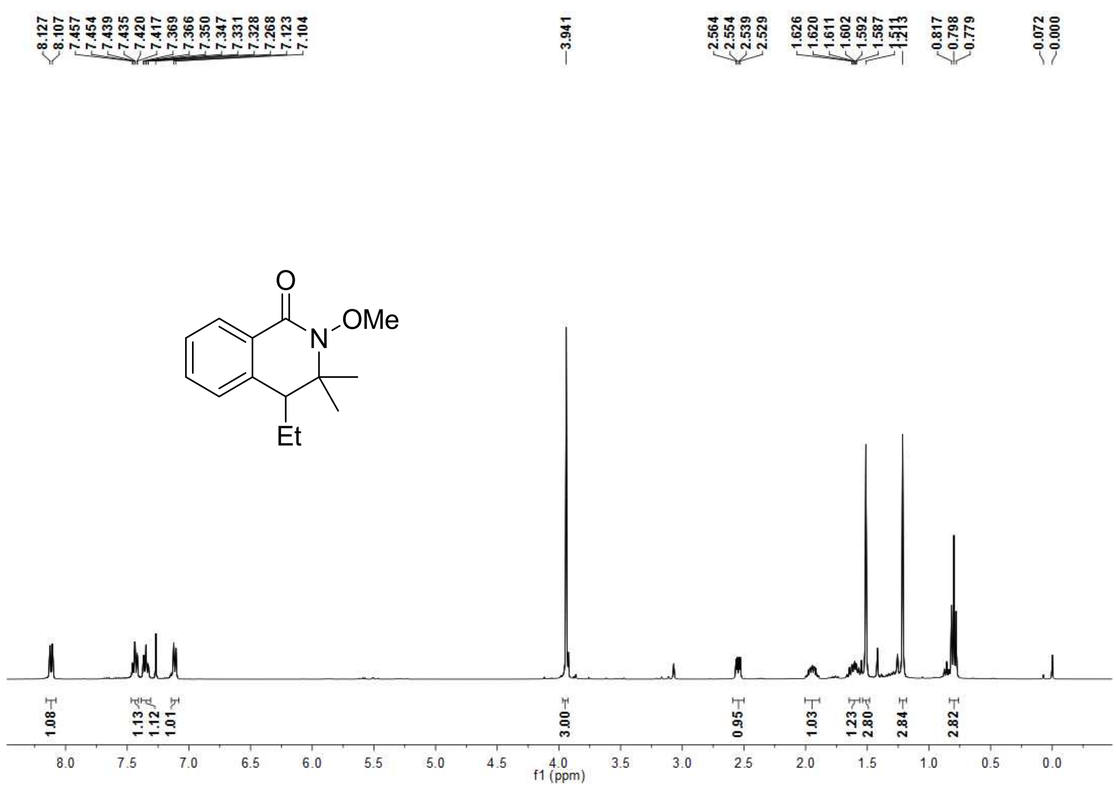
8.127
8.107
7.457
7.454
7.439
7.435
7.420
7.417
7.369
7.366
7.350
7.347
7.331
7.328
7.268
7.123
7.104
3.941
2.564
2.554
2.539
2.529
1.626
1.620
1.611
1.602
1.592
1.587
1.511
1.213
0.817
0.798
0.779
0.072
0.000
O
N
OMe
Et
1.08
1.13
1.12
1.01
3.00
0.95
1.03
1.23
2.80
2.84
2.82
8.0
7.5
7.0
6.5
6.0
5.5
5.0
4.5
4.0
3.5
3.0
2.5
2.0
1.5
1.0
0.5
0.0
f1 (ppm)

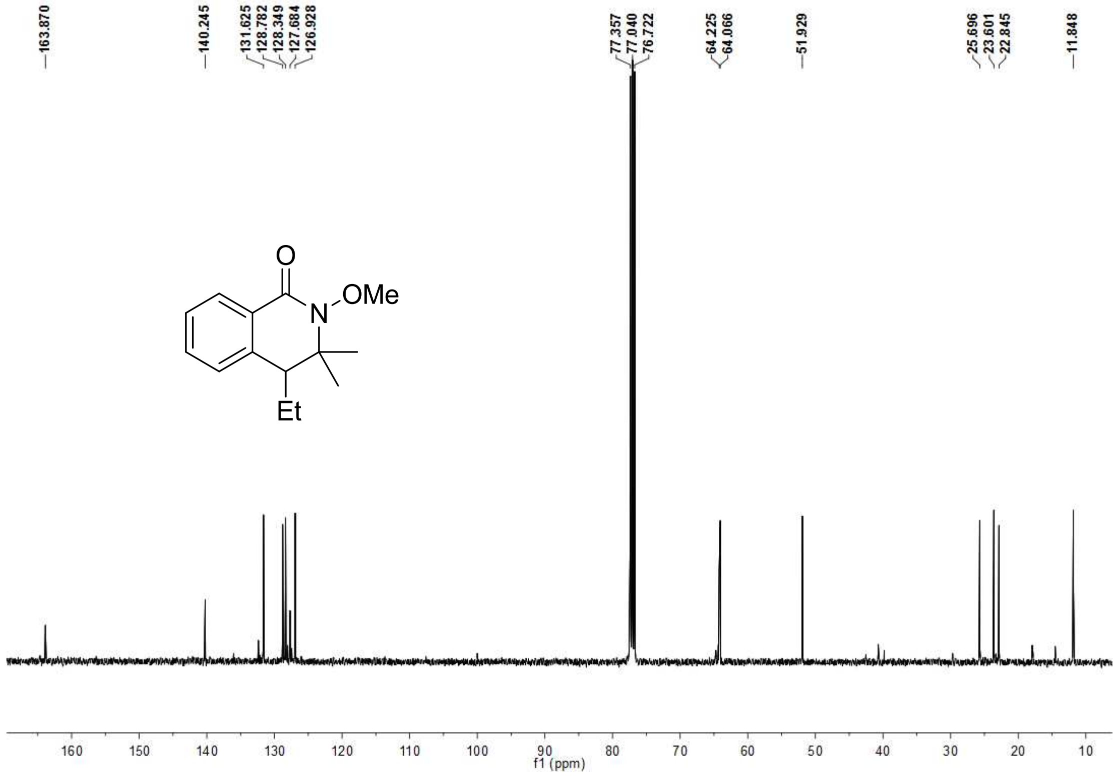
163.870
140.245
131.625
128.782
128.349
127.684
126.928
77.357
77.040
76.722
64.225
64.066
51.929
25.696
23.601
22.845
11.848
O
N
OMe
Et
160
150
140
130
120
110
100
90
80
70
60
50
40
30
20
10
f1 (ppm)

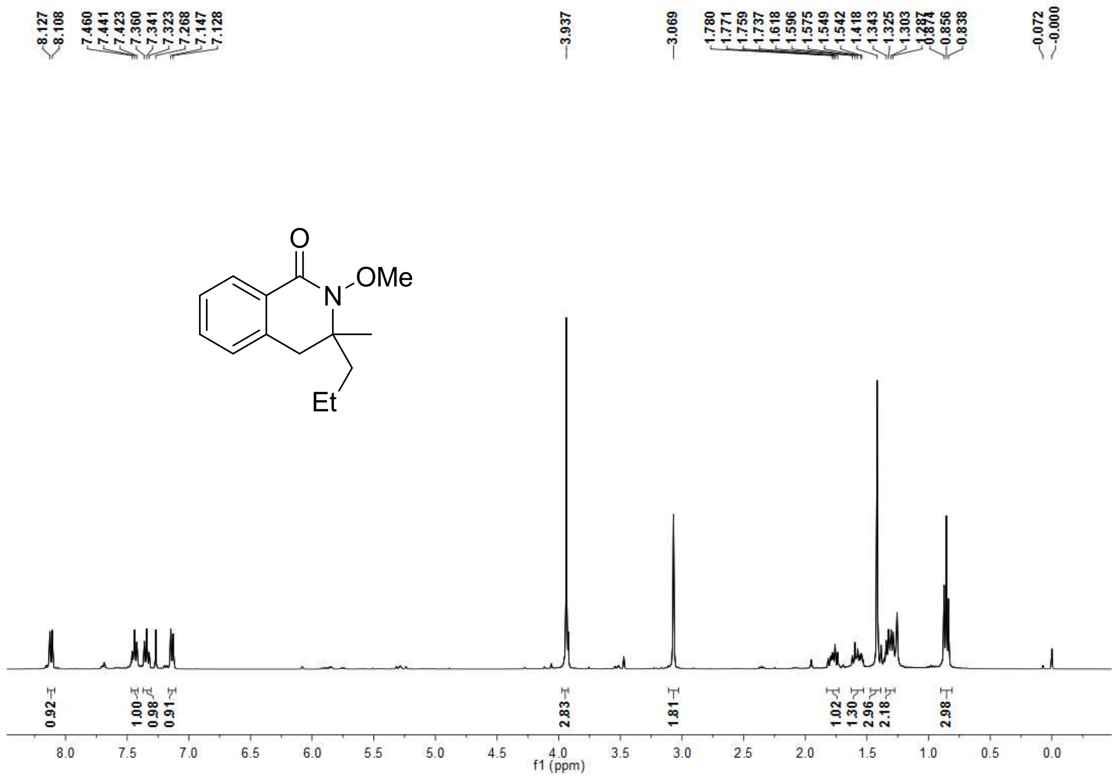

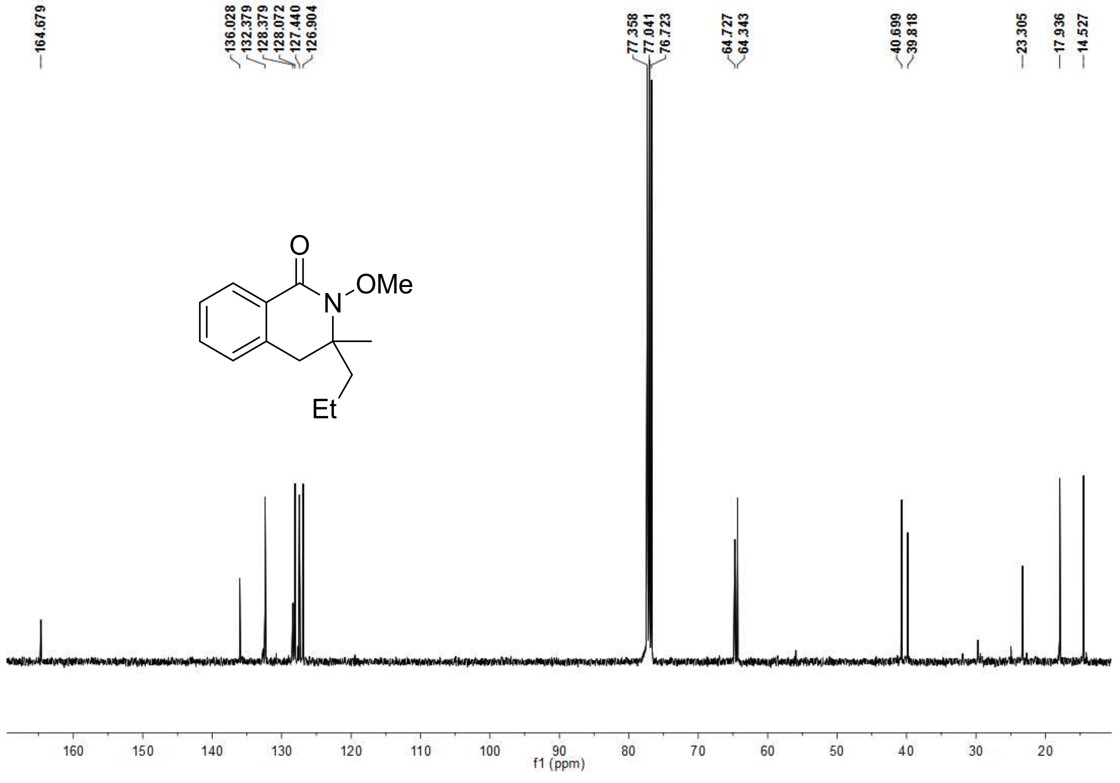

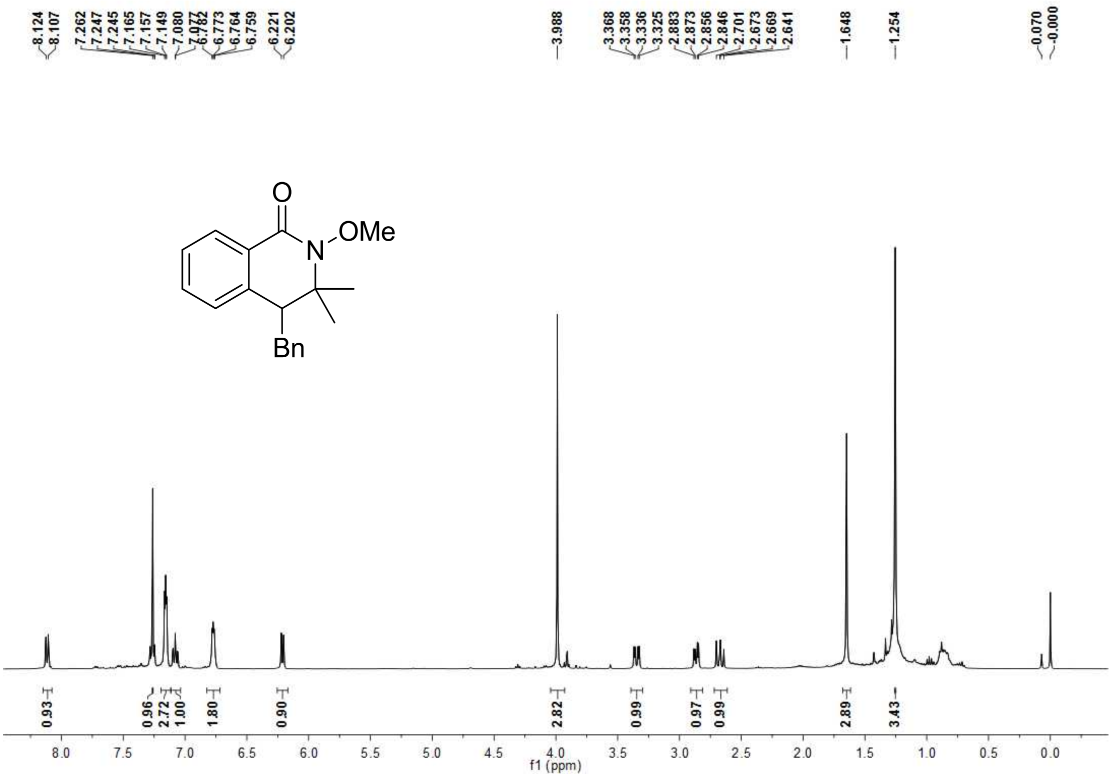
8.124
8.107
7.262
7.247
7.245
7.165
7.157
7.149
7.080
6.782
6.773
6.764
6.759
6.221
6.202
3.988
3.368
3.358
3.336
3.325
2.883
2.873
2.856
2.846
2.701
2.673
2.669
2.641
1.648
1.254
0.070
-0.000
O
N
OMe
Bn
0.93
0.96
2.72
1.00
1.80
0.90
2.82
0.99
0.97
0.99
2.89
3.43
f1 (ppm)

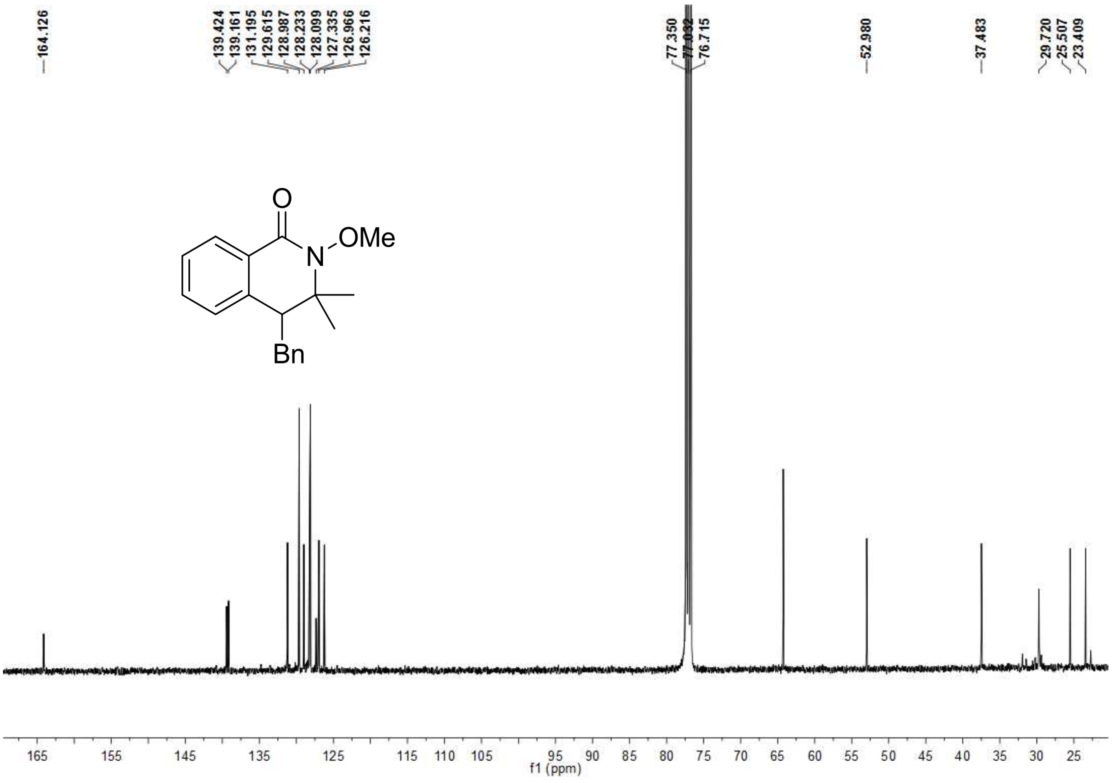
164.126
139.424
139.161
131.195
129.615
128.987
128.233
128.099
127.335
126.966
126.216
77.350
77.032
76.715
52.980
37.483
29.720
25.507
23.409
O
N
OMe
Bn
f1 (ppm)

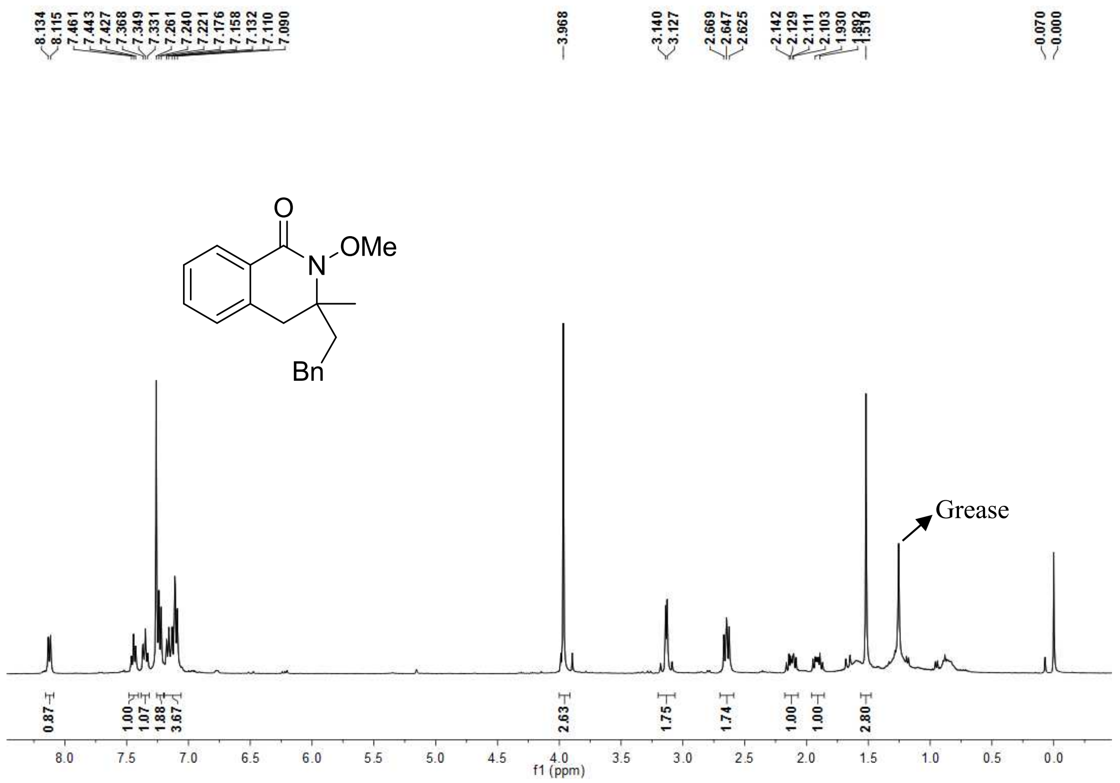
8.134
8.115
7.461
7.443
7.427
7.368
7.349
7.331
7.261
7.240
7.221
7.176
7.158
7.132
7.110
7.090
3.968
3.140
3.127
2.669
2.647
2.625
2.142
2.129
2.111
2.103
1.930
1.519
0.070
0.000
O
N
OMe
Bn
Grease
0.87
1.00
1.07
1.88
3.67
2.63
1.75
1.74
1.00
1.00
2.80
f1 (ppm)

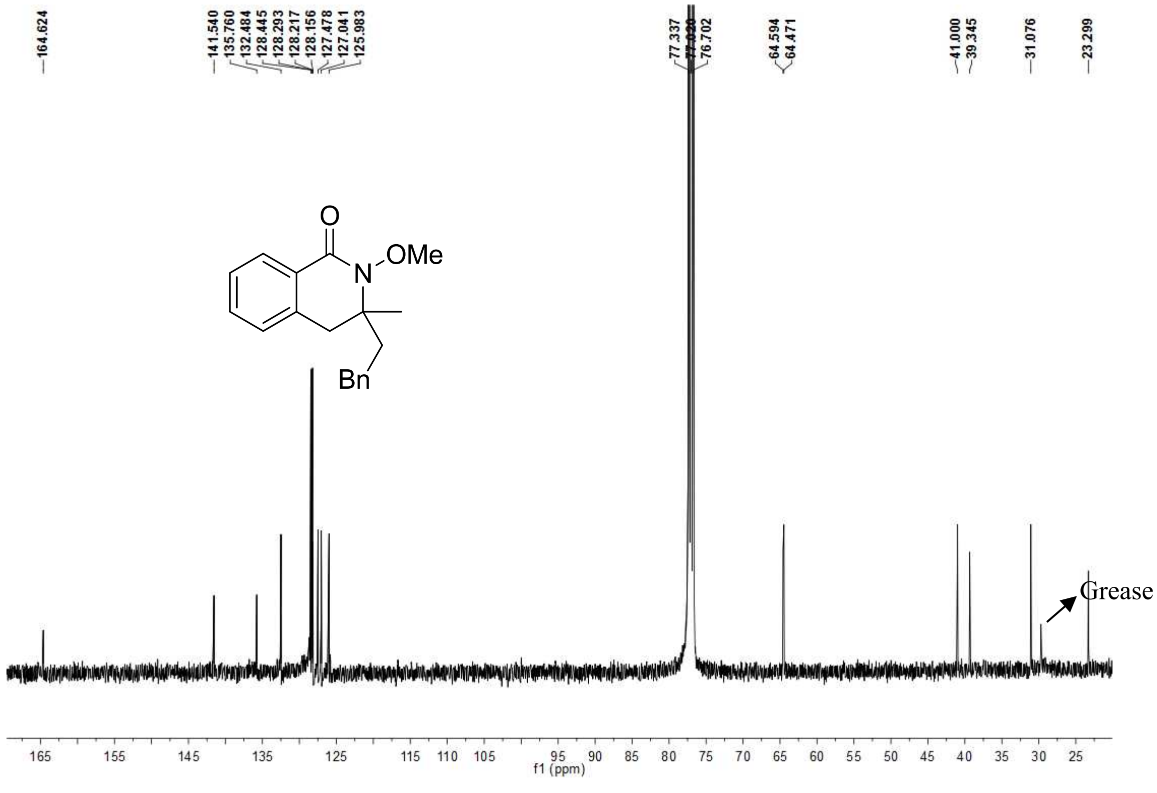
164.624
141.540
135.760
132.484
128.445
128.293
128.217
128.156
127.478
127.041
125.983
77.337
76.702
64.594
64.471
41.000
39.345
31.076
23.299
O
N
OMe
Bn
Grease
f1 (ppm)

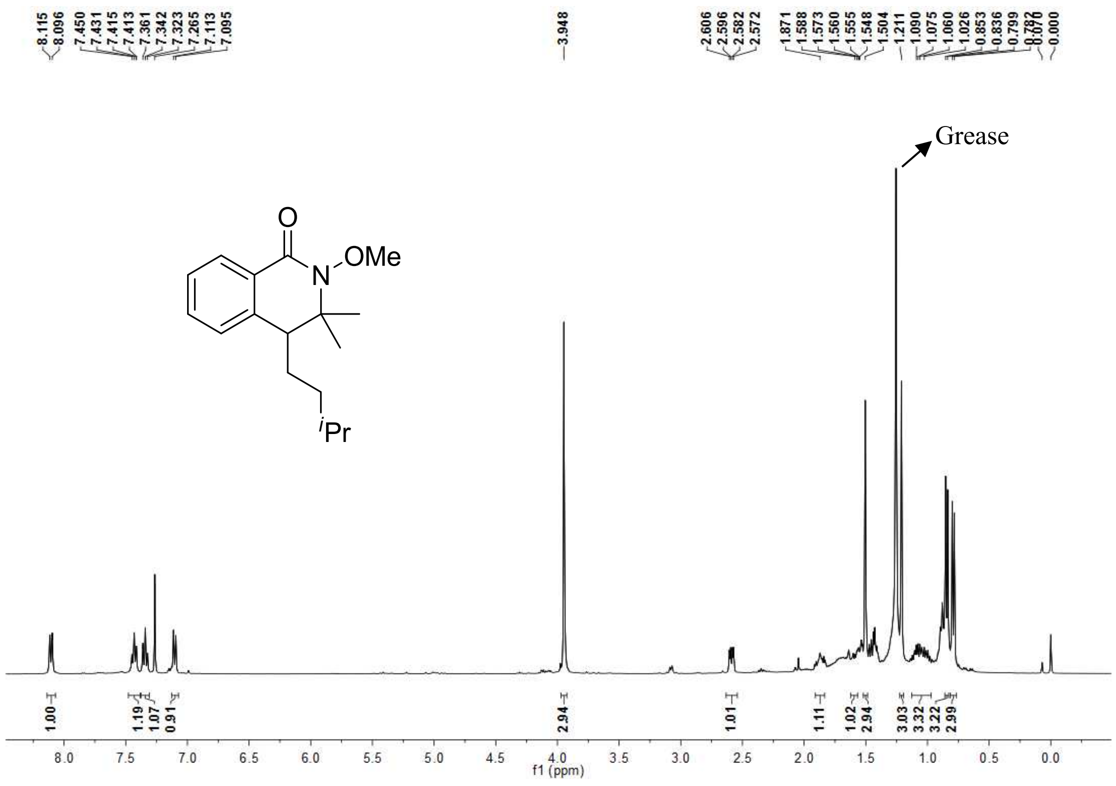
OMe
N
O
iPr
Grease
f1 (ppm)

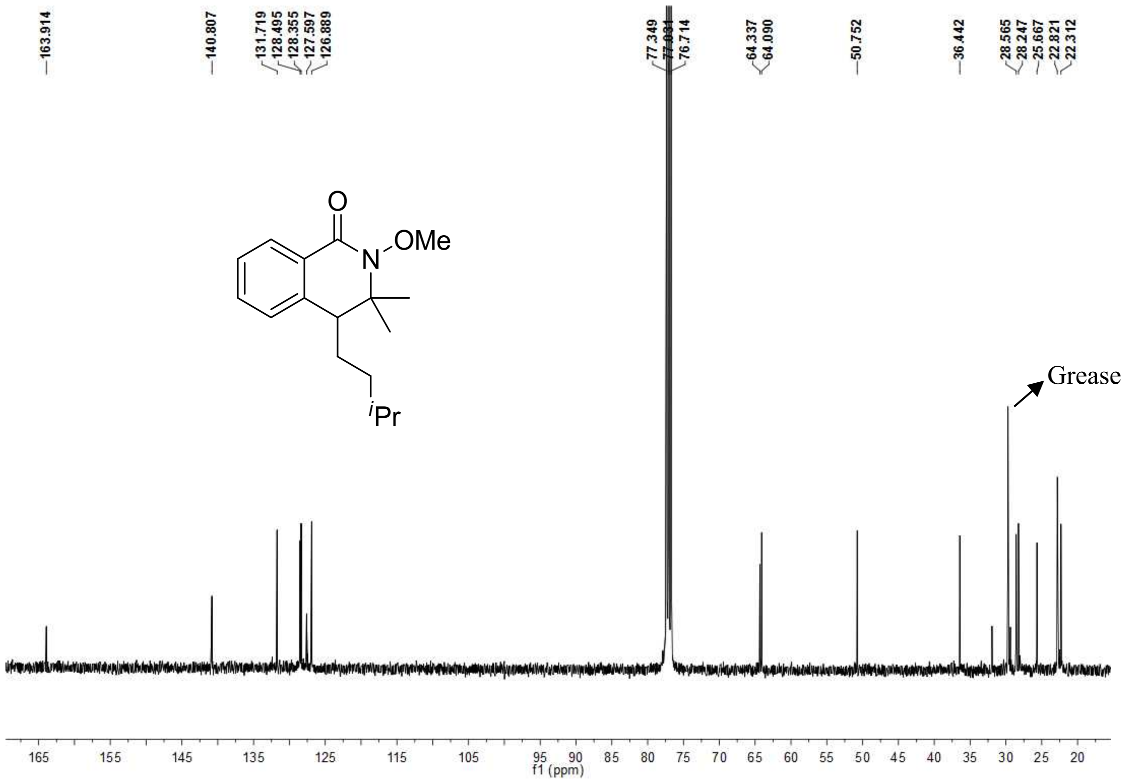
163.914
140.807
131.719
128.495
128.355
127.597
126.889
77.349
76.714
64.337
64.090
50.752
36.442
28.565
28.247
25.667
22.821
22.312
O
N
OMe
iPr
Grease
165
155
145
135
125
115
105
95
90
85
80
75
70
65
60
55
50
45
40
35
30
25
20
f1 (ppm)

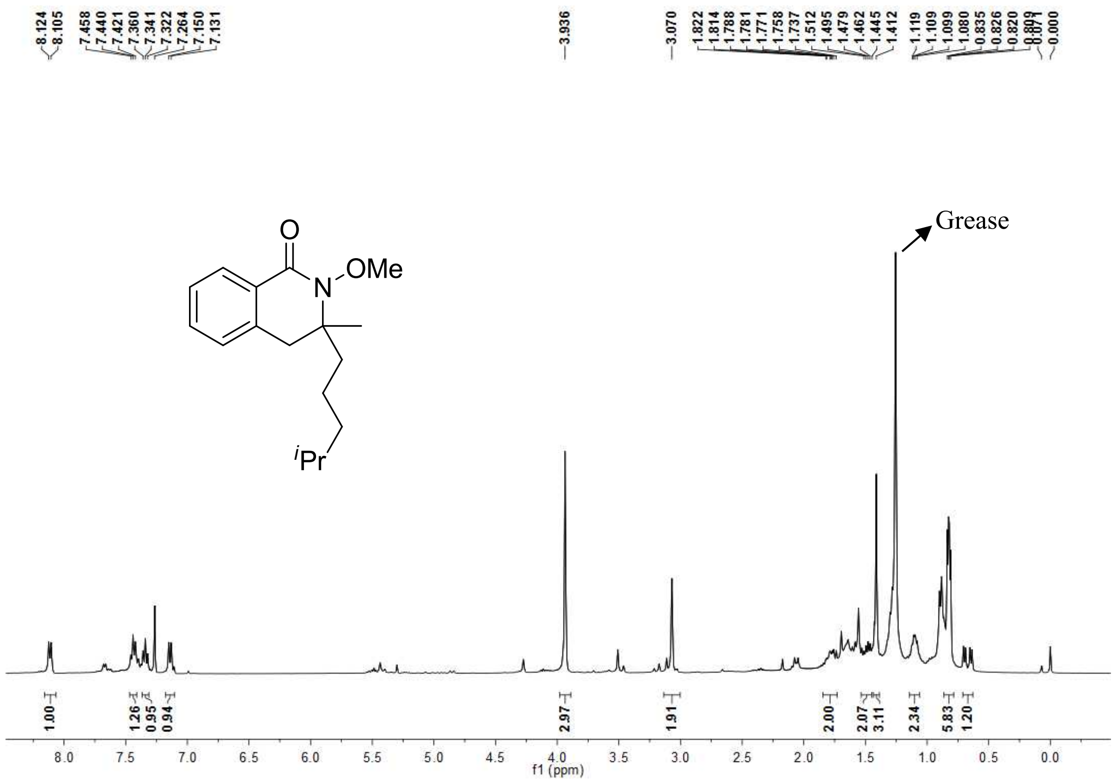
OMe
N
O
iPr
Grease
f1 (ppm)

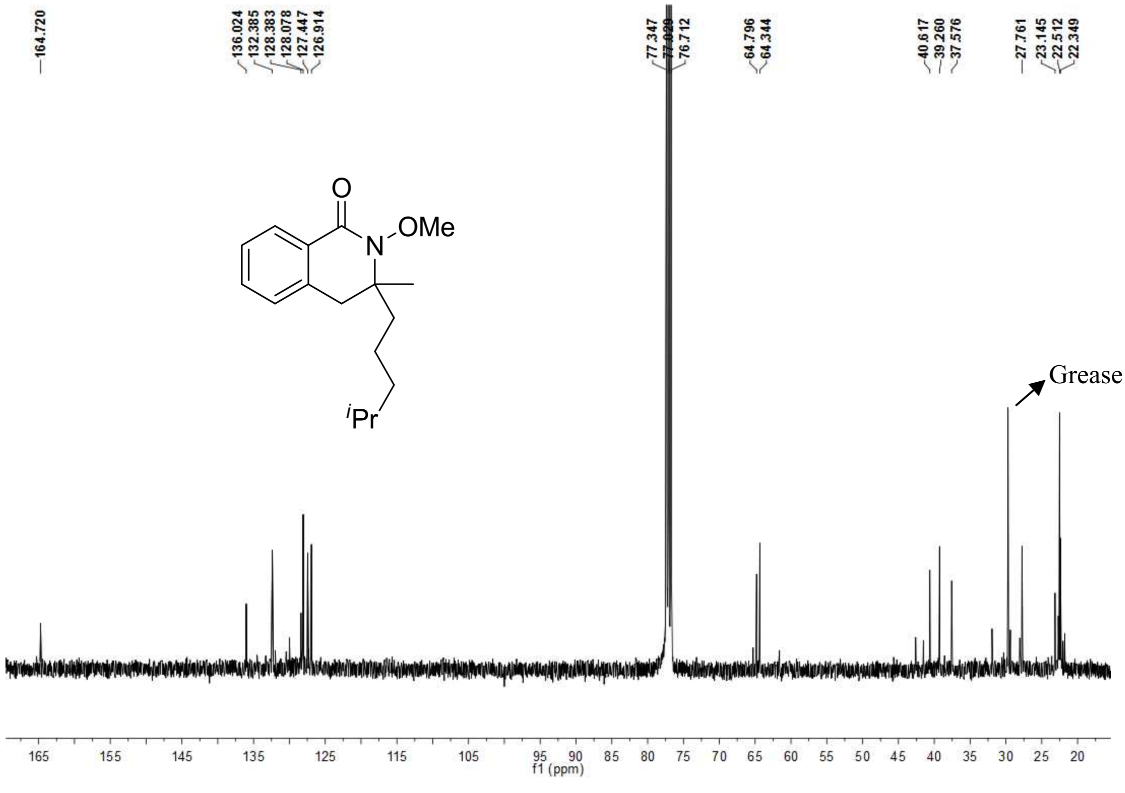
OMe
N
O
iPr
Grease
f1 (ppm)

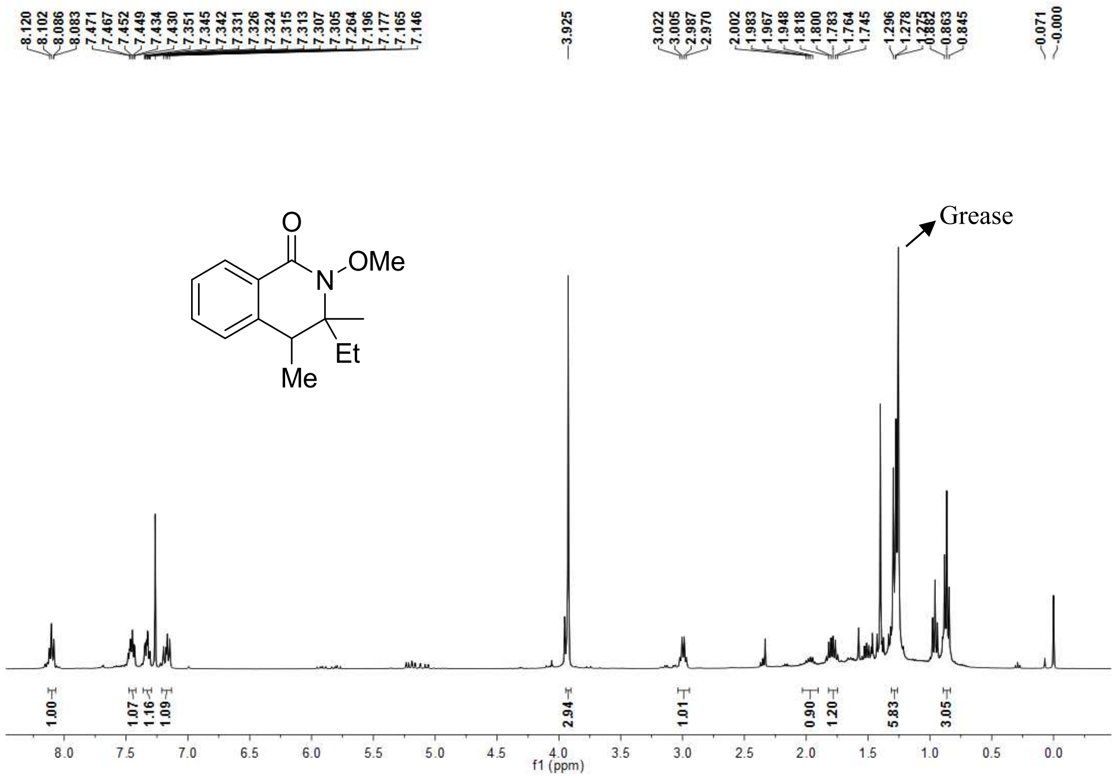
O
N
OMe
Et
Me
Grease
f1 (ppm)

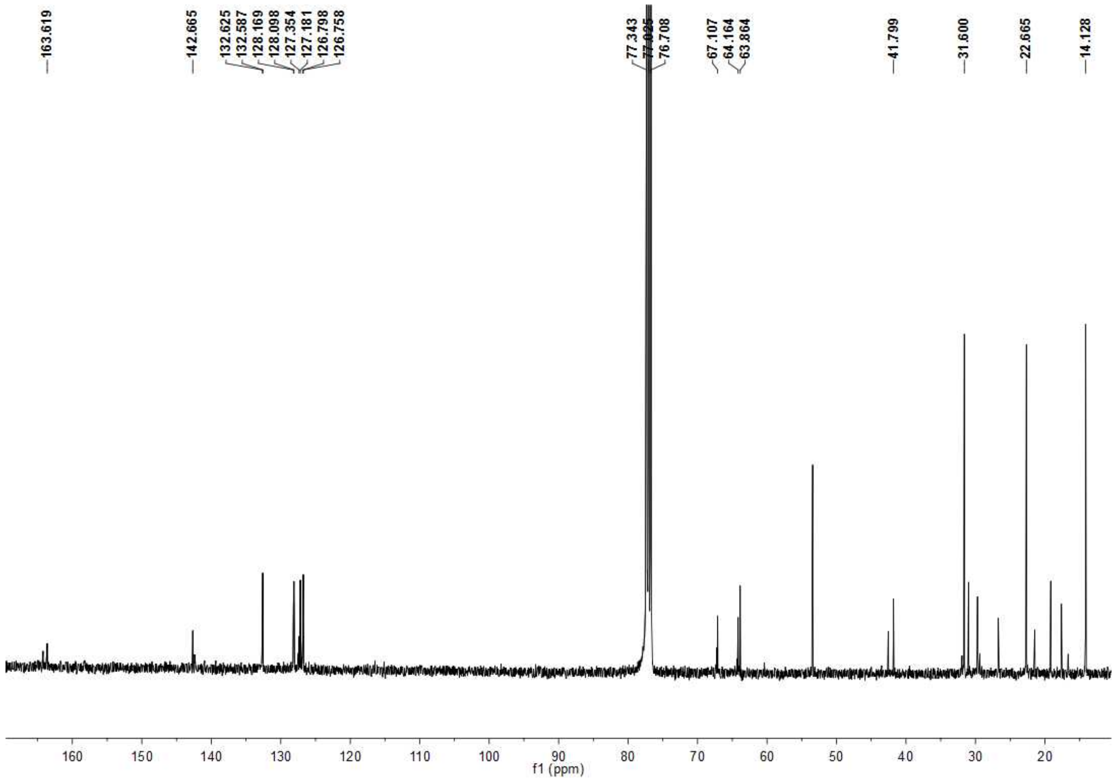
f1 (ppm)

X-ray Structure and Data of 18a

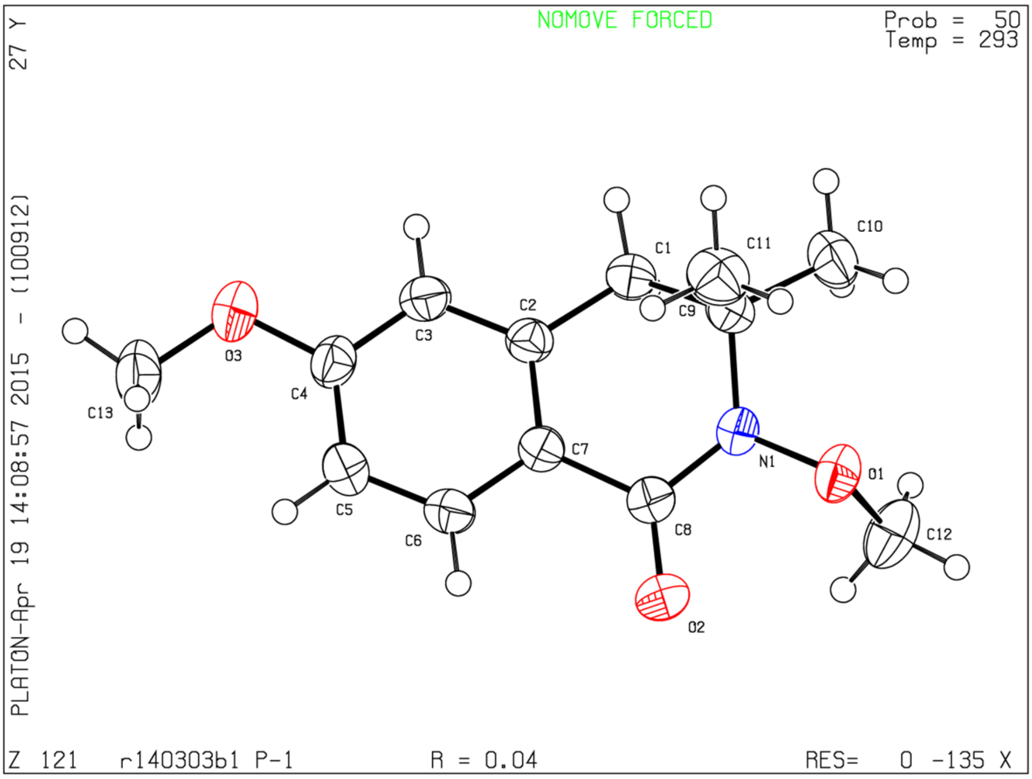

Table 1. Crystal data and structure refinement for 18a.

Identification code	**18a**
Empirical formula	C13 H17 N O3
Formula weight	235.28
Temperature	293(2) K
Wavelength	0.71073 A
Crystal system, space group	Triclinic, P-1
Unit cell dimensions	a = 7.6086(15) A alpha = 108.24(3) deg.
	b = 7.9394(16) A beta = 102.74(3) deg.
	c = 11.314(2) A gamma = 98.42(3) deg.
Volume	615.7(2) A^3
Z, Calculated density	2, 1.269 Mg/m^3
Absorption coefficient	0.090 mm^-1

F(000)	252
Crystal size	0.20 x 0.18 x 0.12 mm
Theta range for data collection	1.98 to 27.96 deg.
Limiting indices	-10<=h<=8, -10<=k<=10, -13<=l<=14
Reflections collected / unique	6345 / 2903 [R(int) = 0.0457]
Completeness to theta = 27.96	98.1 %
Absorption correction	Semi-empirical from equivalents
Max. and min. transmission	0.9893 and 0.9822
Refinement method	Full-matrix least-squares on F^2
Data / restraints / parameters	2903 / 0 / 158
Goodness-of-fit on F^2	1.034
Final R indices [I>2sigma(I)]	R1 = 0.0434, wR2 = 0.1028
R indices (all data)	R1 = 0.0813, wR2 = 0.1108
Largest diff. peak and hole	0.165 and -0.181 e.A^-3

Table 2. Atomic coordinates (x 10^4) and equivalent isotropic displacement parameters (A^2 x 10^3) for 18a.

U(eq) is defined as one third of the trace of the orthogonalized Uij tensor.

	x	y	z	U(eq)
O(1)	-1350(1)	-2814(1)	5674(1)	44(1)
O(2)	2047(1)	-2125(2)	7153(1)	59(1)
O(3)	3926(1)	5355(1)	11895(1)	53(1)
N(1)	-785(2)	-1452(2)	6923(1)	36(1)
C(1)	-1402(2)	1042(2)	8511(1)	39(1)
C(2)	643(2)	1693(2)	9164(1)	34(1)
C(3)	1418(2)	3256(2)	10244(1)	40(1)

C(4)	3316(2)	3777(2)	10838(1)	39(1)
C(5)	4466(2)	2714(2)	10345(1)	43(1)
C(6)	3697(2)	1148(2)	9271(1)	41(1)
C(7)	1805(2)	622(2)	8674(1)	35(1)
C(8)	1069(2)	-1089(2)	7519(1)	38(1)
C(9)	-1876(2)	-36(2)	7053(1)	37(1)
C(10)	-3932(2)	-967(2)	6546(2)	54(1)
C(11)	-1395(2)	1171(2)	6305(2)	54(1)
C(12)	-1873(2)	-4572(2)	5765(2)	62(1)
C(13)	5856(2)	5899(2)	12561(2)	61(1)

Table 3. Bond lengths [A] and angles [deg] for 18a.

O(1)-N(1)	1.4110(14)
O(1)-C(12)	1.4359(18)
O(2)-C(8)	1.2260(15)
O(3)-C(4)	1.3644(17)
O(3)-C(13)	1.4260(19)
N(1)-C(8)	1.3666(17)
N(1)-C(9)	1.4823(16)
C(1)-C(2)	1.5003(19)
C(1)-C(9)	1.5316(19)
C(1)-H(1A)	0.9700
C(1)-H(1B)	0.9700
C(2)-C(3)	1.378(2)
C(2)-C(7)	1.3983(18)
C(3)-C(4)	1.390(2)
C(3)-H(3)	0.9300

C(4)-C(5)	1.3874(18)
C(5)-C(6)	1.376(2)
C(5)-H(5)	0.9300
C(6)-C(7)	1.3859(19)
C(6)-H(6)	0.9300
C(7)-C(8)	1.486(2)
C(9)-C(11)	1.520(2)
C(9)-C(10)	1.5240(19)
C(10)-H(10A)	0.9600
C(10)-H(10B)	0.9600
C(10)-H(10C)	0.9600
C(11)-H(11A)	0.9600
C(11)-H(11B)	0.9600
C(11)-H(11C)	0.9600
C(12)-H(12A)	0.9600
C(12)-H(12B)	0.9600
C(12)-H(12C)	0.9600
C(13)-H(13A)	0.9600
C(13)-H(13B)	0.9600
C(13)-H(13C)	0.9600
N(1)-O(1)-C(12)	109.84(11)
C(4)-O(3)-C(13)	117.65(12)
C(8)-N(1)-O(1)	113.54(10)
C(8)-N(1)-C(9)	123.50(12)
O(1)-N(1)-C(9)	113.22(9)
C(2)-C(1)-C(9)	113.37(11)
C(2)-C(1)-H(1A)	108.9
C(9)-C(1)-H(1A)	108.9
C(2)-C(1)-H(1B)	108.9
C(9)-C(1)-H(1B)	108.9

H(1A)-C(1)-H(1B)	107.7
C(3)-C(2)-C(7)	118.67(13)
C(3)-C(2)-C(1)	123.27(12)
C(7)-C(2)-C(1)	118.02(12)
C(2)-C(3)-C(4)	121.19(13)
C(2)-C(3)-H(3)	119.4
C(4)-C(3)-H(3)	119.4
O(3)-C(4)-C(5)	124.08(13)
O(3)-C(4)-C(3)	115.96(13)
C(5)-C(4)-C(3)	119.95(13)
C(6)-C(5)-C(4)	119.04(13)
C(6)-C(5)-H(5)	120.5
C(4)-C(5)-H(5)	120.5
C(5)-C(6)-C(7)	121.29(13)
C(5)-C(6)-H(6)	119.4
C(7)-C(6)-H(6)	119.4
C(6)-C(7)-C(2)	119.85(13)
C(6)-C(7)-C(8)	118.40(13)
C(2)-C(7)-C(8)	121.75(12)
O(2)-C(8)-N(1)	122.53(13)
O(2)-C(8)-C(7)	122.27(13)
N(1)-C(8)-C(7)	115.17(12)
N(1)-C(9)-C(11)	110.60(11)
N(1)-C(9)-C(10)	108.92(12)
C(11)-C(9)-C(10)	110.31(12)
N(1)-C(9)-C(1)	105.85(10)
C(11)-C(9)-C(1)	112.03(13)
C(10)-C(9)-C(1)	108.99(12)
C(9)-C(10)-H(10A)	109.5
C(9)-C(10)-H(10B)	109.5

H(10A)-C(10)-H(10B)	109.5
C(9)-C(10)-H(10C)	109.5
H(10A)-C(10)-H(10C)	109.5
H(10B)-C(10)-H(10C)	109.5
C(9)-C(11)-H(11A)	109.5
C(9)-C(11)-H(11B)	109.5
H(11A)-C(11)-H(11B)	109.5
C(9)-C(11)-H(11C)	109.5
H(11A)-C(11)-H(11C)	109.5
H(11B)-C(11)-H(11C)	109.5
O(1)-C(12)-H(12A)	109.5
O(1)-C(12)-H(12B)	109.5
H(12A)-C(12)-H(12B)	109.5
O(1)-C(12)-H(12C)	109.5
H(12A)-C(12)-H(12C)	109.5
H(12B)-C(12)-H(12C)	109.5
O(3)-C(13)-H(13A)	109.5
O(3)-C(13)-H(13B)	109.5
H(13A)-C(13)-H(13B)	109.5
O(3)-C(13)-H(13C)	109.5
H(13A)-C(13)-H(13C)	109.5
H(13B)-C(13)-H(13C)	109.5

Symmetry transformations used to generate equivalent atoms:

Table 4. Anisotropic displacement parameters (A^2 x 10^3) for 18a.

The anisotropic displacement factor exponent takes the form:

-2 pi^2 [h^2 a*^2 U11 + ... + 2 h k a* b* U12]

	U11	U22	U33	U23	U13	U12
O(1)	52(1)	37(1)	34(1)	5(1)	7(1)	3(1)
O(2)	48(1)	51(1)	61(1)	-4(1)	9(1)	23(1)
O(3)	53(1)	45(1)	41(1)	0(1)	3(1)	5(1)
N(1)	36(1)	32(1)	32(1)	5(1)	4(1)	7(1)
C(1)	34(1)	40(1)	41(1)	11(1)	12(1)	11(1)
C(2)	35(1)	35(1)	34(1)	13(1)	9(1)	11(1)
C(3)	42(1)	39(1)	39(1)	11(1)	11(1)	16(1)
C(4)	47(1)	33(1)	30(1)	8(1)	5(1)	7(1)
C(5)	35(1)	45(1)	42(1)	13(1)	3(1)	6(1)
C(6)	37(1)	44(1)	41(1)	12(1)	10(1)	16(1)
C(7)	35(1)	33(1)	34(1)	12(1)	7(1)	9(1)
C(8)	39(1)	35(1)	39(1)	11(1)	8(1)	11(1)
C(9)	32(1)	39(1)	40(1)	15(1)	9(1)	11(1)
C(10)	34(1)	61(1)	53(1)	10(1)	5(1)	7(1)
C(11)	58(1)	54(1)	57(1)	31(1)	14(1)	17(1)
C(12)	76(1)	35(1)	62(1)	9(1)	17(1)	-2(1)
C(13)	61(1)	53(1)	44(1)	7(1)	-8(1)	-2(1)

Table 5. Hydrogen coordinates (x 10^4) and isotropic displacement parameters (A^2 x 10^3) for 18a.

	x	y	z	U(eq)
H(1A)	-1954	2088	8639	47

H(1B)	-1946	277	8920	47
H(3)	657	3976	10580	48
H(5)	5738	3055	10736	51
H(6)	4461	428	8939	49
H(10A)	-4238	-1655	5633	80
H(10B)	-4649	-61	6688	80
H(10C)	-4205	-1772	6996	80
H(11A)	-102	1762	6642	80
H(11B)	-2117	2076	6397	80
H(11C)	-1665	439	5404	80
H(12A)	-865	-4767	6346	93
H(12B)	-2166	-5500	4921	93
H(12C)	-2939	-4623	6087	93
H(13A)	6222	4953	12849	91
H(13B)	6097	7000	13297	91
H(13C)	6547	6109	11986	91

Table 6. Torsion angles [deg] for 18a.

C(12)-O(1)-N(1)-C(8)	90.36(15)
C(12)-O(1)-N(1)-C(9)	-122.29(13)
C(9)-C(1)-C(2)-C(3)	150.56(13)
C(9)-C(1)-C(2)-C(7)	-31.53(18)
C(7)-C(2)-C(3)-C(4)	0.2(2)
C(1)-C(2)-C(3)-C(4)	178.14(14)
C(13)-O(3)-C(4)-C(5)	-2.7(2)
C(13)-O(3)-C(4)-C(3)	177.67(12)
C(2)-C(3)-C(4)-O(3)	179.64(13)

C(2)-C(3)-C(4)-C(5)	0.0(2)
O(3)-C(4)-C(5)-C(6)	-179.86(14)
C(3)-C(4)-C(5)-C(6)	-0.2(2)
C(4)-C(5)-C(6)-C(7)	0.2(2)
C(5)-C(6)-C(7)-C(2)	0.0(2)
C(5)-C(6)-C(7)-C(8)	-179.81(14)
C(3)-C(2)-C(7)-C(6)	-0.2(2)
C(1)-C(2)-C(7)-C(6)	-178.21(13)
C(3)-C(2)-C(7)-C(8)	179.55(13)
C(1)-C(2)-C(7)-C(8)	1.5(2)
O(1)-N(1)-C(8)-O(2)	-16.68(19)
C(9)-N(1)-C(8)-O(2)	-160.20(13)
O(1)-N(1)-C(8)-C(7)	165.41(11)
C(9)-N(1)-C(8)-C(7)	21.90(19)
C(6)-C(7)-C(8)-O(2)	6.7(2)
C(2)-C(7)-C(8)-O(2)	-173.02(13)
C(6)-C(7)-C(8)-N(1)	-175.35(12)
C(2)-C(7)-C(8)-N(1)	4.9(2)
C(8)-N(1)-C(9)-C(11)	72.25(16)
O(1)-N(1)-C(9)-C(11)	-71.37(15)
C(8)-N(1)-C(9)-C(10)	-166.35(13)
O(1)-N(1)-C(9)-C(10)	50.03(15)
C(8)-N(1)-C(9)-C(1)	-49.29(17)
O(1)-N(1)-C(9)-C(1)	167.09(10)
C(2)-C(1)-C(9)-N(1)	51.04(16)
C(2)-C(1)-C(9)-C(11)	-69.59(15)
C(2)-C(1)-C(9)-C(10)	168.05(12)

Symmetry transformations used to generate equivalent atoms:
